# $C_2$ generalization of the van Diejen model from the minimal $(D_5,D_5)$ conformal matter

**DOI:** 10.1007/s11005-023-01714-7

**Published:** 2023-09-05

**Authors:** Belal Nazzal, Anton Nedelin

**Affiliations:** 1 Department of Physics, Technion, 32000 Haifa, Israel; 2 Section de Mathématiques, Université de Genève, 1211 Genève 4, Switzerland

**Keywords:** Elliptic Integrable Systems, Supersymmetric Field Theories, Superconformal Index, van Diejen model, Matrix Models, 81T30, 81T32, 37J35

## Abstract

We study superconformal indices of 4*d* compactifications of the 6*d* minimal $(D_{N+3},D_{N+3})$ conformal matter theories on a punctured Riemann surface. Introduction of supersymmetric surface defect in these theories is done at the level of the index by the action of the finite difference operators on the corresponding indices. There exist at least three different types of such operators according to three types of punctures with $A_N, C_N$ and $\left( A_1\right) ^N$ global symmetries. We mainly concentrate on $C_2$ case and derive explicit expression for an infinite tower of difference operators generalizing the van Diejen model. We check various properties of these operators originating from the geometry of compactifications. We also provide an expression for the kernel function of both our $C_2$ operator and previously derived $A_2$ generalization of van Diejen model. Finally, we also consider compactifications with $A_N$-type punctures and derive the full tower of commuting difference operators corresponding to this root system generalizing the result of our previous paper.

## Introduction

Intricate connection between supersymmetric gauge theories and integrable systems plays an important role in modern theoretical physics and mathematics. From physics point of view whenever some sector of the gauge theory is related to an integrable system, observables in this sector can be computed using rich variety of integrability techniques. Canonical example of this situation is the integrability of ${\mathcal {N}}=4$ super Yang–Mills (SYM) theory [1, 2]. In particular, it connects calculation of the planar scaling dimensions of ${\mathcal {N}}=4$ SYM to the integrable system of spin chains and allows to compute these scaling dimensions at arbitrary coupling. On the other hand, exploration of such connections can also shed light on some questions about integrable systems and even lead to construction of new classes of such systems making this kind of studies interesting from mathematics point of view.

In our work, we are exploring a particular class of connections between six-dimensional (1, 0) superconformal field theories and elliptic quantum mechanics Hamiltonians in the spirit of Bethe/gauge correspondence of Nekrasov and Shatashvili [3–6]. In particular, we consider four-dimensional theories with four supercharges obtained by compactification of a 6*d* SCFT on a punctured Riemann surface. In order for an integrable model to emerge in this setting, we have to introduce surface defects into our 4*d* theory and study its superconformal index.

This construction was first established in the context of compactifications of 6*d* (2, 0) of ADE type [7, 8]. In particular, the corresponding 4*d* superconformal indices with the defect were found to be closely related to the Ruijsenaars–Schneider (RS) elliptic analytic finite difference operators (A$\Delta$Os). Later these results were extended to many other cases: class $S_k$ 4*d* theories [9], compactifications of $A_2$ and $D_4$ minimal 6*d* SCFTs [10] and compactifications of rank one E-string theories [11]. In some of these cases, the obtained A$\Delta$Os were already known in the literature. For example in E-string compactifications, *van Diejen (vD) model* [12, 13] was observed. But in some cases operators were previously unknown as in the case of the minimal 6*d* SCFTs compactifications. Study of such novel operators constitutes an interesting field of research with some initial steps already taken in this direction [14, 15].

From the point of view of physics, the connection of superconformal indices in the presence of defects with the integrable quantum mechanics Hamiltonians allows one to bootstrap index of an arbitrary theory obtained by compactifying corresponding 6*d* theory on a punctured Riemann surface. In particular, if we know the eigenfunctions of the corresponding A$\Delta$O, we can compute the index of any theory obtained in such compactifications including non-Lagrangian theories for which there are no other methods of computing the index. As mentioned previously, the first setting where these ideas were tested is 4*d* class-S theories obtained in the compactifications of 6*d* (2, 0) theory [7]. In this case, the corresponding integrable system was given by elliptic RS model. Eigenfunctions of the elliptic RS Hamiltonians are not known in general, but some of their limits are well studied in the mathematical literature. These limits were used in order to prove previously established relations [16–18] of superconformal indices with the Macdonald and Schur polynomials allowing one to compute indices of class S non-Lagrangian theories.

The construction outlined above relies on the intermediate 5*d* layer. For things to work there should exist an effective 5*d* gauge theory obtained by compactifying original 6*d* SCFT on a circle with a choice of holonomies for its global symmetries. In particular, different 5*d* compactifications lead to different types of punctures on the Riemann surfaces used to obtain 4*d* theories. One of the interesting problems related to this fact is the compilation of the dictionary between known compactifications of various 6*d* SCFTs and elliptic integrable systems. In our previous paper [19], we have considered compactifications of the 6*d* minimal $(D_{N+3},D_{N+3})$ conformal matter theories [20, 21]. In particular, we have derived A$\Delta$Os corresponding to the intermediate 5*d* $\textrm{SU}(N+1)$ gauge theory or equivalently the $A_N$-type puncture on the compactification surface. These elliptic A$\Delta$Os appeared to be previously unknown $A_N$ generalizations of vD model. On the other hand, there are at least two more 5*d* effective descriptions of 6*d* SCFTs corresponding to $\textrm{USp}(2N)$ and $\textrm{SU}(2)^N$ gauge theories giving rise to the punctures with the same $C_N$ and $(A_1)^{\otimes N }$ global symmetries. These two descriptions should lead to additional higher-rank generalizations of the vD model.

In our present paper, we follow this line of research and closely study compactifications of the minimal $(D_{N+3},D_{N+3})$ conformal matter theory on a Riemann surface with the $C_N$-type punctures. In particular, we concentrate on the next to simplest case of $N=2$^1^ and derive corresponding infinite tower of A$\Delta$Os. We also devote part of the paper to the study and proof of some remarkable properties of these operators that follow from the geometry of corresponding compactifications. In particular, we prove the novel kernel property for two operators of different type but same rank. We also discuss commutation property of obtained operators.

The paper is organized as follows. In Sect. 2, we review our previous results for $A_N$-type operators. In addition to the previous results, we also derive full tower of such A$\Delta$Os which was not obtained previously. In Sect. 3, we derive in details novel generalization of vD operators corresponding to $C_2$ root system.^2^ In Sect. 4, we discuss properties of derived $C_2$ A$\Delta$Os. In particular we pay special attention to a new kernel function for simultaneously $A_2$ and $C_2$ operators and give fully analytic proof of the corresponding kernel equation. We also briefly discuss commutation relations and check them perturbatively in expansion. In Sect. 5, we briefly summarize our results and discuss plans for the future research further developing these results. Finally, the paper has a number of appendices collecting useful formulas as well as technical details of various calculations in our paper.

## $A_N$ operators

In this section, we will briefly review derivation of the $A_N$ generalization of the van Diejen operator. So-called basic version^3^ of this operator has been derived in our previous paper [19]. Here, we will extend this result to the full tower of operators.

**Fig. 1:**
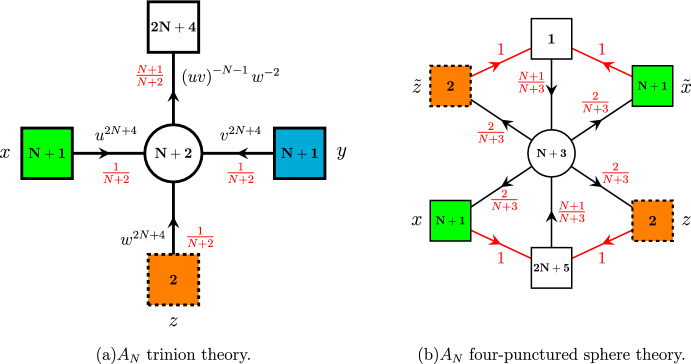
**a** $A_N$ three-punctured sphere with two maximal and one minimal puncture. **b** $A_N$ four-punctured sphere obtained by S-gluing two three-punctured spheres

Just as in [19] in order to derive A$\Delta$O, we start with the 4*d* three-punctured sphere theory which was first introduced in [21]. This theory is obtained in the compactification of the 6*d* minimal $(D_{N+3},D_{N+3})$ conformal matter on a sphere with two maximal $\textrm{SU}(N+1)$ punctures and one minimal $\textrm{SU}(2)$ puncture. The quiver of this theory is shown in Fig. 1. In addition to the global symmetry, the punctures are characterized by the moment map operators. In particular, both $\textrm{SU}(N+1)$ maximal and $\textrm{SU}(2)$ minimal punctures are characterized by $(2N+4)$ mesonic and 2 baryonic moment maps:

2.1 $$\begin{aligned} M_u= & {} \mathbf{N+1}^x\otimes \left( \mathbf{2N+4}_{u^{N+3}v^{-N-1}w^{-2}}\oplus \textbf{1}_{\left( uv^{N+1} \right) ^{2N+4}}\right) \oplus \overline{ \mathbf{N+1} }^x\otimes \textbf{1}_{\left( u^N w^2\right) ^{2N+4}}, \nonumber \\ M_v= & {} \mathbf{N+1}^y\otimes \left( \mathbf{2N+4}_{v^{N+3}u^{-N-1}w^{-2}}\oplus \textbf{1}_{\left( vu^{N+1} \right) ^{2N+4}}\right) \oplus \overline{ \mathbf{N+1} }^y\otimes \textbf{1}_{\left( v^N w^2\right) ^{2N+4}}, \nonumber \\ M_w= & {} \textbf{2}^z\otimes \left( \mathbf{2N+4}_{\left( uvw^{-2} \right) ^{-N-1}}\oplus \textbf{1}_{\left( wv^{N+1}\right) ^{2N+4}} \oplus \textbf{1}_{\left( wu^{N+1}\right) ^{2N+4}}\right) , \end{aligned}$$

where $a_i,\, u,\, v,\, w$ are fugacities of the Cartans of the 6*d* global $\textrm{SO}(4N+8)$ symmetry. Subscripts of the moment maps written above denote their charges w.r.t. to these symmetries.

Further *S*-gluing two such trinions along the maximal punctures, we can obtain four-punctured sphere with zero flux two maximal and two minimal punctures. To obtain the corresponding 4*d* gauge theory, we should just take two copies of trinion theories shown in Fig. 1 and then identify and gauge corresponding global symmetries of the maximal punctures. Here and everywhere else in the paper, all operations with gauge theories are expressed in terms of the superconformal indices. In this language, the gluing procedure takes the following form:

2.2 $$\begin{aligned} K_4^A(x,{\tilde{x}},z,{\tilde{z}})=\kappa _N\oint \prod \limits _{i=1}^N\frac{dy_i}{2\pi i y_i} \prod \limits _{i\ne j}^{N+1}\frac{1}{\Gamma _e\left( \frac{y_i}{y_j}\right) }{\bar{K}}_3^{A}({\tilde{x}},y,{\tilde{z}})K_3^{A}(x,y,z).\qquad \end{aligned}$$

where $K^{A}_3(x,y,z)$ is the index of the trinion with *y* being fugacity of the global $\textrm{SU}(N+1)$ symmetry of the puncture we glue along and ${\bar{K}}_3^{A}$ is the index of the conjugated trinion. Finally, $\kappa _N$ is the usual constant given by:

2.3 $$\begin{aligned} \kappa _N\equiv \frac{\left( q;q\right) _\infty ^{N}\left( p;p\right) _\infty ^{N}}{\left( N+1\right) !}. \end{aligned}$$

Performing this *S*-gluing operation, we obtain the four-punctured sphere theory shown in Fig. 1 with the corresponding superconformal index specified in (B.1). This theory was previously obtained by us in [19] to derive basic $A_N$ A$\Delta$Os. Now in order to derive the operator, we should close two minimal punctures of this four-punctured sphere. To do it, we should break the global symmetry of the puncture. This can be achieved by giving a non-trivial vev $\langle \partial _{12}^L \partial _{34}^K M\rangle \ne 0$ to the derivatives of one of the moment map operators. When we close punctures with at least one of *K* or *L* not equal to zero, i.e., vev is space-time dependent, we effectively insert defect into the theory [7, 22]. At the level of the superconformal index, closing the puncture amounts to giving a corresponding weight to the fugacity of the puncture’s global symmetry. Once we do it, we hit a pole of the index. Then, computation of the residue of this pole results in the superconformal index of the IR theory that the UV theory flowed to due to the introduction of the vev. In our case, we choose to close $\textrm{SU}(2)_z$ minimal puncture with the defect and $\textrm{SU}(2)_{{\tilde{z}}}$ puncture without, i.e., choosing $L=K=0$ in the vev. In particular, let’s say that we are going to compute A$\Delta$O acting on the puncture with the moment maps of charges ${\tilde{h}}_i$ and an overall $\textrm{U}(1)$ charge

2.4 $$\begin{aligned} {\tilde{h}}\equiv \prod \limits _{i=1}^{2N+6}{\tilde{h}}_i. \end{aligned}$$

For example, the moment maps of the $\textrm{SU}(N+1)_x$ puncture of the trinion shown in Fig. 1 correspond to $M_u$ operators specified in (2.1) and have the following charges:

2.5 $$\begin{aligned}{} & {} {\tilde{h}}_i = u^{N+3}v^{-N-1}w^{-2}a_i,\, \quad i=1,\dots ,2N+4, \quad \prod _{i=1}^{2N+4}a_i=1, \nonumber \\{} & {} {\tilde{h}}_{2N+5} = \left( uv^{N+1}\right) ^{2N+4},\,\quad {\tilde{h}}_{2N+6}= \left( u^{N} w^{2}\right) ^{-2N-4},\quad {\tilde{h}}=\left( uw^{-1}\right) ^{8(N+2)}, \nonumber \\ \end{aligned}$$

where we have flipped the charge ${\tilde{h}}_{2N+6}$ of the last moment map since this parametrization will be more natural for our operators and in this case all of the moment maps of the maximal puncture transform in the fundamental representation of $\textrm{SU}(N+1)_x$. We can now notice from (2.1) that charges of the minimal puncture moment maps are related to the charges of the moment maps of the maximal punctures by simple relation

2.6 $$\begin{aligned} {\tilde{h}}^{\textrm{SU}(2)}_i=\left( {\tilde{h}}^{\textrm{SU}(N+1)}_i\right) ^{-1}\left( {\tilde{h}}^{\textrm{SU}(N+1)}\right) ^{\frac{1}{4}}, \end{aligned}$$

So we can express everything in terms of only the moment maps of the maximal puncture we act on. Now, assume we give vevs to the moment maps (mesonic or baryonic) with charges ${\tilde{h}}_i^{-1}{\tilde{h}}^{1/4}$ of both $\textrm{SU}(2)_z$ and $\textrm{SU}(2)_{{\tilde{z}}}$. Moment maps we use should be the same in order to keep total flux of the 6*d* global symmetries zero. At the level of the index calculations, it corresponds to computing the residue of the index of the four-punctured sphere theory at the pole

2.7 $$\begin{aligned} z= & {} Z_{i;L,M}^*=(pq)^{-\frac{1}{2}} {\tilde{h}}_i{\tilde{h}}^{-\frac{1}{4}} q^{-M} p^{-L}, \;\;\;\;\;\;\;\nonumber \\ {\tilde{z}}= & {} {\tilde{Z}}^*_{i;0,0}=(pq)^{-\frac{1}{2}} {\tilde{h}}_i^{-1}{\tilde{h}}^{\frac{1}{4}} q^{-{\tilde{M}}} p^{-{\tilde{L}}}, \end{aligned}$$

where $L,M,{\tilde{L}},{\tilde{M}}$ are positive integers corresponding to the powers of derivatives inside the vev. As we mentioned previously, it is enough to introduce defect only for one of the two punctures. Hence, we choose ${\tilde{M}}={\tilde{L}}=0$ and keep *L*, *M* general. Then, we compute corresponding residues of the index of the four-punctured sphere theory and obtain theory for the tube with two maximal $\textrm{SU}(N+1)$ punctures and a codimension-two defect. Its superconformal index is given by^4^:

2.8 $$\begin{aligned} K_{(2;i;L,M)}^A(x,{\tilde{x}})\sim \textrm{Res}_{z\rightarrow Z^*_{i;L,M},{\tilde{z}}\rightarrow {\tilde{Z}}^*_{i;0,0}}K_4^A(x,{\tilde{x}},z,{\tilde{z}}) \end{aligned}$$

Finally in order to obtain desired A$\Delta$O, we glue our tube with the defect to an arbitrary Riemann surface with maximal $\textrm{SU}(N+1)_{{\tilde{x}}}$ puncture. As the result of this gluing, we expect to obtain action of a finite difference operator on the index ${{\mathcal {I}}}({\tilde{x}})$ of this 4*d* ${\mathcal {N}}=1$ theory:

2.9 $$\begin{aligned} {{\mathcal {O}}}^{(A_N;h_{k};L,M)}_x\cdot {{\mathcal {I}}}(x)=\kappa _N\oint \prod \limits _{j=1}^N\frac{d{\tilde{x}}_j}{2\pi i{\tilde{x}}_{j}}\prod \limits _{i\ne j}^{N+1}\frac{1}{\Gamma _e\left( \frac{{\tilde{x}}_i}{{\tilde{x}}_j}\right) }K_{(2;i;L,M)}^A(x,\,{\tilde{x}}) {{\mathcal {I}}}({\tilde{x}}) \nonumber \\ \end{aligned}$$

Details of all the calculations summarized above are given in Appendix B. They result in the following operator:
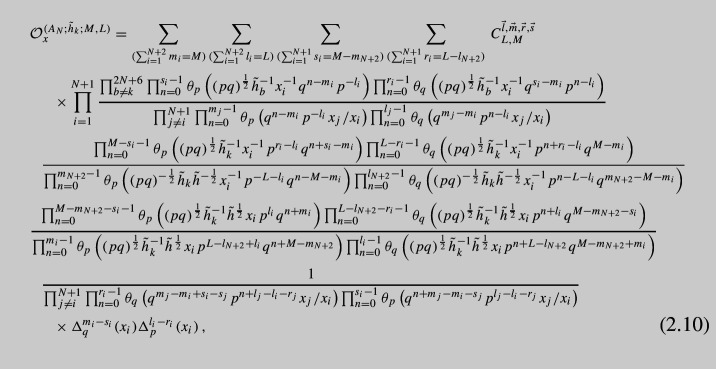


where $C_{L,M}^{\vec {l},\vec {m},\vec {r},\vec {s}}$ are *x*-independent constant factors given by,
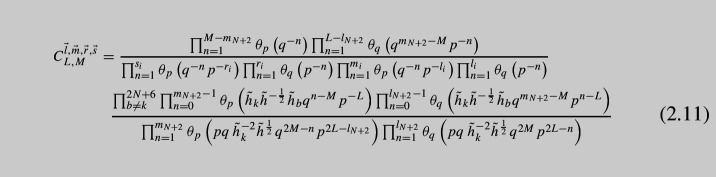


Also $\Delta$s are shift operators defined as follows:

2.12 $$\begin{aligned} \Delta ^m_a(x_i)f(x)\equiv f\left( x_i\rightarrow a^mx_i\right) , \quad a=q,p. \end{aligned}$$

The operator contains all shifts of the form $q^{m_i}p^{l_i}x_i$ where $\vec {m}$ and $\vec {l}$ are all possible partitions of length $N+1$ of *M* and *L* correspondingly. At each level, i.e., fixed *M* and *L*, there are $2N+6$ operators due to $2N+6$ moment maps with the charges ${\tilde{h}}_k{\tilde{h}}^{-\frac{1}{4}}$. There are also $2N+6$ other operators obtained by giving vevs to the flipped moment maps of the charge ${\tilde{h}}_k^{-1}{\tilde{h}}^{\frac{1}{4}}$. They have similar form and properties so we do not present them here. All the operators should commute with each other, and we checked this in expansion in *p*, *q* for a few of the simplest cases. Now, we will refer to the case $M=1$ and $L=0$, or vice versa, as *basic operators*. These basic operators for $A_N$ generalizations of vD model were derived by us previously in [19]. The operators above also reproduce our previous results when we fix $M=1,\, L=0$ or $M=0,\, L=1$. Further in our paper, we will also need the basic operator obtained by closing flipped moment maps. This operator can be found in [19] and has the following form: 
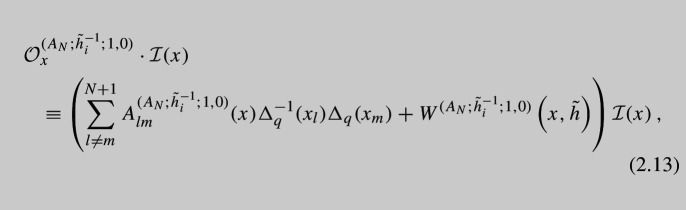


where the shift part of this operator is given by

2.14 $$\begin{aligned} A^{(A_N;{\tilde{h}}_i^{-1};1,0)}_{lm}(x)= & {} \frac{\prod \limits _{j=1}^{2N+6}\theta _p\left( (pq)^{\frac{1}{2}} {\tilde{h}}_j^{-1}x_l^{-1}\right) }{\theta _p\left( \frac{x_m}{x_l}\right) \theta _p\left( q\frac{x_m}{x_l}\right) }\nonumber \\{} & {} \prod \limits _{k\ne m\ne l}^{N+1}\frac{\theta _p\left( (pq)^{\frac{1}{2}}{\tilde{h}}_i^{-1}x_k^{-1}\right) \theta _p\left( (pq)^{\frac{1}{2}}{\tilde{h}}_i^{-1}{\tilde{h}}^{1/2} x_k\right) }{\theta _p\left( \frac{x_k}{x_l}\right) \theta _p\left( \frac{x_m}{x_k}\right) },\nonumber \\ \end{aligned}$$

and the constant part is given by:

2.15 $$\begin{aligned} W^{(A_N;{\tilde{h}}_i^{-1};1,0)}(x,{\tilde{h}})= & {} \frac{\prod \limits _{j\ne i}^{2N+6}\theta _p\left( q^{-1}{\tilde{h}}_i{\tilde{h}}_j{\tilde{h}}^{-1/2}\right) }{\theta _p\left( q^{-2}{\tilde{h}}_i^{2}{\tilde{h}}^{-1/2}\right) } \prod \limits _{k=1}^{N+1}\frac{\theta _p\left( (pq)^{\frac{1}{2}}{\tilde{h}}_i^{-1} x_k^{-1}\right) }{\theta _p\left( (pq)^{-\frac{1}{2}}{\tilde{h}}_i{\tilde{h}}^{-1/2}q^{-1}x_k^{-1}\right) } \nonumber \\{} & {} +\sum \limits _{m=1}^{N+1}\frac{\prod \limits _{j\ne i}^{2N+6}\theta _p\left( (pq)^{\frac{1}{2}}{\tilde{h}}_jx_m\right) }{\theta _p\left( (pq)^{\frac{1}{2}}{\tilde{h}}_i^{-1}{\tilde{h}}^{1/2}q x_m\right) } \nonumber \\{} & {} \prod \limits _{k\ne m}^{N+1}\frac{\theta _p\left( (pq)^{\frac{1}{2}}{\tilde{h}}_{i}^{-1}{\tilde{h}}^{1/2}x_k\right) \theta _p\left( (pq)^{\frac{1}{2}}{\tilde{h}}_i^{-1}x_k^{-1}\right) }{\theta _p\left( q^{-1}\frac{x_k}{x_m}\right) \theta _p\left( \frac{x_m}{x_k}\right) }. \end{aligned}$$

This constant part is elliptic function in each $x_i$ variable with periods 1 and *p*. It has poles in the fundamental domain at the following positions:

2.16 $$\begin{aligned} x_i=q^{\pm 1}x_r,\quad x_i=sq^{\pm \frac{1}{2}}P_i^{-\frac{1}{2}},\quad x_i=sq^{\pm \frac{1}{2}}p^{\frac{1}{2}}P_i^{-\frac{1}{2}},\quad s=\pm 1, \end{aligned}$$

where we defined

2.17 $$\begin{aligned} P_i\equiv \prod \limits _{j\ne i}^N x_j. \end{aligned}$$

From expression (2.15), it looks like there are extra poles in the constant part, but careful examination shows that residues at these values of *x*’s are zero so there are no real poles there. At the poles (2.16), we have the following residues:

2.18 $$\begin{aligned}&\textrm{Res}_{x_l=qx_r}W^{(A_N;{\tilde{h}}_i^{-1};1,0)}(x,{\tilde{h}})\nonumber \\&\quad =-\frac{qx_r}{\left( p;p\right) _\infty ^2\theta _p\left( q^{-1}\right) }\prod \limits _{j=1}^{2N+6}\theta _p\left( (pq)^{\frac{1}{2}}{\tilde{h}}_jx_r\right) \nonumber \\&\qquad \times \prod \limits _{k\ne l\ne r}^{N+1}\frac{\theta _p\left( (pq)^{\frac{1}{2}}{\tilde{h}}_i^{-1}{\tilde{h}}^{1/2}x_k\right) \theta _p\left( (pq)^{\frac{1}{2}}{\tilde{h}}_i^{-1}x_k^{-1}\right) }{\theta _p\left( q^{-1}\frac{x_k}{x_r}\right) \theta _p\left( \frac{x_r}{x_k}\right) } \nonumber \\&\textrm{Res}_{x_l=q^{-1}x_r}W^{(A_N;{\tilde{h}}_i^{-1};1,0)}(x,h)\nonumber \\&\quad =\frac{q^{-1}x_r}{\left( p;p\right) _\infty ^2\theta _p\left( q^{-1}\right) }\prod \limits _{j=1}^{2N+6}\theta _p\left( (pq)^{\frac{1}{2}}{\tilde{h}}_j^{-1}x_r^{-1}\right) \nonumber \\&\qquad \times \prod \limits _{k\ne l\ne r}^{N+1}\frac{\theta _p\left( (pq)^{\frac{1}{2}}{\tilde{h}}_i^{-1}{\tilde{h}}^{1/2}x_k\right) \theta _p\left( (pq)^{\frac{1}{2}}{\tilde{h}}_i^{-1}x_k^{-1}\right) }{\theta _p\left( q^{-1}\frac{x_r}{x_k}\right) \theta _p\left( \frac{x_k}{x_r}\right) } \nonumber \\&\textrm{Res}_{x_l=sq^{-\frac{1}{2}}P_l^{-\frac{1}{2}}}W^{(A_N;{\tilde{h}}_i^{-1};1,0)}(x,h)\nonumber \\&\quad =s\frac{q^{-\frac{1}{2}}P_l^{-\frac{1}{2} }}{2 \left( p;p\right) _\infty ^2\theta _p\left( q^{-1}\right) } \prod \limits _{j=1}^{2N+6}\theta _p\left( sp^{1/2}{\tilde{h}}_jP_l^{-1/2}\right) \nonumber \\&\qquad \times \prod \limits _{k\ne l}^N \frac{\theta _p\left( (pq)^{\frac{1}{2}}{\tilde{h}}_i^{-1}{\tilde{h}}^{1/2}x_k\right) \theta _p\left( (pq)^{\frac{1}{2}}{\tilde{h}}_i^{-1}x_k^{-1}\right) }{\theta _p\left( sq^{-1/2}P_l^{-1/2}x_k^{-1}\right) \theta _p\left( sq^{-1/2}P_l^{1/2}x_k\right) }\,, \nonumber \\&\textrm{Res}_{x_l=sq^{\frac{1}{2}}P_l^{-\frac{1}{2}}}W^{(A_N;{\tilde{h}}_i^{-1};1,0)}(x,{\tilde{h}})\nonumber \\&\quad =-s\frac{q^{\frac{1}{2}}P_l^{-\frac{1}{2} }}{2 \left( p;p\right) _\infty ^2\theta _p\left( q^{-1}\right) } \prod \limits _{j=1}^{2N+6}\theta _p\left( sp^{1/2}{\tilde{h}}_jP_l^{-1/2}\right) \nonumber \\&\qquad \times \prod \limits _{k\ne l}^N \frac{\theta _p\left( (pq)^{\frac{1}{2}}{\tilde{h}}_i^{-1}{\tilde{h}}^{1/2}x_k\right) \theta _p\left( (pq)^{\frac{1}{2}}{\tilde{h}}_i^{-1}x_k^{-1}\right) }{\theta _p\left( sq^{-1/2}P_l^{-1/2}x_k^{-1}\right) \theta _p\left( sq^{-1/2}P_l^{1/2}x_k\right) }\,, \nonumber \\&\textrm{Res}_{x_l=sp^{\frac{1}{2}}q^{-\frac{1}{2}}P_l^{-\frac{1}{2}}}W^{(A_N;{\tilde{h}}_i^{-1};1,0)}(x,{\tilde{h}})\nonumber \\&\quad =s\frac{q^{-\frac{1}{2}}p^{\frac{3}{2}}{\tilde{h}}^{-\frac{1}{2}} P_l^{\frac{1}{2} }}{2 \left( p;p\right) _\infty ^2\theta _p\left( q^{-1}\right) } \prod \limits _{j=1}^{2N+6}\theta _p\left( s{\tilde{h}}_jP_l^{-1/2}\right) \nonumber \\&\qquad \times \prod \limits _{k\ne l}^N \frac{\theta _p\left( (pq)^{\frac{1}{2}}{\tilde{h}}_i^{-1}{\tilde{h}}^{1/2}x_k\right) \theta _p\left( (pq)^{\frac{1}{2}}{\tilde{h}}_i^{-1}x_k^{-1}\right) }{\theta _p\left( sp^{-1/2}q^{-1/2}P_l^{-1/2}x_k^{-1}\right) \theta _p\left( sp^{1/2}q^{-1/2}P_l^{1/2}x_k\right) }\,, \nonumber \\&\textrm{Res}_{x_l=sp^{\frac{1}{2}}q^{\frac{1}{2}}P_l^{-\frac{1}{2}}}W^{(A_N;{\tilde{h}}_i^{-1};1,0)}(x,{\tilde{h}})\nonumber \\&\quad =-s\frac{q^{\frac{1}{2}}p^{\frac{3}{2}}{\tilde{h}}^{-\frac{1}{2}} P_l^{\frac{1}{2} }}{2 \left( p;p\right) _\infty ^2\theta _p\left( q^{-1}\right) }\prod \limits _{j=1}^{2N+6}\theta _p\left( s{\tilde{h}}_jP_l^{-1/2}\right) \nonumber \\&\qquad \times \prod \limits _{k\ne l}^N \frac{\theta _p\left( (pq)^{\frac{1}{2}}{\tilde{h}}_i^{-1}{\tilde{h}}^{1/2}x_k\right) \theta _p\left( (pq)^{\frac{1}{2}}{\tilde{h}}_i^{-1}x_k^{-1}\right) }{\theta _p\left( sp^{-1/2}q^{-1/2}P_l^{-1/2}x_k^{-1}\right) \theta _p\left( sp^{1/2}q^{-1/2}P_l^{1/2}x_k\right) }\,, \end{aligned}$$

These are completely general expressions for the whole family of $A_N$ operators.

## Derivation of $C_2$ operators

In this section, we will discuss derivation of the *C*-type rank-2 analytic finite difference operators A$\Delta$O. In our previous paper [19], we have already derived the basic operator for $A_N$ generalization of van Diejen model and the calculation of the full tower of these operators is summarized in Sect. 2. Unfortunately full $C_N$ generalization is still out of reach for us due to technical complications but we can concentrate on the study of rank-2 generalizations with $C_2$ root system.

We will derive $C_2$ operator corresponding to the insertion of the codimension-two defect due to the non-trivial vev of the holomorphic derivatives of the moment maps $\langle \partial _{\pm }^K M \rangle \ne 0$. To simplify our calculations of A$\Delta$O, we will start with the derivation of four- and three-punctured spheres with $C_2$-type punctures.

**Fig. 2:**
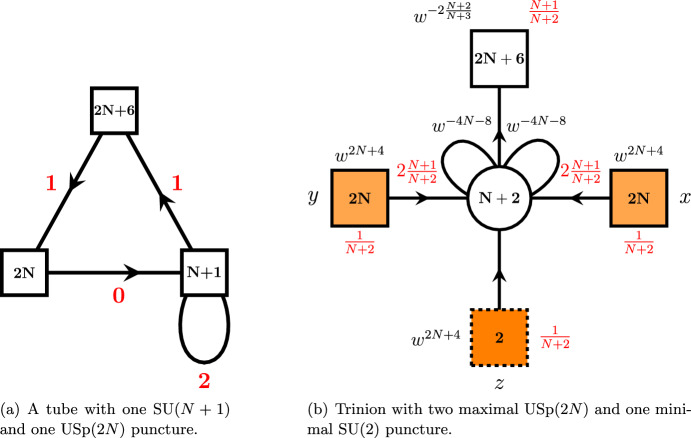
Tube and trinion with maximal $C_N$ punctures

The basic $C_N$ trinion was already derived by us in Appendix B.5 of [19] and is shown in Fig. 2b. Generalizations of this trinion to the cases of three maximal punctures and higher numbers of minimal punctures can also be found in [23]. In order to derive this trinion, we used a tube theory with one $\textrm{USp}(2N)$ and one $\textrm{SU}(N+1)$ maximal punctures shown in Fig. 2a. This theory was first discussed in [24]. In order to obtain trinion theory shown in Fig. 2b, we start with the three-punctured sphere with two maximal $\textrm{SU}(N+1)$ and one minimal $\textrm{SU}(2)$ punctures and glue two $A_NC_N$ tubes to the maximal punctures. The moment maps of the punctures of the resulting trinion are given by $(2N+6)$ mesons both for maximal and minimal punctures:

3.1 $$\begin{aligned} M_x= & {} \textbf{2N}_x\otimes \left( \mathbf{2N+6}\right) _{w^{2\frac{(N+2)^2}{N+3}}}, \qquad M_y=\textbf{2N}_y\otimes \left( \mathbf{2N+6}\right) _{w^{2\frac{(N+2)^2}{N+3}}}, \nonumber \\ M_z= & {} \textbf{2}_z\otimes \left( \mathbf{2N+6}\right) _{w^{2\frac{(N+2)^2}{N+3}}}, \end{aligned}$$

where *w* is one of the parameters of Cartans of the global $\textrm{SO}(4N+8)$ symmetry of the 6*d* minimal conformal matter theory. In general, we will use two types of parametrization of 6*d* global symmetry in this paper. First one is natural to use in compactifications with $C_N$-type punctures, and it is given by

3.2 $$\begin{aligned} {\tilde{a}}_i,\quad i=1,\dots ,2N+6,\quad \prod \limits _{i=1}^{2N+6}{\tilde{a}}_i=1, \quad w. \end{aligned}$$

Another parametrization is useful when we work with $A_N$ expressions and is given by:

3.3 $$\begin{aligned} a_i,\quad i=1,\dots ,2N+4,\quad \prod \limits _{i=1}^{2N+4}a_i=1, \quad u,v,w. \end{aligned}$$

These two parametrizations are related by the following map that we will often need in our calculations:

3.4 $$\begin{aligned} {\tilde{a}}_l= & {} \left( uv \right) ^{-N-1}w^{-\frac{2}{N+3}}a_l,~l=1,\dots ,2N+4, \nonumber \\ {\tilde{a}}_{2N+6}= & {} u^{2(N+1)(N+2)}w^{2\frac{N+2}{N+3}},\quad {\tilde{a}}_{2N+5}=v^{2(N+1)(N+2)}w^{2\frac{N+2}{N+3}}. \end{aligned}$$

Now in order to derive finite difference operators, we should proceed in the same way as in the case of $A_N$ operators summarized in Sect. 2. We start by taking two $C_N$ trinion theories ${{\mathcal {T}}}^{{\mathcal {A}}}_{x,y,z}$ where ${\mathcal {A}}$ is the nonzero flux of the trinion and *x*, *y*, *z* in the subscript are fugacities of the global symmetries of two maximal and one minimal punctures correspondingly. Then, we take an arbitrary ${{\mathcal {N}}}=1$ theory obtained in the compactification of the minimal $(D_{N+3},D_{N+3})$ conformal matter theory on the Riemann surface of genus *g* with *s* punctures denoted as ${{\mathcal {C}}}_{g,s}$ with at least one $\textrm{USp}(2N)_{{\tilde{x}}}$ maximal puncture. Gluing all three surfaces together along the maximal punctures results in a Riemann surface of the same genus and global symmetry fluxes but two extra minimal punctures. We will be performing all these operations at the level of superconformal indices. There gluing amounts to identifying global symmetries of the punctures we glue and gauging it. So for the indices we can write down the following identity after gluing:

3.5 $$\begin{aligned}{} & {} {{\mathcal {I}}}\left[ {{\mathcal {C}}}_{g,s}({\tilde{x}})\oplus {\bar{ {{\mathcal {T}}}}}^{{\mathcal {A}}}_{{\tilde{x}},y,z_1}\oplus {{\mathcal {T}}}^{{\mathcal {A}}}_{y,x,z_2} \right] \nonumber \\{} & {} \quad =\frac{\left( q;q\right) _\infty ^4\left( p;p\right) _\infty ^4}{2^4\cdot 2!\cdot 2!}\oint \prod \limits _{i=1}^2\frac{d{\tilde{x}}_i}{2\pi i {\tilde{x}}_i}\frac{dy_i}{2\pi i y_i} \prod \limits _{i=1}^N\frac{1}{\Gamma _e\left( y_i^{\pm 2}\right) }\nonumber \\{} & {} \qquad \times \prod \limits _{i<j}^N\frac{1}{\Gamma _e\left( y_i^{\pm 1}y_j^{\pm 1}\right) } \prod \limits _{i=1}^N\frac{1}{\Gamma _e\left( {\tilde{x}}_i^{\pm 2}\right) }\nonumber \\{} & {} \quad \qquad \prod \limits _{i<j}^N\frac{1}{\Gamma _e\left( {\tilde{x}}_i^{\pm 1}{\tilde{x}}_j^{\pm 1}\right) } K_3^{C}(x,y,z_2){\bar{K}}_3^{C}({\tilde{x}},y,z_1) {{\mathcal {I}}}\left[ {{\mathcal {C}}}_{g,s}({\tilde{x}})\right] , \end{aligned}$$

where ${{\mathcal {I}}}\left[ {{\mathcal {C}}}_{g,s}\right]$ is the index of a theory obtained by compactifications of 6*d* theory on the Riemann surface ${{\mathcal {C}}}_{g,s}$ and $K_3^{C}(x,y,z)$ is the index of the trinion theory shown in Fig. 2b, while ${\bar{K}}_3^{C}(x,y,z)$ is its conjugate. Definition and properties of the elliptic $\Gamma$-function $\Gamma _e\left( x\right)$ are given in Appendix A. Then, closing $\textrm{SU}(2)_z$ and $\textrm{SU}(2)_{{\tilde{z}}}$ minimal punctures with or without introduction of defects we can obtain finite difference operators. As discussed previously in order to close minimal punctures, we have to give certain weights to corresponding global symmetry fugacities $z_1$ and $z_2$. As a result, we get the following identity

3.6 $$\begin{aligned} \lim _{z_1\rightarrow Z_1^*,z_2\rightarrow Z_2^* } {{\mathcal {I}}}\left[ {{\mathcal {C}}}_{g,s}(x)\oplus {\bar{ {{\mathcal {T}}}}}^{{\mathcal {A}}}_{x,y,z_1}\oplus {{\mathcal {T}}}^{{\mathcal {A}}}_{y,{\tilde{x}},z_2} \right] \sim {{\mathcal {O}}}\cdot {{\mathcal {I}}}\left[ {{\mathcal {C}}}_{g,s}(x)\right] , \end{aligned}$$

where ${{\mathcal {O}}}$ is some finite difference operator, and in case we close punctures without introducing defects we just obtain the identity operator.

Here for convenience, we will not compute the full expression (3.5) directly right away. Just as for $A_N$ operators derived in Sect. 2, it appears to be much easier to perform calculation in a slightly different way. First we derive the index of the four-punctured sphere with zero flux, two maximal $\textrm{USp}(2N)$ and two minimal $\textrm{SU}(2)$ punctures. For this purpose, we perform *S*-gluing of two trinions ${{\mathcal {T}}}^{AC}_{x,y,z}$ with $\textrm{SU}(N+1)$ and $\textrm{USp}(2N)$ maximal punctures and $\textrm{SU}(2)$ minimal punctures each. This kind of trinions can be derived by appropriate gluing of $A_NC_N$ tube theory shown in Fig. 2a, to one of the maximal punctures of $A_N$ trinion introduced in [21]. Superconformal index of the resulting four-punctured sphere theory is given by:

3.7 $$\begin{aligned} K_4^C(x,{\tilde{x}},z,{\tilde{z}})=\kappa _N\oint \prod \limits _{i=1}^N\frac{dy_i}{2\pi i y_i} \prod \limits _{i\ne j}^{N+1}\frac{1}{\Gamma _e\left( \frac{y_i}{y_j}\right) } {\bar{K}}_3^{AC}({\tilde{x}},y,{\tilde{z}})K_3^{AC}(x,y,z). \end{aligned}$$

where $K^{AC}_3(x,y,z)$ is the index of the trinion with *y* being $\textrm{SU}(N+1)$ fugacity and $\kappa _N$ is the usual constant given in (2.3). Geometrically this operation is shown in Fig. 5a.

Next we close one of the two minimal punctures by giving nonzero vev to one of the $M_z$ moment maps given in (3.1). At the level of the index computations, this means that the corresponding global symmetry fugacity should be consistent with the vev, i.e., in our case we should fix ${\tilde{z}}$ fugacity to

3.8 $$\begin{aligned} {\tilde{z}}={\tilde{Z}}_{i;K,M}^*\equiv (pq)^{-\frac{1}{2}}w^{2\frac{(N+2)^2}{N+3}}{\tilde{a}}_i q^{-K}p^{-M}, \end{aligned}$$

where ${\tilde{a}}_i$ can be chosen arbitrary since all the expressions are symmetric w.r.t. permutations of ${\tilde{a}}_i$. Integers *K* and *M* correspond to an order of the derivative of the moment map in 34 and 12 planes correspondingly, i.e., we give vev to $\langle \partial _{12}^M\partial _{34}^K{\widehat{M}}_i \rangle \ne 0$. Physically this corresponds to introducing various codimension two defects into 4*d* theory. For the first $\textrm{SU}(2)_{{\tilde{z}}}$ minimal puncture, we choose to close it without introducing any defect, which in turn corresponds to $K=M=0$ choice in (3.8).

At this value of ${\tilde{z}}$, the superconformal index (3.7) of the four-punctured sphere has a pole. Computing the residue at this pole, we obtain the index of the three-punctured sphere:

3.9 $$\begin{aligned} K_{(3;i,0)}^C(x,y,z)\sim \textrm{Res}_{{\tilde{z}}\rightarrow {\tilde{Z}}_{i;0,0 }^*}K_4^C(x,y,z,{\tilde{z}}) \end{aligned}$$

Here, subscript (3; *i*, 0) refers to the fact that we obtain three-punctured sphere by closing minimal puncture of the four-punctured sphere choosing ${\tilde{a}}_i$ for the vev in (3.8) and not introducing a defect, which corresponds to the choice $K=M=0$ in the same equation.

**Fig. 3:**
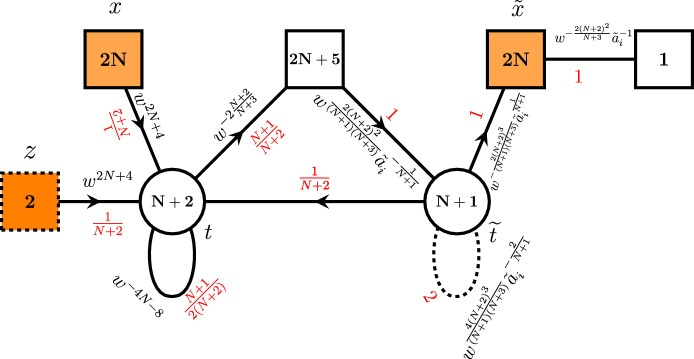
Three-punctured sphere theory with two maximal $\textrm{USp}(2N)$ punctures and one minimal $\textrm{SU}(2)$ puncture obtained after gluing (3.7) and closing one minimal $\textrm{SU}(2)_{{\tilde{z}}}$ puncture without defect. Solid and dashed line attached to the gauge node denotes multiplet in *AS* and ${\overline{AS}}$ representation correspondingly

Calculation of (3.7) and (3.9) results in the theory shown in Fig. 3. Detailed derivations of this section can be found in Appendix C.

Now that we have obtained the desired three-punctured sphere theory we are ready to derive A$\Delta$O. For this purpose, we should close the remaining minimal $\textrm{SU}(2)_z$ puncture by giving non-trivial vev $\langle \partial _{12}^M\partial _{34}^KM \rangle \ne 0$ to a derivative of one of its moment maps. In order to get zero total flux through the resulting two-punctured sphere, we should choose the same moment map as we did closing $\textrm{SU}(2)_{{\tilde{z}}}$ minimal puncture previously. The difference is that now we have to choose non-trivial derivative that is at least one of *K* and *M* numbers is not zero. Physically this corresponds to introducing codimension-two defect into the tube theory with two maximal punctures as shown in Fig. 5b. At the level of the index, we should give the following value to the *z*-fugacity:

3.10 $$\begin{aligned} z=Z_{i;K,M}^*\equiv (pq)^{-\frac{1}{2}}w^{-2\frac{(N+2)^2}{N+3}}{\tilde{a}}_i^{-1} q^{-K}p^{-M}, \end{aligned}$$

Then, according to (3.6), capturing the corresponding pole and gluing the resulting tube with two maximal $\textrm{USp}(2N)$ punctures to an arbitrary theory with at least one puncture of this type as shown in Fig. 5c, we obtain an A$\Delta$O. However, technically it sometimes appears not to be as straightforward. In particular, if we perform these operations with the expression (C.12) instead of A$\Delta$O, we obtain some integral-finite difference operator. It is highly possible that in fact this operator can be written in the form of A$\Delta$O. However, technical issues make it too difficult task and we leave it for future investigation.

**Fig. 4:**
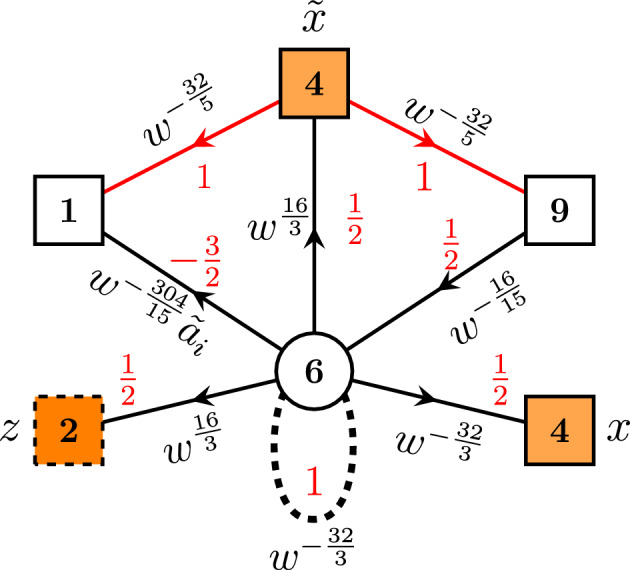
Three-punctured sphere theory with two maximal $\textrm{USp}(4)$ and one minimal $\textrm{SU}(2)$ punctures. Dashed line starting and ending on the gauge $\textrm{SU}(6)$ node as previously corresponds to the matter in the ${\overline{AS}}$ representation

Here, we will instead concentrate on the derivation of next to the lowest rank $C_2$ operator. This case is simpler to analyze. For example, in our trinion theory shown in Fig. 3, when $N=2$, the ${\overline{AS}}$ multiplet of the right $\textrm{SU}(3)$ gauge node becomes just anti-fundamental, and a chain of duality transformations can be used to simplify the theory. These calculations are summarized in Appendix C. As a result, we obtain single-node $\textrm{SU}(6)$ gauge theory shown in Fig. 4 with the corresponding index specified in (C.13).

Finally, we can close $\textrm{SU}(2)_z$ puncture. In particular, we give the following weight consistent with (3.8) to the fugacity *z*:

3.11 $$\begin{aligned} z=Z^*_{i;K,0}=(pq)^{-\frac{1}{2}}w^{-\frac{32}{5}}a_i^{-1}q^{-K}. \end{aligned}$$

**Fig. 5:**
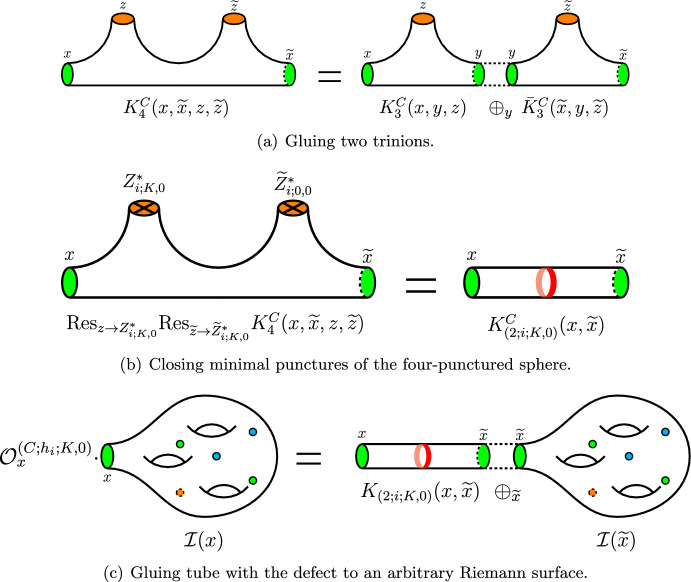
On the figures above we summarize all the steps we go through in order to derive $C_2$ A$\Delta$O given in (3.14). First as shown on the (**a**), we glue two trinions with two maximal $\textrm{USp}(4)$ (shown with *green*) and one minimal $\textrm{SU}(2)$ (shown with *orange*) punctures each. For one of the trinions we conjugate all the charges in order to perform S-gluing. At the level of the superconformal index, this operation is expressed in (3.7). Next, as shown on the (**b**), we close two minimal punctures of the four-punctured sphere. This operation is performed by giving vev $\langle \partial _+^K{\widehat{M}}_i\rangle \ne 0$ to holomorphic derivative of one of the moment map operators with the *U*(1) charge $h_i$. As the result, we obtain tube theory with two maximal $\textrm{USp}(4)$ punctures and codimension-two defect introduced. On the Figure we denote this defect with the red ring. Finally, as the last step of our algorithm shown on the (**c**), we glue this tube with the defect to an arbitrary surface with at least one maximal $\textrm{USp}(4)$ puncture. This results in the action of the set of certain A$\Delta$Os on the index of the original theory (color figure online)

Performing this closure, we obtain the index of the theory for two-punctured sphere with two $\textrm{USp}(4)$ maximal punctures

3.12 $$\begin{aligned} K_{(2;i;K,0)}^C(x,{\tilde{x}})\sim \textrm{Res}_{z\rightarrow Z_{i;K,0 }^*}K_{(3;i,0)}^C(x,{\tilde{x}},z) \end{aligned}$$

The index itself is specified in (C.18). This time it does not have natural gauge theory interpretation in case of general *K* due to the presence of the codimension-two defect.

As a final step of our derivation, we glue the obtained tube with the defect to an arbitrary ${\mathcal {N}}=1$ theory with at least one maximal $\textrm{USp}(4)$ puncture and obtain A$\Delta$O as follows:

3.13 $$\begin{aligned} {{\mathcal {O}}}^{(C_2;h_{k};K,0)}_x\cdot {{\mathcal {I}}}(x)\sim & {} \frac{\left( q;q\right) _\infty ^2\left( p;p\right) _\infty ^2}{2^2\cdot 2!}\oint \frac{d{\tilde{x}}_{1,2}}{2\pi i{\tilde{x}}_{1,2}}\frac{1}{\Gamma _e\left( {\tilde{x}}_{1,2}^{\pm 2}\right) \Gamma _e\left( {\tilde{x}}_1^{\pm 1}{\tilde{x}}_2^{\pm 1}\right) }\nonumber \\{} & {} \times K_{(2;i;K,0)}^C(x,\,{\tilde{x}}) {{\mathcal {I}}}({\tilde{x}}) \end{aligned}$$

Details of this calculation can be found in Appendix C. It leads to the following expression for A$\Delta$O: 
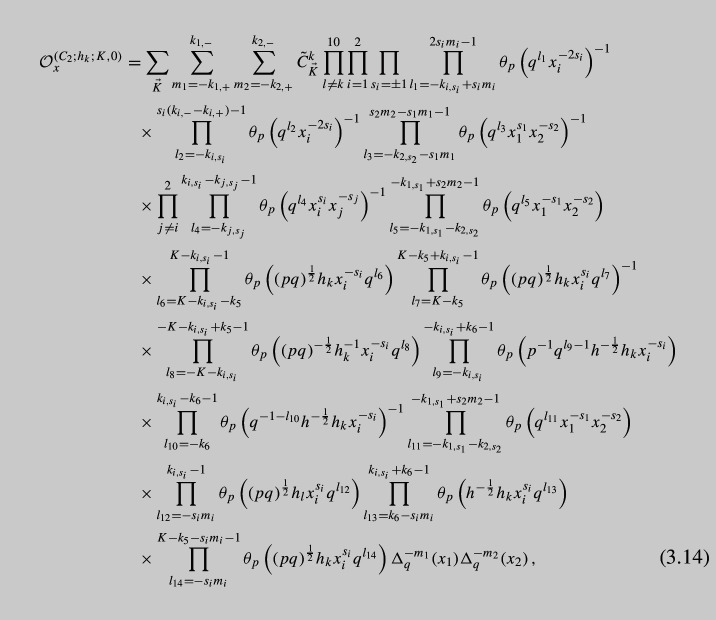


where the constant ${\tilde{C}}^k_{\vec {K}}$ is given by 
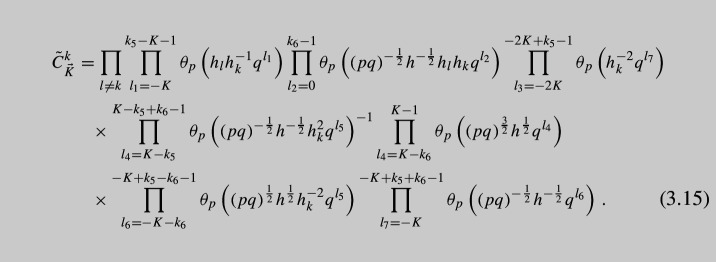


We have also introduced notation $h_i$, $i=1,\dots ,10$ for *U*(1) charges of the moment maps we act on. Notice that according to (3.1) moment maps of both minimal and maximal punctures have the very same charges, so that in fact

3.16 $$\begin{aligned} h_i=w^{\frac{32}{5}}{\tilde{a}}_i,\qquad h\equiv \prod \limits _{j=1}^{10} h_j=w^{64}, \end{aligned}$$

for both charges of the maximal punctures we act on and minimal punctures we close. We have also introduced here an overall *U*(1) charge *h*. The operator itself is labeled by an index *k* according to the choice of *U*(1) charge $h_k$ of the moment map we give vev to in order to close the minimal puncture and to introduce defects into the theory.

The first sum in the expression is performed over all possible partitions $\vec {K}=(k_1,\cdots ,k_6)$ of the integer *K*. Finally, the products in (3.14) should be understood as ordered ones, i.e., one should assume $\prod _{i=n_1}^{n_2}$ is just 1 if $n_2<n_1$ since there are no terms in the product. The coefficient ${\tilde{C}}^k_{\vec {K}}$ is chosen so that the *x*-independent factors of the highest order shift terms $\Delta _q^{\pm K}(y_{1,2})$ are just one.

As expected obtained operators differ from the canonical $BC_n$ vD. In order to obtain the latter one, we should instead consider compactifications of 6*d* rank-Q E-string theory using the very same approach described in details on our paper. One of the most important difference from the $BC_n$ vD model is the number of parameters it depends on. Higher-rank (i.e., rank higher than one) vD model depends on *p*, *q* and another 9 parameters. This number of parameters is independent of the rank of the *BC*-type root system. Meanwhile, our $C_2$ operators depend on *p*, *q* and another 10 parameters. Moreover, in the present paper due to technical difficulties we failed to derive higher-rank $C_N$ generalizations. But from our construction we can expect them to depend on $2N+6$ parameters corresponding to *U*(1) charges of the moment maps.^5^

Notice that the expression above is not the most general since we could have closed $\textrm{SU}(2)_z$ minimal puncture using general *K* and *M* integers in (3.10), but we will concentrate here only on the case of non-trivial *K* while putting $M=0$. As we see, the operator appears to be quite complicated and it is hard to analyze it. Instead it is useful to write down *basic* operator corresponding to the choice of $K=1$, i.e., the simplest non-trivial case. Taking into account six possible partitions $\vec {K}$, we can write down explicit form of A$\Delta$O: 
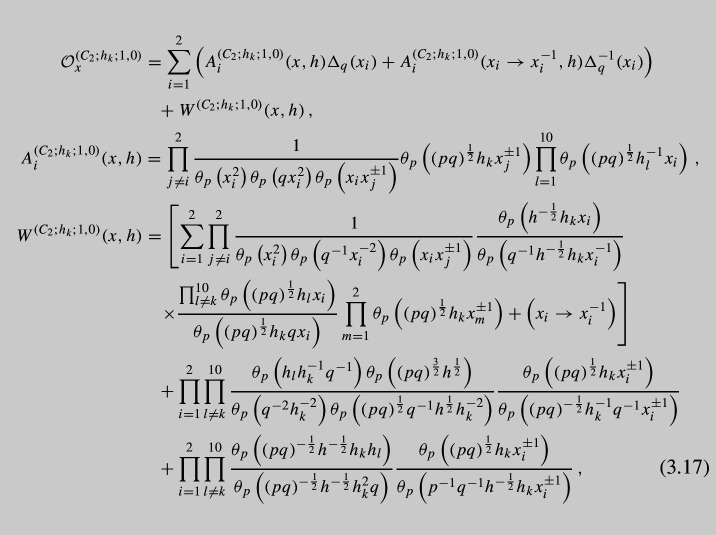


One thing to be noticed is that just as in the case of vD model, the constant term choice is not unique. What really defines this constant term are the following properties. First of all it can be noticed that $W^{(h_k;1,0)}(x,h)$ is an elliptic function in both $x_1$ and $x_2$ with periods 1 and *p*. In the fundamental domain, this elliptic function has poles located at:

3.18 $$\begin{aligned} x_i=sq^{\pm \frac{1}{2}},\qquad x_i=sq^{\pm \frac{1}{2}}p^{\frac{1}{2}}, \quad s=\pm 1, \end{aligned}$$

which are in fact exactly the same as the poles of vD model. Corresponding residues are given by:

3.19 $$\begin{aligned} \textrm{Res}_{x_i=sq^{\frac{1}{2}}}W^{(C_2;h_l;1,0)}(x,h)&=-s\frac{q^{\frac{1}{2}}\prod \limits _{k=1}^{10}\theta _p\left( (pq)^{\frac{1}{2}}sq^{-\frac{1}{2}}h_k\right) }{2\left( p;p\right) _\infty ^2\theta _p\left( q^{-1}\right) }\prod \limits _{j\ne i}^2 \frac{\theta _p\left( (pq)^{\frac{1}{2}}h_lx_j^{\pm 1}\right) }{\theta _p\left( sq^{-\frac{1}{2}}x_j^{\pm 1} \right) }\,, \nonumber \\ \textrm{Res}_{x_i=sq^{-\frac{1}{2}}}W^{(C_2;h_l;1,0)}(x,h)&=s\frac{q^{-\frac{1}{2}}\prod \limits _{k=1}^{10}\theta _p\left( (pq)^{\frac{1}{2}}sq^{-\frac{1}{2}}h_k\right) }{2\left( p;p\right) _\infty ^2\theta _p\left( q^{-1}\right) }\prod \limits _{j\ne i}^2 \frac{\theta _p\left( (pq)^{\frac{1}{2}}h_lx_j^{\pm 1}\right) }{\theta _p\left( sq^{-\frac{1}{2}}x_j^{\pm 1} \right) }\,, \nonumber \\ \textrm{Res}_{x_i=sp^{\frac{1}{2}}q^{\frac{1}{2}}}W^{(C_2;h_l;1,0)}(x,h)&=-s\frac{ph^{-\frac{1}{2}}\prod \limits _{k=1}^{10}\theta _p\left( sh_k\right) }{2\left( p;p\right) _\infty ^2\theta _p\left( q^{-1}\right) }\prod \limits _{j\ne i}^2 \frac{\theta _p\left( (pq)^{\frac{1}{2}}h_lx_j^{\pm 1}\right) }{\theta _p\left( (pq)^{-\frac{1}{2}}sx_j^{\pm 1} \right) }\,, \nonumber \\ \textrm{Res}_{x_i=sp^{\frac{1}{2}}q^{-\frac{1}{2}}}W^{(C_2;h_l;1,0)}(x,h)&=s\frac{pq^{-1}h^{-\frac{1}{2}}\prod \limits _{k=1}^{10}\theta _p\left( sh_k\right) }{2\left( p;p\right) _\infty ^2\theta _p\left( q^{-1}\right) }\prod \limits _{j\ne i}^2 \frac{\theta _p\left( (pq)^{\frac{1}{2}}h_lx_j^{\pm 1}\right) }{\theta _p\left( (pq)^{-\frac{1}{2}}sx_j^{\pm 1} \right) }\,. \end{aligned}$$

This concludes our derivation of the $C_2$ A$\Delta$O. All possible details of these derivations can be found in Appendix C.

## Properties of the $C_2$ operators

There is a number of interesting properties that the operators we derived should possess by construction. In this section, we discuss checks and, where it is possible, give proofs of these properties using explicit expressions we have derived.

The most important but also most complicated property to discuss is the so-called *kernel property* of our operators. The main idea behind it is that the superconformal index of any ${\mathcal {N}}=1$ theory obtained in the compactifications of 6*d* minimal $(D_5,D_5)$ conformal matter theory is a *kernel function* of our operators. The kernel function is defined by the following mathematical identity:

4.1 $$\begin{aligned} {{\mathcal {O}}}_z^{(G_1;h_i;r,m)}\cdot {{\mathcal {I}}}\left[ {{\mathcal {C}}}_{g,s}[z,u]\right] = {{\mathcal {O}}}_u^{(G_2;h_i;r,m)}\cdot {{\mathcal {I}}}\left[ {{\mathcal {C}}}_{g,s}[z,u]\right] . \end{aligned}$$

**Fig. 6:**
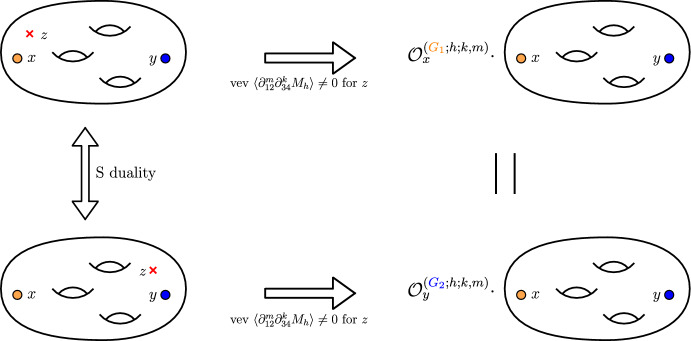
On this figure, we represent the argument in favor of the kernel property of ${\mathcal {N}}=1$ indices. For this, we consider an index of a theory obtained in the compactification of the minimal $(D_5,D_5)$ conformal matter theory on a generic Riemann surface with at least two maximal punctures *(shown as colored disks on the figure)* of any types with fugacities *x* and *y* as well as one minimal $\textrm{SU}(2)$ puncture *(shown as red crosses)* with the fugacity *z*. For example in case of the $A_2C_2$ tube and kernel property (4.2), one maximal puncture has $\textrm{USp}(4)$ global symmetry, while the second one has $\textrm{SU}(3)$ symmetry. Then, we close the minimal puncture by giving space-dependent vev $\langle \partial _{12}^m\partial _{34}^k M \rangle \ne 0$ to one of the moment maps of this puncture. As discussed in the paper, this corresponds to the introduction of the codimension-two defect into 4*d* effective description and results in the action of the A$\Delta$O on one of the maximal punctures of the index of the theory corresponding to the Riemann surface with only two maximal punctures. This operation can be performed in different duality frames. In each frame, we obtain operators acting on one of the two punctures. Since the result of the calculation should not depend on the duality frame we choose, we conclude that the action of two, possibly different operators, on two different punctures leads to two expressions equal to each other. Hence, we arrive to the kernel property Eq. (4.1) (color figure online)

Physically we consider the superconformal index of the theory obtained in the 6*d* compactification on the Riemann surface ${{\mathcal {C}}}_{g,s}[z,u]$ with at least two maximal punctures parametrized by the fugacities *z* and *u* correspondingly. In general, there can be as many punctures as we want. The claim here is that any such superconformal index plays the role of the *kernel function* for the derived A$\Delta$Os according to (4.1). Namely we can act with our operators on different punctures and we should always obtain the same result. This property is expected to hold due to an argument coming from the geometry of compactification shown in Fig. 6. Equivalently, it can be understood from the invariance of the superconformal indices under *S* duality transformations.

In principle, we can choose any sphere with multiple punctures to check the kernel property. Natural simplest candidate would be WZW model for the two-punctured sphere with two maximal $\textrm{USp}(2N)$ punctures for which the index is given in (C.18) with $K=0$. However, we will check a more interesting and trickier case where two punctures are of different types. Namely we take the tube theory shown in Fig. 2a which has one $\textrm{SU}(N+1)$ and one $\textrm{USp}(2N)$ maximal puncture. Then for $N=2$, the kernel property (4.1) in this case reads:

4.2 $$\begin{aligned} {{\mathcal {O}}}^{(A_2;{\tilde{h}}_i^{-1};r,m)}_{y}K_2^{AC}(x,y)= {{\mathcal {O}}}^{(C_2;h_i;r,m)}_{x}K_2^{AC}(x,y), \end{aligned}$$

where $K_2^{AC}(x,y)$ is the index of the tube specified in (C.5) with *x* and *y* being fugacities of $\textrm{USp}(4)$ and $\textrm{SU}(3)$ punctures correspondingly. Operators ${{\mathcal {O}}}^{(A_2;{\tilde{h}}_i;1,0)}_{y}$ and ${{\mathcal {O}}}^{(C_2;h_i;1,0)}_{x}$ are given in (2.13) and (3.17) correspondingly. Notice that in order for the kernel property to work we need to close minimal punctures in the same way on two sides of the equation, i.e., we have to give vev to the same moment map of the minimal puncture. In particular, in $A_2$ case we should give vevs to the minimal puncture moment map of the charge ${\tilde{h}}_i^{-1}{\tilde{h}}^{\frac{1}{4}}$ where ${\tilde{h}}_i$ are given in (2.5). At the same time, in $C_2$ case minimal puncture is closed by giving vev to the moment map of charge $h_i$ specified in (3.16). Using the map (3.4), we can see that the only map that matches between the two is

4.3 $$\begin{aligned} {\tilde{h}}_{10}^{-1}{\tilde{h}}^{\frac{1}{4}}=v^{-24}w^{-8}=w^{-\frac{32}{5}}{\tilde{a}}_{10}^{-1}=h_{10}. \end{aligned}$$

If we would like to check the kernel property for other ways of closing minimal punctures, we should consider $A_2$ operators (2.10) with the non-flipped moment maps so that in this case we close the minimal puncture with the vevs of the moment map with charge ${\tilde{h}}_i{\tilde{h}}^{-\frac{1}{4}}$. Because of this complication, here we consider only closing with the vev given to the moment map of charge (4.3). The proofs for other operators should work identically. Also in our proof we restrict ourselves to only basic operators (3.17) and (2.13), i.e., we fix $r=1,m=0$ (or equivalently $m=1,r=0$ on both sides of the kernel equation (4.2). We have to do it since higher operators specified in (3.14) and (2.10) are too complicated for the analysis. However, the kernel property (4.2) should also work for these higher operators as well.

Since we precisely know explicit expressions for both operators (2.13) and (3.17) as well as supposed kernel function (C.5), it is straightforward to check this kernel identity. Acting with finite difference operators of two kinds on the kernel, we get two algebraic functions. Equality of these functions implies validity of the kernel property (4.2). In Appendix D, we give details of the analytic proof of this identity.

**Fig. 7:**
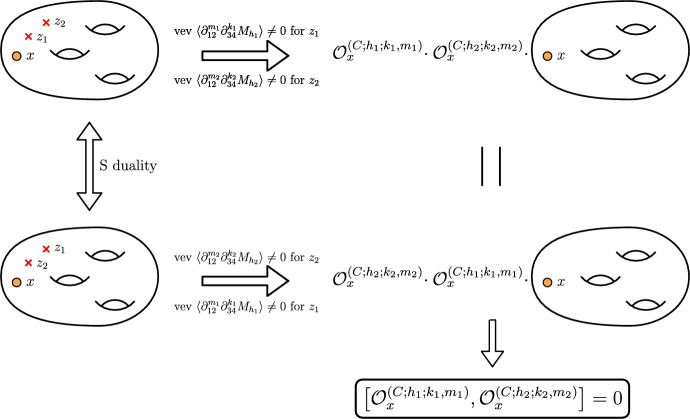
On this figure, we represent the argument in favor of commutation of A$\Delta$Os derived in the present paper. For this, we start with a theory obtained in compactification of 6*d* minimal $\left( D_5,D_5\right)$ conformal matter on an arbitrary Riemann surface. This surface has one maximal $\textrm{USp}(4)$ puncture *(orange disk)* with fugacity *x* and two minimal $\textrm{SU}(2)$ punctures *(red crosses)* with fugacities $z_1$ and $z_2$ correspondingly. Then, we introduce codimension-two defects into this theory giving space-dependent vevs to some of the moment maps $M_{h_1}$ and $M_{h_2}$ correspondingly. Giving this vev is equivalent to closing puncture, and at the level of the index computations leads to an action of two different A$\Delta$Os. We can perform this operation in any duality frame, and the result should not depend on the choice of a particular frame. Hence, the action of operators on the index does not depend on their order. Since the compactification surface, and hence, the effective 4*d* descriptions were chosen arbitrarily, we conclude that the operators themselves also commute (color figure online)

Another important property of the derived A$\Delta$Os is their commutation with each other which also directly follows from the S duality of our compactification construction as shown in Fig. 7. Since it does not depend which duality frame we close the minimal punctures in, it also does not matter in which order two different A$\Delta$Os act on the index. Hence, we conclude that all of operators we derived should commute with each other:

4.4 $$\begin{aligned}{} & {} \left[ {{\mathcal {O}}}_x^{(C_2;h_a;K_1,M_1)},\, {{\mathcal {O}}}_x^{(C_2;h_b;K_2,M_2)}\right] =0,\quad \nonumber \\{} & {} \quad \quad \forall ~ ~a,b=1,\ldots ,10;~ K_{1,2}\ge 0;~M_{1,2}\ge 0; \end{aligned}$$

Here, $h_a$ and $h_b$ are moment maps we give vev to in order to close two minimal punctures and $K_{1,2}, M_{1,2}$ are numbers of holomorphic/anti-holomorphic derivatives. Unfortunately it is very hard to prove or even to check these relations for general operators (3.14) of the full tower. However, we can perform checks of the commutation relations (4.4) for the basic operators, i.e., for the case when $K_{1,2}$ and $M_{1,2}$ are taking values 0 and 1. In particular, we show that:

4.5 $$\begin{aligned} \left[ {{\mathcal {O}}}_x^{(C_2;h_a;1,0)},\, {{\mathcal {O}}}_x^{(C_2;h_b;1,0)}\right]= & {} \left[ {{\mathcal {O}}}_x^{(C_2;h_a;0,1)},\, {{\mathcal {O}}}_x^{(C_2;h_b;0,1)}\right] \nonumber \\= & {} \left[ {{\mathcal {O}}}_x^{(C_2;h_a;1,0)},\, {{\mathcal {O}}}_x^{(C_2;h_b;0,1)}\right] \nonumber \\= & {} 0,\qquad \forall ~ a,b =1,\dots ,10. \end{aligned}$$

In Appendix E, we check these relations. In particular, using ellipticity properties of $\theta _p\left( x\right)$ function we prove that the action of the third type of the commutators on an arbitrary trial function is zero. Hence, these commutators are zero themselves. For the first and second type of commutators, it is hard to prove analytically that their action on a trial function is zero. Instead we check these identities perturbatively in *p* and *q* expansion. These checks performed up to a sufficiently high order suggest that both of the commutators are indeed zero.

## Discussion and outlook

In this paper, we have considered 4*d* description of the compactification of the minimal $(D_5,D_5)$ conformal matter theory on a Riemann surface with $\textrm{USp}(4)$ maximal punctures. Using this 4*d* description and introducing codimension-two defect in corresponding theory, we derive $C_2$ generalization of the van Diejen model. In particular, we obtain an infinite set of analytic difference operators acting on the maximal puncture of $C_2$ type. Different operators of our set correspond to different ways of closing minimal punctures on the compactification surfaces. They are organized in a decuplet of basic operators and an infinite tower of operators on top of each of the basic ones.

The operators we obtain are supposed to satisfy interesting and important properties following directly from the geometry of compactifications. First such property is the commutation of all operators. In our paper, using combination of residue computations and perturbative expansions we show that at least all basic operators indeed commute with each other. Second important property is that the superconformal index of any compactification of the minimal $(D_5,D_5)$ conformal matter theory on a surface with several $\textrm{USp}(4)$ punctures is the kernel function of our difference operators. As an example of such kernel function, we considered a tube theory corresponding to the compactification on a sphere with one $\textrm{USp}(4)$ and one $\textrm{SU}(3)$ puncture. In order to prove the kernel property, we should act on two punctures with two different operators: $A_2$ operator derived in [19] from one side and $C_2$ operator derived in the present paper from the other. This fact makes corresponding kernel function very interesting for the study. In the paper, we managed to prove this kernel property fully analytically for the basic $A_2$ and $C_2$ operators.

Another result of our paper is derivation of the full tower of $A_N$ generalization of van Diejen operator originating from the compactification of the minimal $(D_{N+3},D_{N+3})$ conformal matter theory on a Riemann surface with $\textrm{SU}(N+1)$ maximal punctures. We have already derived basic operators of this kind in our previous paper [19]. However, the tower of operators was missing and we filled this gap in the present paper.

The present paper is another brick in our program of establishing dictionary between compactifications of 6*d* SCFTs and integrable analytic difference operators and studying this dictionary in details. There are plenty of ways this research can be continued in. In particular, there should be separate difference operator for each possible 5*d* compactification of 6*d* theory, or equivalently for each possible puncture type from 4*d* prospective. So far several results have been observed in this frame. First of all $BC_1$ van Diejen model was observed in E-string compactifications in several ways [11, 19]. Also previously unknown $A_2$ and $A_3$ operators were derived using compactifications of the minimal conformal matter theories of types $\textrm{SU}(3)$ and $\textrm{SO}(8)$ [10]. Finally, we have considered compactifications of the minimal $(D_{N+3},D_{N+3})$ conformal matter theories on a Riemann surfaces with $A_N$- and $C_2$ (in case of $(D_5,D_5)$ 6*d* theory)-type punctures. However, there are plenty of examples of 6*d* SCFT compactifications that are known up to date [25–34]. Our main goal is to extend our results to more examples of compactifications and ideally establish a dictionary between them and integrable models. Such dictionary can from one point of view shed light on physics of 6*d*, 5*d* and 4*d* supersymmetric gauge theories. From the other point of view, this research program can lead to important results in the field of integrable systems.

First of all as a continuation of the present paper, it is natural to consider compactifications of the minimal $(D_{N+3},D_{N+3})$ conformal matter on Riemann surfaces with other types of punctures. In first place, these are of course punctures of $C_N$ type for general *N*. In the present work, we failed to derive explicit form of the corresponding A$\Delta$O because of the technical difficulties but it is worth trying to overcome them. Second candidate for the study is $\left( A_1\right) ^N$ puncture. Corresponding 4*d* description was derived in [20]. It would be interesting to derive explicit form of the corresponding difference operators and study their properties. For example, we immediately know examples of the kernel functions of these operators even before deriving them explicitly.

Second candidate for the studies are non-minimal $(D_{N+3},D_{N+3})$ conformal matter theories, which are obtained as worldvolume theories of the stack of *k* M5 branes probing $D_{N+3}$ singularity [26]. In this case, the known 5*d* gauge theory, and hence the puncture type on Riemann surface, is a direct generalization of $\left( A_1 \right) ^N$ case of minimal conformal matter compactification discussed above. It corresponds to the $\left( A_{k-1}\right) ^2\times \left( A_{2k-1}\right) ^N\times \left( A_{k-1}\right) ^2$ gauge theory in 5*d* and hence the puncture type with the same global symmetry. Trinion theories with two maximal and one minimal punctures, which are building blocks in the construction of A$\Delta$Os, are also known [35]. So construction of the corresponding difference operators should be straightforward though can happen to be technically complicated.

Finally, one more thing to be made in this direction is the study of the rank-Q E-string theory compactifications along the same lines of research. Unfortunately so far corresponding trinion theories were not obtained but the tube theories were already derived in [36, 37]. Although we cannot derive corresponding A$\Delta$O using only tube theories, in this case we can naturally conjecture it to be $BC_n$ van Diejen model. We can at least test this conjecture by checking corresponding kernel property.

Another possible direction of the research is related to the study of the properties of the operators we derived. Most interesting questions here are related to the eigenfunctions of these operators. Deriving full eigenfunctions is of course difficult and most probably impossible. Realistically we can try to derive certain limits of these eigenfunction. In case we succeed these eigenfunctions can play the same role for indices of ${\mathcal {N}}=1$ theories as Macdonald and Schur polynomials played for the indices of ${\mathcal {N}}=2$ theories [16–18].

It would also be interesting to establish connections of our research program with other integrable models emerging in supersymmetric gauge theories. For example, integrable systems derived using compactifications of the worldvolume theory of *k* M5 branes probing $A_{N-1}$ singularity were shown to be related to the set of transfer matrices [9, 38–40]. It would be interesting to understand if similar connection emerges in case of $D_{N+3}$ singularity.

Also, a relation of E-string theory to the van Diejen model was observed in [41] using quantization of the corresponding Seiberg–Witten curve. Later, authors of this paper generalized their result to 6*d* (1, 0) SCFTs with $\textrm{SO}(N)$ gauge group and $(N-8)$ fundamental flavors obtaining yet another set of difference operators [42]. It would be interesting to clarify precise relation of our construction with the methods and results of these papers.

## Figures and Tables

**Fig. 8:**
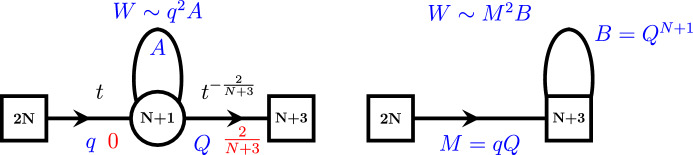
Spiridonov–Warnaar–Vartanov (SWV) duality. On the picture, we also specify superpotentials, UV on the left and dynamically generated on the right sides of dualities. The solid line starting and ending on the same node denotes multiplet in rank-two antisymmetric representation (AS)

**Fig. 9:**
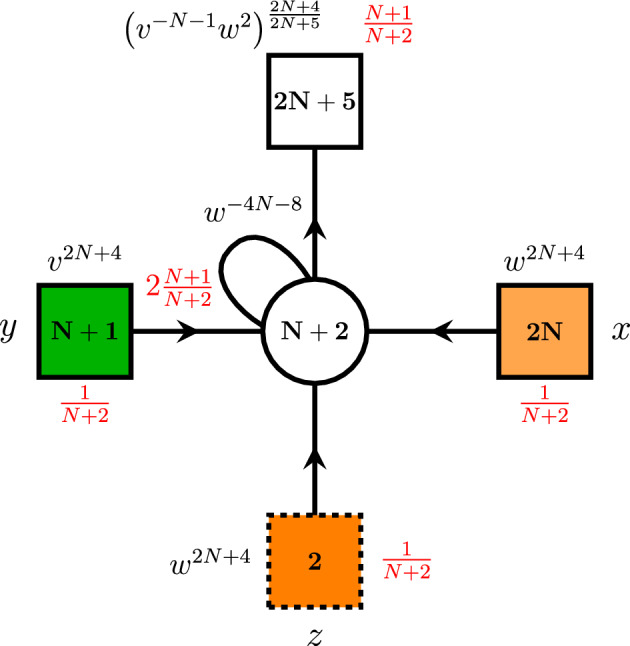
Trinion theory with $\textrm{USp}(2N)_x$ maximal puncture *(light orange)*, $\textrm{SU}(N+1)_y$ maximal puncture *(green)* and $\textrm{SU}(2)_z$ minimal puncture *(orange)*. *U*(1) global symmetries are parametrized using the parametrization (3.3) more natural for the $\textrm{SU}(N+1)$-type punctures. The index of the theory is given in (C.8) (color figure online)

**Fig. 10:**
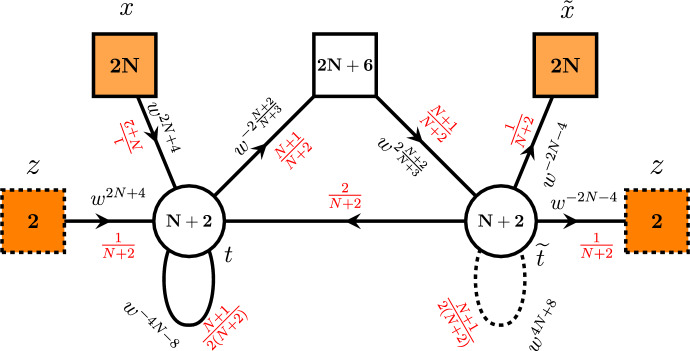
Four-punctured sphere theory with two maximal $\textrm{USp}(2N)$ and two minimal $\textrm{SU}(2)$ punctures. The four-punctured sphere is derived by gluing two trinion theories shown in Fig. 9 and performing some duality transformations specified in the text

**Fig. 11:**
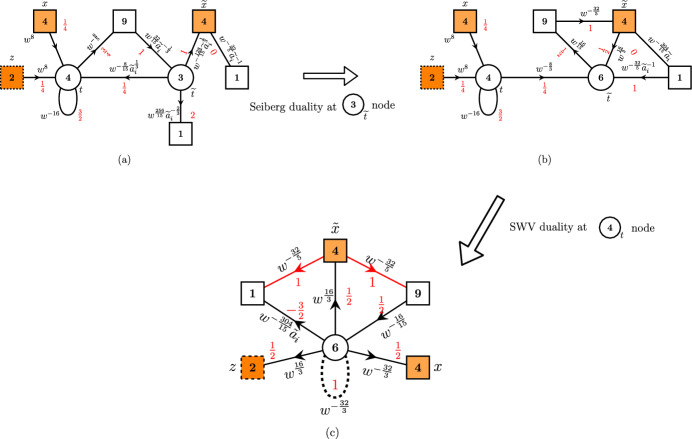
Chain of duality transformations of three-punctured sphere theory in $C_2$ case. We start with the theory derived by gluing two three-punctured sphere theories ${{\mathcal {T}}}^{AC}$ along *A*-type puncture, integrating out $A_2$ node and closing $\textrm{SU}(2)_{{\tilde{z}}}$ minimal puncture. This theory is shown in Figure (**a**) above, and corresponding index is given by (C.13). Then, performing Seiberg duality on the right node of this theory we obtain the quiver shown in Figure (**b**). Finally, we notice that right $\textrm{SU}(4)$ gauge node of theory (**b**) is confining due to SWV duality. Integrating it out we obtain theory shown in the Figure (**c**)

## Data Availability

Data sharing is not applicable to this article as no datasets were generated or analyzed during the current study.

## Special functions

Here, we summarize some definitions and properties of special functions used in the paper.

Elliptic gamma function is defined through the following infinite product:

A.1 $$\begin{aligned} \Gamma _e\left( z\right) \equiv \prod \limits _{k,m=0}^\infty \frac{1-p^{k+1}q^{m+1}/z}{1-p^kq^m z}. \end{aligned}$$

It can be easily seen that the poles of this function are located at the following values of the argument:

A.2 $$\begin{aligned} z=p^{-k}q^{-m},\qquad k,m\in {\mathbb {Z}}_{\ge 0}. \end{aligned}$$

The following relation will be useful in our calculations:

A.3 $$\begin{aligned} \Gamma _e\left( \frac{pq}{z}\right) \Gamma _e\left( z\right) =1. \end{aligned}$$

Also we will often deal with the elliptic beta integral formula

A.4 $$\begin{aligned} \kappa \oint \frac{dz}{4\pi i z}\frac{1}{\Gamma _e\left( z^{\pm 2}\right) }\prod \limits _{j=1}^6\Gamma _e\left( t_iz^{\pm 1}\right) =\prod \limits _{i<j}\Gamma _e\left( t_it_j\right) . \end{aligned}$$

Here, $\kappa$ is defined to be

A.5 $$\begin{aligned} \kappa =(q;q)_{\infty }(p;p)_{\infty }=\prod _{\ell =0}^\infty (1-q^{1+\ell })(1-p^{1+\ell }). \end{aligned}$$

$A_N$ generalization of this formula is

A.6 $$\begin{aligned}{} & {} \frac{\kappa ^N}{N!}\oint \prod \limits _{i=1}^N\frac{dz_i}{2\pi i z_i}\prod \limits _{i\ne j}^{N+1}\Gamma _e\left( \frac{z_i}{z_j}\right) ^{-1}\prod \limits _{i=1}^{N+2}\prod \limits _{j=1}^{N+1} \Gamma _e\left( s_iz_j\right) \Gamma _e\left( t_iz_j^{-1}\right) \nonumber \\{} & {} \quad =\prod \limits _{i=1}^{N+2}\Gamma _e\left( Ss_i^{-1}\right) \Gamma _e\left( Tt_i^{-1}\right) \prod \limits _{i,j=1}^{N+2}\Gamma _e\left( s_it_j\right) ,\qquad \quad \left( T=\prod \limits _{i=1}^{N+2}t_i,~S=\prod \limits _{i=1}^{N+2}s_i\right) .\nonumber \\ \end{aligned}$$

The theta function is defined as follows:

A.7 $$\begin{aligned} \theta _p\left( x\right) \equiv \left( x;p\right) _\infty \left( x^{-1}p;p\right) _\infty , \end{aligned}$$

where $\left( z;p\right) _\infty$ is the usual q-Pochhammer symbol defined as follows:

A.8 $$\begin{aligned} \left( x;p\right) _\infty =\prod \limits _{k=0}^\infty \left( 1-xp^k \right) . \end{aligned}$$

Following properties of theta function will be useful to us

A.9 $$\begin{aligned} \theta _p\left( x\right)= & {} \frac{\Gamma _e\left( qx\right) }{\Gamma _e\left( x\right) },\quad \theta _p\left( x^{-1}\right) =-x^{-1}\theta _p\left( x\right) ,\quad \nonumber \\ \theta _p\left( xp^m\right)= & {} (-1)^mx^{-m}p^{-\frac{1}{2}m(m-1)}\theta _p\left( x\right) , \nonumber \\ \frac{ \Gamma _e\left( p^L q^K x \right) }{ \Gamma _e\left( x\right) }= & {} \prod _{j=0}^{K-1} \theta _p\left( q^j x\right) \prod _{j=0}^{L-1} \theta _q\left( q^K p^j x\right) \end{aligned}$$

We will also use the following duality identity from [43]:

A.10 $$\begin{aligned} V(\underline{t})=\prod \limits _{1\le j<k\le 4}^8\Gamma _e\left( t_jt_k\right) \Gamma _e\left( t_{j+4}t_{k+4}\right) V(\underline{s}), \end{aligned}$$

where

A.11 $$\begin{aligned} V(\underline{t})\equiv & {} \kappa \oint \frac{dz}{2\pi iz}\frac{\prod \limits _{j=1}^8\Gamma _e\left( t_jz^{\pm 1}\right) }{\Gamma _e\left( z^{\pm 2} \right) }, \qquad \prod \limits _{j=1}^8t_i=pq,\quad |t_j|,|s_j|<1. \nonumber \\ s_j= & {} \rho ^{-1}t_j,\quad j=1,2,3,4;\quad s_j=\rho t_j,\quad \nonumber \\ j= & {} 5,6,7,8;\quad \rho \equiv \sqrt{\frac{t_1t_2t_3t_4}{pq}} \end{aligned}$$

## Derivation of higher tower $A_N$ operators

In this appendix, we will give details of the derivation of $A_N$ A$\Delta$Os that we summarize in Sect. 2. We start with the four-punctured sphere theory whose quiver is shown in Fig. 1. This theory and its superconformal index were derived in [19]. The index is given by the following expression:

B.1 $$\begin{aligned}{} & {} K_4(x,{\tilde{x}},z,{\tilde{z}})=\kappa _{N+2}\oint \prod \limits _{i=1}^{N+2}\frac{dt_i}{2\pi i t_i} \prod \limits _{i\ne j}^{N+3}\frac{1}{\Gamma _e\left( \frac{t_i}{t_j}\right) } \nonumber \\{} & {} \quad \times \prod \limits _{i=1}^{N+3}\prod \limits _{j=1}^{N+1}\prod \limits _{k=1}^{2N+4} \Gamma _e\left( (pq)^{\frac{1}{N+3}}\left( u^{-1}w \right) ^{4\frac{N+2}{N+3}}x_j^{-1}t_i\right) \Gamma _e\left( (pq)^{\frac{1}{N+3}}\left( uw^{-1} \right) ^{2\frac{(N+2)(N+1)}{N+3}}z^{\pm 1}t_i\right) \nonumber \\{} & {} \quad \times \Gamma _e\left( (pq)^{\frac{N+1}{2(N+3)}}u^{-\frac{(N+1)^2}{N+3}}v^{N+1}w^{-2\frac{N+1}{N+3}}a_k^{-1}t_i^{-1}\right) \Gamma _e\left( (pq)^{\frac{1}{2}}u^{N+3}v^{-N-1}w^{-2}a_kx_j\right) \nonumber \\{} & {} \quad \times \Gamma _e\left( (pq)^{\frac{N+1}{2(N+3)}}u^{-2\frac{(N+1)(N+2)}{N+3}}v^{-2(N+1)(N+2)}w^{-4\frac{N+2}{N+3}}t_i^{-1}\right) \nonumber \\{} & {} \quad \times \Gamma _e\left( (pq)^{\frac{1}{2}}u^{2N+4}v^{2(N+1)(N+2)}x_j\right) \Gamma _e\left( (pq)^{\frac{1}{2}}\left( uv w^{-2}\right) ^{-N-1}a_lz^{\pm 1} \right) \nonumber \\{} & {} \quad \times \Gamma _e\left( (pq)^{\frac{1}{2}}v^{2(N+1)(N+2)}w^{2N+4}z^{\pm 1}\right) \Gamma _e\left( (pq)^{\frac{N+1}{2(N+3)}}u^{2\frac{(N+1)(N+2)^2}{N+3}}w^{4\frac{(N+2)^2}{N+3}}t_i^{-1}\right) \nonumber \\{} & {} \quad \times \Gamma _e\left( (pq)^{\frac{1}{2}}u^{-2N(N+2)}w^{-4N-8}{\tilde{x}}_j\right) \Gamma _e\left( (pq)^{\frac{1}{2}}u^{-2(N+1)(N+2)}w^{-2N-4}{\tilde{z}}^{\pm 1}\right) \nonumber \\{} & {} \quad \times \Gamma _e\left( (pq)^{\frac{1}{N+3}} \left( u^{-1}w \right) ^{4\frac{N+2}{N+3}}t_i{\tilde{x}}_j^{-1}\right) \Gamma _e\left( (pq)^{\frac{1}{N+3}}\left( uw^{-1} \right) ^{2\frac{(N+1)(N+2)}{N+3}}t_i{\tilde{z}}^{\pm 1}\right) . \end{aligned}$$

This is an index of an $\textrm{SU}(N+3)$ SQCD with $2N+6$ flavors and a superpotential. Variables $x,{\tilde{x}},z,{\tilde{z}}$ are fugacities of the global symmetry of two maximal $\textrm{SU}(N+1)_{x,{\tilde{x}}}$ and two minimal $\textrm{SU}(2)_{z,{\tilde{z}}}$ punctures correspondingly.

We close the two $\textrm{SU}(2)$ punctures by setting *z* and ${\tilde{z}}$ to the values in (2.7). Performing calculations for general values of the charges is complicated. So we will perform here the calculation only for one of the charges fixing ${\tilde{h}}_i={\tilde{h}}_{2N+6}=\left( u^Nw^2\right) ^{-2N-4}$. Corresponding positions of the poles are then given by

B.2 $$\begin{aligned} z= & {} (pq)^{-\frac{1}{2}} \left( w u^{N+1} \right) ^{-2N-4} q^{-M} p^{-L}, \;\;\;\;\;\;\; \nonumber \\ {\tilde{z}}= & {} (pq)^{-\frac{1}{2}} \left( w u^{N+1} \right) ^{2N+4} q^{-{\tilde{M}}} p^{-{\tilde{L}}}. \end{aligned}$$

Also as discussed in Sect. 2, we will further consider ${\tilde{L}}={\tilde{M}}=0$.

The pole in ${\tilde{z}}$ is an explicit simple pole so we can just take the residue. The pole in *z*, however, is due to contour pinching. To see this clearly, it is useful to first perform a Seiberg duality leading to an index of $SU(N+2)$ gauge theory with $2N+5$ flavors, with the following index,

B.3 $$\begin{aligned}{} & {} K_{(2;2N+6;L,M)}^{A_N}\left( x,{\tilde{x}}\right) =\kappa _{N+1}\oint \prod \limits _{i=1}^{N+1}\frac{dt_i}{2\pi it_i} \prod \limits _{i\ne j}^{N+2}\frac{1}{\Gamma _e\left( \frac{t_i}{t_j}\right) } \nonumber \\{} & {} \quad \times \prod \limits _{i=1}^{N+2}\prod \limits _{j=1}^{N+1}\prod \limits _{k=1}^{2N+4}\Gamma _e\left( (pq)^{\frac{1}{2(N+2)}}u^{2N+4}x_jt_i\right) \Gamma _e\left( (pq)^{\frac{1}{2}}u^{-N-3}v^{N+1}w^2a_k^{-1}{\tilde{x}}_j^{-1}\right) \nonumber \\{} & {} \quad \times \Gamma _e\left( (pq)^{\frac{1}{2}}\left( uv^{N+1}\right) ^{-2N-4}{\tilde{x}}^{- 1}\right) \Gamma _e\left( (pq)^{\frac{1}{2(N+2)}}u^{2N+4}{\tilde{x}}_jt_i\right) \nonumber \\{} & {} \quad \times \Gamma _e\left( (pq)^{\frac{N+3}{2N+4}} u^{(2N+4)(N+1)} w^{4N+8} t_i q^M p^L\right) \Gamma _e\left( (pq)^{-\frac{N+1}{2N+4}} u^{-(2N+4)(N+1)} t_i q^{-M} p^{-L} \right) \nonumber \\{} & {} \quad \times \Gamma _e\left( (pq)^{\frac{N+3}{2(N+2)}}u^{-2(N+1)(N+2)}t_i\right) \Gamma _e\left( (pq)^{\frac{N+1}{2(N+2)}}\left( uv\right) ^{-N-1}w^{-2}a_kt_i^{-1}\right) \nonumber \\{} & {} \quad \times \Gamma _e\left( (pq)^{\frac{N+1}{2(N+2)}}v^{2(N+1)(N+2)}t_i^{-1}\right) \Gamma _e\left( (pq)^{\frac{1}{2}}\left( u^Nw^2 \right) ^{-2N-4}{\tilde{x}}_j\right) \end{aligned}$$

where we have already dropped irrelevant overall factors and substituted values of *z* from (B.2). The subscript of the index as usually contains all the information of the underlying theory. In particular, 2 stands for the number of punctures. Index $2N+6$ stands for the index of the moment map we give vev to in order to close the puncture (moment map of charge ${\tilde{h}}_{2N+6}{\tilde{h}}^{-\frac{1}{4}}$ in this case). Finally, (*L*, *M*) denotes derivative powers in the vev and is the same as *L* and *M* integers in (B.2). As can be seen from the expression above, the contour pinching is due to collision of poles at the following values:

B.4 $$\begin{aligned}{} & {} t_i=(pq)^{-\frac{1}{2(N+2)}}u^{-2(N+2)}x_i^{-1}q^{-m_i}p^{-l_i},\quad \nonumber \\{} & {} t_{N+2}=(pq)^{\frac{N+1}{2(N+2)}}u^{2(N+1)(N+2)}q^{M-m_{N+2}}p^{L-l_{N+2}} , \nonumber \\{} & {} \textrm{or} \nonumber \\{} & {} t_i=(pq)^{-\frac{1}{2(N+2)}}u^{-2(N+2)}{\tilde{x}}_i^{-1}q^{-m_i}p^{-l_i},\quad \nonumber \\{} & {} t_{N+2}=(pq)^{\frac{N+1}{2(N+2)}}u^{2(N+1)(N+2)}q^{M-m_{N+2}}p^{L-l_{N+2}}, \nonumber \\ \end{aligned}$$

where $m_i$ and $l_i$ are partitions of *M* and *L*, respectively. There are $(N+2)!$ such poles coming from permutations of $t_i$ but they all give the same result and we are ignoring overall factors. It can be checked that the two lines in (B.4) give the same result so we will consider only the first set of poles. After computing the residue, we get the following expression for particular partitions $\vec {l},\vec {m}$,

B.5 $$\begin{aligned}{} & {} K_{\left( 2;2N+6;\vec {m},\vec {l}\right) }^{A_N}(x,{\tilde{x}})=\Gamma _e\left( pq\right) \prod _{j=1}^{N+2} \frac{ \prod _{n=1}^{M-m_{N+2}} \theta _p\left( q^{-n} \right) \prod _{n=1}^{L-l_{N+2} } \theta _q\left( q^{m_{N+2}-M} p^{-n} \right) }{ \prod _{n=1}^{m_j} \theta _p\left( q^{-n} p^{-l_j} \right) \prod _{n=1}^{l_j } \theta _q\left( p^{-n} \right) } \nonumber \\{} & {} \quad \prod \limits _{i=1}^{N+1} \prod \limits _{k=1}^{2N+4}\prod \limits _{j\ne i}^{N+1} \Gamma _e\left( \frac{{\tilde{x}}_i}{x_i}q^{-m_i} p^{-l_i} \right) \frac{\Gamma _e\left( (pq)^{\frac{1}{2}}u^{2(N+2)^2}q^{M-m_{N+2}}p^{L-l_{N+2}} x_i\right) }{\Gamma _e\left( (pq)^{\frac{1}{2}}u^{2(N+2)^2}q^{M+m_i-m_{N+2}}p^{L+l_i-l_{N+2}}x_i\right) } \nonumber \\{} & {} \qquad \times \frac{\Gamma _e\left( (pq)^{-\frac{1}{2}}u^{-2(N+2)^2}x_i^{-1}q^{-M-m_i} p^{-L-l_i} \right) }{\Gamma _e\left( (pq)^{-\frac{1}{2}}u^{-2(N+2)^2}x_i^{-1}q^{m_{N+2}-M-m_i} p^{l_{N+2}-L-l_i} \right) } \Gamma _e\left( \frac{{\tilde{x}}_j}{x_i}q^{-m_i} p^{-l_i} \right) \nonumber \\{} & {} \qquad \times \Gamma _e\left( (pq)^{\frac{1}{2}}u^{N+3}v^{-N-1}w^{-2}a_kx_iq^{m_i} p^{l_i} \right) \Gamma _e\left( (pq)^{\frac{1}{2}}\left( u^Nw^2\right) ^{2N+4}x_i^{-1}q^{M-m_i} p^{L-l_i} \right) \nonumber \\{} & {} \qquad \times \Gamma _e\left( (pq)^{\frac{1}{2}}u^{-2(N+2)^2}x_i^{-1}q^{-m_i} p^{-l_i} \right) \Gamma _e\left( (pq)^{\frac{1}{2}}\left( uv^{N+1}\right) ^{2N+4}x_iq^{m_i} p^{l_i} \right) \nonumber \\{} & {} \qquad \times \Gamma _e\left( (pq)^{\frac{1}{2}}u^{-N-3}v^{N+1}w^2a_k^{-1}{\tilde{x}}_i^{-1}\right) \Gamma _e\left( q^{m_{N+2}-M}p^{l_{N+2}-L} \left( vu^{-1}\right) ^{2(N+1)(N+2)}\right) \nonumber \\{} & {} \qquad \times \frac{\Gamma _e\left( \frac{x_j}{x_i}q^{-m_i} p^{-l_i} \right) }{\Gamma _e\left( \frac{x_j}{x_i}q^{m_j-m_i} p^{l_j-l_i} \right) } \Gamma _e\left( (pq)^{\frac{1}{2}}u^{2(N+2)^2}q^{M-m_{N+2}} p^{L-l_{N+2}} {\tilde{x}}_i\right) \nonumber \\{} & {} \qquad \times \Gamma _e\left( (pq)^{\frac{1}{2}}\left( u^Nw^2\right) ^{-2N-4}{\tilde{x}}_i\right) \Gamma _e\left( pq \left( u^{N+1}w\right) ^{4N+8} q^{2M-m_{N+2}} p^{2L-l_{N+2}} \right) \nonumber \\{} & {} \qquad \times \Gamma _e\left( (pq)^{\frac{1}{2}}\left( uv^{N+1}\right) ^{-2N-4}{\tilde{x}}_i^{-1}\right) \Gamma _e\left( \left( u^{2N+5}v\right) ^{-N-1}w^{-2}a_kq^{m_{N+2}-M} p^{l_{N+2}-L} \right) .\nonumber \\ \end{aligned}$$

To get the $A\Delta O$ acting on $\textrm{SU}(N+1)_x$ puncture, we should S glue this tube to the index of an arbitrary theory ${{\mathcal {I}}}({\tilde{x}})$ with $SU(N+1)_{{\tilde{x}}}$ puncture and sum over all partitions,

B.6 $$\begin{aligned} {{\mathcal {I}}}_1(x)=\kappa _{N}\sum _{\{m_i\},\{l_i\}}\oint \prod \limits _{i=1}^{N}\frac{d{\tilde{x}}_i}{2\pi i {\tilde{x}}_i}\prod \limits _{i\ne j}^{N+1}\frac{1}{\Gamma _e\left( \frac{{\tilde{x}}_i}{{\tilde{x}}_j}\right) } K_{\left( 2;2N+6;\vec {m},\vec {l}\right) }^{A_N}({\tilde{x}},x) {{\mathcal {I}}}_0({\tilde{x}}),\nonumber \\ \end{aligned}$$

Note that there is a zero in (B.5) coming from $\Gamma _e\left( pq\right)$ but this is canceled against the pinching of ${\tilde{x}}$ at the following values,

B.7 $$\begin{aligned} {\tilde{x}}_i=x_{i}q^{m_i-s_i}p^{l_i-r_i} ,\quad \sum \limits _{i=1}^{N+1}s_i=M-m_{N+2},\quad \sum \limits _{i=1}^{N+1}r_i=L-l_{N+2}, \end{aligned}$$

up to permutations of $x_i$ which give the same result due to the Weyl symmetry of $A_N$ root system. If we specify ${\tilde{x}}_i$ to the values written above, we obtain double pole due to the contour pinching of (B.6) but as we mentioned one of them is canceled by the zero of $\Gamma _e\left( pq\right)$. Computing the residue of the remaining pole, we obtain the following contribution of each individual partition:

B.8 $$\begin{aligned}{} & {} {{\mathcal {I}}}^{\varvec{m,l,s,r}}_1(x)= \prod _{i=1}^{N+1} \prod _{n=1}^{N+2}\prod \limits _{k=1}^{2N+4}\prod \limits _{j\ne i}^{N+1} \frac{\Gamma _e\left( (pq)^{\frac{1}{2}}u^{2(N+2)^2}q^{M-m_{N+2}}p^{L-l_{N+2}} x_i\right) }{\Gamma _e\left( (pq)^{\frac{1}{2}}u^{2(N+2)^2}q^{M+m_i-m_{N+2}}p^{L+l_i-l_{N+2}}x_i\right) } \nonumber \\{} & {} \quad \times \frac{ \prod _{n=1}^{M-m_{N+2}} \theta _p\left( q^{-n} \right) \prod _{n=1}^{L-l_{N+2} } \theta _q\left( q^{m_{N+2}-M} p^{-n} \right) }{ \prod _{n=1}^{m_j} \theta _p\left( q^{-n} p^{-l_j} \right) \prod _{n=1}^{l_j } \theta _q\left( p^{-n} \right) \prod _{n=1}^{s_i} \theta _p\left( q^{-n} p^{-r_i} \right) \prod _{n=1}^{r_i } \theta _q\left( p^{-n} \right) } \nonumber \\{} & {} \quad \times \frac{\Gamma _e\left( (pq)^{-\frac{1}{2}}u^{-2(N+2)^2}x_i^{-1}q^{-M-m_i} p^{-L-l_i} \right) }{\Gamma _e\left( (pq)^{-\frac{1}{2}}u^{-2(N+2)^2}x_i^{-1}q^{m_{N+2}-M-m_i} p^{l_{N+2}-L-l_i} \right) } \Gamma _e\left( \frac{x_j}{x_i}q^{m_j-s_j-m_i} p^{l_j-r_j-l_i} \right) \nonumber \\{} & {} \quad \times \Gamma _e\left( (pq)^{\frac{1}{2}}u^{N+3}v^{-N-1}w^{-2}a_kx_iq^{m_i} p^{l_i} \right) \Gamma _e\left( (pq)^{\frac{1}{2}}\left( u^Nw^2\right) ^{2N+4}x_i^{-1}q^{M-m_i} p^{L-l_i} \right) \nonumber \\{} & {} \quad \times \Gamma _e\left( (pq)^{\frac{1}{2}}u^{-2(N+2)^2}x_i^{-1}q^{-m_i} p^{-l_i} \right) \Gamma _e\left( (pq)^{\frac{1}{2}}u^{-N-3}v^{N+1}w^2a_k^{-1}x_i^{-1} q^{s_i-m_i}p^{r_i-l_i} \right) \nonumber \\{} & {} \quad \times \Gamma _e\left( (pq)^{\frac{1}{2}}\left( uv^{N+1}\right) ^{-2N-4}x_i^{-1} q^{s_i-m_i}p^{r_i-l_i}\right) \frac{\Gamma _e\left( \frac{x_j}{x_i}q^{-m_i} p^{-l_i} \right) }{\Gamma _e\left( \frac{x_j}{x_i}q^{m_j-m_i} p^{l_j-l_i} \right) } \nonumber \\{} & {} \quad \times \frac{\Gamma _e\left( (pq)^{\frac{1}{2}}\left( u^Nw^2\right) ^{-2N-4}x_i q^{m_i-s_i}p^{l_i-r_i} \right) }{ \Gamma _e\left( q^{m_i-m_j+s_j-s_i}p^{l_i-l_j+r_j-r_i} x_i/x_j \right) } \Gamma _e\left( (pq)^{\frac{1}{2}}\left( uv^{N+1}\right) ^{2N+4}x_iq^{m_i} p^{l_i} \right) \nonumber \\{} & {} \quad \times \Gamma _e\left( pq \left( u^{N+1}w\right) ^{4N+8} q^{2M-m_{N+2}} p^{2L-l_{N+2}} \right) \nonumber \\{} & {} \quad \times \Gamma _e\left( q^{m_{N+2}-M}p^{l_{N+2}-L} \left( vu^{-1}\right) ^{2(N+1)(N+2)}\right) \nonumber \\{} & {} \quad \times \Gamma _e\left( (pq)^{\frac{1}{2}}u^{2(N+2)^2}q^{M-m_{N+2} + m_i-s_i} p^{L-l_{N+2}+l_i-r_i} x_i \right) \nonumber \\{} & {} \quad \times \Gamma _e\left( \left( u^{2N+5}v\right) ^{-N-1}w^{-2}a_kq^{m_{N+2}-M} p^{l_{N+2}-L} \right) {{\mathcal {I}}}_0(q^{m_i-s_i} p^{l_i-r_i}x_i). \end{aligned}$$

Summing over all partitions and using properties of $\Gamma$ and $\theta$ functions (A.9), we get the difference operator given in (2.10) with the ${\tilde{h}}_k={\tilde{h}}_{10}$. Operators obtained using another ways to close minimal punctures can be derived in absolutely identical manner and lead to the same A$\Delta$O (2.10).

## Derivation of $C_2$ operator

In this section, we give details of the derivations of results summarized in Sect. 3 and show in all details how to derive $C_2$-type generalization of the van Diejen model.

Let’s start with the derivation of the trinion that has one maximal $\textrm{USp}(2N)$ puncture, one maximal $\textrm{SU}(N+1)$ puncture and one minimal $\textrm{SU}(2)$ puncture. It is important since it will be later used by us to derive four-punctured sphere with two maximal $\textrm{USp}(2N)$ and two minimal $\textrm{SU}(2)$ punctures as specified in (3.7).

To obtain this kind of three-punctured sphere theory, we start with the trinion ${{\mathcal {T}}}^A_{x,y,z}$ with two maximal $\textrm{SU}(N+1)_{x,y}$ and one minimal $\textrm{SU}(2)_z$ punctures. This trinion was derived in [21] and is shown in Fig. 1. Corresponding superconformal index is given by:

C.1 $$\begin{aligned} K_3^{A}(x,y,z)= & {} \kappa _{N+1}\oint \prod \limits _{i=1}^{N+1}\frac{dt_i}{2\pi i t_i}\prod \limits _{i\ne j}^{N+2}\frac{1}{\Gamma _e\left( \frac{t_i}{t_j}\right) } \prod \limits _{i=1}^{N+2}\prod \limits _{j=1}^{N+1}\Gamma _e\left( (pq)^{\frac{1}{2(N+2)}}u^{2N+4}t_ix_j\right) \nonumber \\{} & {} \times \Gamma _e\left( (pq)^{\frac{1}{2(N+2)}}v^{2N+4}t_iy_j\right) \Gamma _e\left( (pq)^{\frac{1}{2(N+2)}}w^{2N+4}t_iz^{\pm 1}\right) \nonumber \\{} & {} \times \prod \limits _{l=1}^{2N+4}\Gamma _e\left( (pq)^{\frac{N+1}{2(N+2)}}\left( uv\right) ^{-N-1}w^{-2} t_i^{-1}a_l\right) , \end{aligned}$$

where *u*, *v*, *w* and $a_i$ parametrize Cartans of the 6*d* $\textrm{SO}(4N+8)$ global symmetry. $\textrm{SU}(N+2)$ gauge symmetry is parametrized by $t_i$’s with the relation

C.2 $$\begin{aligned} \prod \limits _{i=1}^{N+2}t_i=1. \end{aligned}$$

Global $\textrm{SU}(N+1)$ symmetries of the maximal punctures are parametrized by $x_i$ and $y_i$ satisfying

C.3 $$\begin{aligned} \prod \limits _{j=1}^{N+1}x_j=\prod \limits _{j=1}^{N+1}y_j=1. \end{aligned}$$

Each puncture has $(2N+6)$ moment map operators with the charges specified in (2.1).

Now in order to obtain desired trinion, we should turn one of the $\textrm{SU}(N+1)$ punctures into $\textrm{USp}(2N)$-type puncture. For this purpose, we use an $A_NC_N$ tube theory introduced in [24] and shown in Fig. 2a. Chirals of these theory correspond to the moment maps of punctures. For $\textrm{USp}(2N)$ puncture, these are just moment maps specified in (3.1). For $\textrm{SU}(N+1)$ puncture as specified in (2.1) but with the last moment map flipped so that all of them transform in the same fundamental representation of $\textrm{SU}(N+1)$:

C.4 $$\begin{aligned} M_u=\mathbf{N+1}^x\otimes \left( \mathbf{2N+4}_{u^{N+3}v^{-N-1}w^{-2}}\oplus \textbf{1}_{\left( uv^{N+1} \right) ^{2N+4}}\oplus \textbf{1}_{\left( u^N w^2\right) ^{-2N-4}}\right) \qquad \end{aligned}$$

The index of this tube theory is given by:

C.5 $$\begin{aligned} K_2^{AC}(x,{\tilde{x}})= & {} \prod \limits _{i=1}^{N+1}\prod \limits _{j=1}^N\prod \limits _{l=1}^{2N+4}\Gamma _e\left( \left( u^{-1}w\right) ^{2N+4}{\tilde{x}}_i^{-1}x_j^{\pm 1}\right) \Gamma _e\left( (pq)^{\frac{1}{2}}u^{N+3}v^{-N-1}w^{-2}a_l{\tilde{x}}_i\right) \nonumber \\{} & {} \times \Gamma _e\left( (pq)^{\frac{1}{2}}\left( uv^{N+1} \right) ^{2N+4}{\tilde{x}}_i\right) \Gamma _e\left( (pq)^{\frac{1}{2}}\left( u^Nw^2\right) ^{-2N-4}{\tilde{x}}_i\right) \nonumber \\{} & {} \times \Gamma _e\left( (pq)^{\frac{1}{2}}\left( uvw^{-2}\right) ^{N+1}a_l^{-1}x_j^{\pm 1}\right) \Gamma _e\left( (pq)^{\frac{1}{2}}\left( v^{N+1}w \right) ^{-2N-4}x_j^{\pm 1}\right) \nonumber \\{} & {} \times \Gamma _e\left( (pq)^{\frac{1}{2}}\left( u^{N+1}w\right) ^{2N+4}x_j^{\pm 1}\right) \prod \limits _{i<j}^{N+1}\Gamma _e\left( pq \left( uw^{-1}\right) ^{4(N+2)}{\tilde{x}}_i{\tilde{x}}_j\right) ,\nonumber \\ \end{aligned}$$

Now, we take this theory and glue it along the $\textrm{SU}(N+1)$ puncture to the $A_N$ trinion shown in Fig. 1. Consistent gluing in this case is the mixture of $\Phi$ and S-gluings. In particular, we $\Phi$ glue all moment maps except $\textbf{1}_{\left( u^N w^2\right) ^{2N+4}}$ which is S glued. At the level of the index, this operation corresponds to

C.6 $$\begin{aligned} K_3^{AC}(x,y,z)&=\kappa _N\oint \prod \limits _{i=1}^N\frac{d{\tilde{x}}_i}{2\pi i {\tilde{x}}_i}\prod \limits _{i\ne j}^{N+1}\frac{1}{\Gamma _e\left( \frac{{\tilde{x}}_i}{{\tilde{x}}_j}\right) }K_3^A({\tilde{x}},y,z)K_2^{AC}({\tilde{x}},x)\nonumber \\&\quad \times \prod \limits _{i=1}^{N+1}\prod \limits _{l=1}^{2N+4}\Gamma _e\left( (pq)^{\frac{1}{2}} u^{-N-3}v^{N+1}w^2a_l^{-1}{\tilde{x}}_i^{-1}\right) \nonumber \\&\quad \Gamma _e\left( (pq)^{\frac{1}{2}}\left( uv^{N+1}\right) ^{-2N-4}{\tilde{x}}_i^{-1}\right) \end{aligned}$$

Substituting expressions (C.1) for $A_N$ trinion and (C.5) for the $A_NC_N$ tube, we immediately see that the $\textrm{SU}(N+1)_{{\tilde{x}}}$ gauge theory appears to be S-confining. Corresponding S-confining theory is depicted in Fig. 8. This duality was first introduced by Spiridonov and Warnaar [44] as a mathematical identity for superconformal indices of the corresponding theories:

C.7 $$\begin{aligned}{} & {} \kappa _N\oint \prod \limits _{i=1}^N\frac{d{\tilde{x}}_i}{2\pi i {\tilde{x}}_i}\prod \limits _{i\ne j}^{N+1}\frac{1}{\Gamma _e\left( \frac{{\tilde{x}}_i}{{\tilde{x}}_j}\right) }\prod \limits _{i<j}^{N+1}\Gamma _e\left( S{\tilde{x}}_i^{-1}{\tilde{x}}_j^{-1}\right) \prod \limits _{j=1}^{N+1}\prod \limits _{k=1}^N\Gamma _e\left( {\tilde{x}}_j t_k\right) \Gamma _e\left( pq S^{-1}t_k^{-1}{\tilde{x}}_j\right) \nonumber \\{} & {} \quad \times \prod \limits _{m=1}^{N+3}\Gamma _e\left( s_m{\tilde{x}}_j^{-1}\right) =\prod \limits _{k=1}^N\prod \limits _{m=1}^{N+3}\frac{\Gamma _e\left( t_ks_m\right) }{\Gamma _e\left( St_ks_m^{-1}\right) }\prod \limits _{l<m}^{N+3}\Gamma _e\left( Ss_l^{-1}s_m^{-1}\right) , \end{aligned}$$

where $S=\prod \nolimits _{i=1}^{N+3}s_i$. Later Spiridonov and Vartanov [45] discussed physical implications of the relation written above. We refer to this duality as Spiridonov–Warnaar–Vartanov (SWV) duality. More detailed discussion about it can also been found in Appendix B.5 of our previous paper [19]. Using this duality for the $\textrm{SU}(N+1)_{{\tilde{x}}}$ node in (C.6), we can finally write down the index of the desired three-punctured sphere as follows:

C.8 $$\begin{aligned} K_3^{AC}(x,y,z)&=\kappa _{N+1}\oint \prod \limits _{i=1}^{N+1}\frac{dt_i}{2\pi it_i}\prod \limits _{i\ne j}^{N+2}\frac{1}{\Gamma _e\left( \frac{t_i}{t_j}\right) }\prod \limits _{k=1}^N \prod \limits _{i=1}^{N+2}\prod \limits _{j=1}^{N+1}\Gamma _e\left( (pq)^{\frac{1}{2(N+2)}}w^{2N+4}x_k^{\pm 1}t_i\right) \nonumber \\&\qquad \times \Gamma _e\left( (pq)^{\frac{N+1}{2(N+2)}}u^{2(N+1)(N+2)}t_i^{-1}\right) \Gamma _e\left( (pq)^{\frac{1}{2(N+2)}}v^{2N+4}y_jt_i\right) \nonumber \\&\qquad \Gamma _e\left( (pq)^{\frac{1}{2(N+2)}}w^{2N+4}t_iz^{\pm 1}\right) \nonumber \\&\qquad \times \prod \limits _{l=1}^{2N+4}\Gamma _e\left( (pq)^{\frac{N+1}{2(N+2)}}\left( uv \right) ^{-N-1}w^{-2}t_i^{-1}a_l\right) \nonumber \\&\qquad \prod \limits _{i<j}^{N+2}\Gamma _e\left( (pq)^{\frac{N+1}{N+2}}w^{-4N-8}t_i^{-1}t_j^{-1}\right) \,, \end{aligned}$$

where we have omitted some of the $\textrm{USp}(2N)$ singlets, thus redefining type of the puncture. Corresponding trinion theory is shown in Fig. 9.

Now, we can use this $A_NC_N$-type trinion theory in order to derive four-punctured sphere theory with two $\textrm{USp}(2N)$ maximal punctures. For this purpose, we S glue two copies of this trinion along $A_N$-type puncture. At the level of the index, this corresponds to the operation specified in (3.7). Substituting trinion index (C.8) into this expression, we obtain the following four-punctured sphere index:

C.9 $$\begin{aligned}&K_4(x,{\tilde{x}},z,{\tilde{z}})\nonumber \\&\quad \equiv \kappa _N\oint \prod \limits _{i=1}^N\frac{dy}{2\pi ix}\prod \limits _{i\ne j}\frac{1}{\Gamma _e\left( y_i/y_j\right) } K_3^{AC}(x,y,z){\bar{K}}_3^{AC}({\tilde{x}},y,{\tilde{z}})\nonumber \\&\quad = \kappa _{N+1}^2\kappa _N\oint \prod \limits _{i=1}^{N+1}\frac{dt_i}{2\pi it_i}\frac{d{\tilde{t}}_i}{2\pi i{\tilde{t}}_i} \prod \limits _{i=1}^N\frac{dy_i}{2\pi iy_i}\prod \limits _{i=1}^{N+2}\frac{1}{\Gamma _e\left( t_i/t_j\right) \Gamma _e\left( {\tilde{t}}_i/{\tilde{t}}_j\right) }\prod \limits _{i=1}^{N+1}\frac{1}{\Gamma _e\left( y_i/y_j\right) }\nonumber \\&\qquad \times \prod \limits _{i=1}^{N+2}\prod \limits _{j=1}^{N+1}\prod \limits _{k=1}^N\prod \limits _{l=1}^{2N+4}\Gamma _e\left( (pq)^{\frac{1}{2N+4}}w^{2N+4}x_k^{\pm 1}t_i\right) \Gamma _e\left( (pq)^{\frac{N+1}{2N+4}}u^{2(N+1)(N+2)}t_i^{-1}\right) \nonumber \\&\qquad \times \Gamma _e\left( (pq)^{\frac{1}{2N+4}}v^{2N+4}y_jt_i\right) \Gamma _e\left( (pq)^{\frac{1}{2N+4}}w^{2N+4}t_iz^{\pm 1}\right) \Gamma _e\left( (pq)^{\frac{1}{2N+4}}w^{-2N-4}{\tilde{x}}_k^{\pm 1}{\tilde{t}}_i^{-1}\right) \nonumber \\&\qquad \times \Gamma _e\left( (pq)^{\frac{N+1}{2N+4}}\left( uv\right) ^{-N-1}w^{-2}t_i^{-1}a_l\right) \Gamma _e\left( (pq)^{\frac{N+1}{2N+4}}\left( uv\right) ^{N+1}w^2{\tilde{t}}_ia_l^{-1}\right) \nonumber \\&\qquad \times \Gamma _e\left( (pq)^{\frac{N+1}{2N+4}}u^{2(N+1)(N+2)}{\tilde{t}}_i\right) \Gamma _e\left( (pq)^{\frac{1}{2N+4}}v^{-2N-4}y_j^{-1}{\tilde{t}}_i^{-1}\right) \nonumber \\&\qquad \times \Gamma _e\left( (pq)^{\frac{1}{2N+4}}w^{-2N-4}{\tilde{t}}_i^{-1}{\tilde{z}}^{\pm 1}\right) \nonumber \\&\qquad \prod \limits _{i<j}^{N+2}\Gamma _e\left( (pq)^{\frac{N+1}{N+2}}w^{-4N-8}t_i^{-1}t_j^{-1}\right) \Gamma _e\left( (pq)^{\frac{N+1}{N+2}}w^{4N+8}{\tilde{t}}_i{\tilde{t}}_j\right) \end{aligned}$$

Now, if we look on the $\textrm{SU}(N+1)_y$ gauged node, we see that it corresponds to the theory with $N+2$ hypermultiplets, meaning the node is S-confining and can be integrated out using the standard Seiberg duality. Performing this simple operation, we land on the theory shown in Fig. 10 with the following superconformal index:

C.10 $$\begin{aligned} K_4^C(x,{\tilde{x}},z,{\tilde{z}})&=\kappa ^2_{N+1}\oint \prod \limits _{i=1}^{N+1}\frac{dt_i}{2\pi it_i}\frac{d{\tilde{t}}_i}{2\pi i{\tilde{t}}_i} \prod \limits _{i\ne j}^{N+2}\frac{1}{\Gamma _e\left( t_i/t_j\right) \Gamma _e\left( {\tilde{t}}_i/{\tilde{t}}_j\right) }\nonumber \\&\quad \times \prod \limits _{i=1}^{N+2}\prod \limits _{k=1}^N\prod \limits _{l=1}^{2N+6}\Gamma _e\left( (pq)^{\frac{1}{2N+4}}w^{2N+4}x_k^{\pm 1}t_i\right) \Gamma _e\left( (pq)^{\frac{N+1}{2N+4}}w^{-2\frac{N+2}{N+3}}{\tilde{a}}_lt_i^{-1}\right) \nonumber \\&\quad \times \Gamma _e\left( (pq)^{\frac{1}{2N+4}}w^{-2N-4}{\tilde{x}}_k^{\pm 1}{\tilde{t}}_i^{-1}\right) \Gamma _e\left( (pq)^{\frac{1}{2N+4}}w^{-2N-4}{\tilde{t}}_i^{-1}{\tilde{z}}^{\pm 1}\right) \nonumber \\&\quad \times \Gamma _e\left( (pq)^{\frac{1}{2N+4}}w^{2\frac{N+2}{N+3}}{\tilde{a}}_l^{-1}{\tilde{t}}_i\right) \nonumber \\&\quad \prod \limits _{i,j=1}^{N+2}\Gamma _e\left( (pq)^{\frac{1}{N+2}}t_i{\tilde{t}}_j^{-1}\right) \Gamma _e\left( (pq)^{\frac{1}{2N+4}}w^{2N+4}t_iz^{\pm 1}\right) \nonumber \\&\quad \times \prod \limits _{i<j}^{N+2}\Gamma _e\left( (pq)^{\frac{N+1}{N+2}}w^{-4N-8}t_i^{-1}t_j^{-1}\right) \Gamma _e\left( (pq)^{\frac{N+1}{N+2}}w^{4N+8}{\tilde{t}}_i{\tilde{t}}_j\right) \,, \end{aligned}$$

where we have also used dictionary (3.4) in order to express everything in terms of $C_N$ parameterization (3.2) since now we have only this type of maximal punctures and hence latter parametrization is more natural.

Now, we finally close one of the two $\textrm{SU}(2)$ minimal punctures without introducing the defect. As discussed in Sect. 3, this amounts to computing the residue of (C.10) at the point ${\tilde{z}}=(pq)^{-\frac{1}{2}}w^{2\frac{(N+2)^2}{N+3}}{\tilde{a}}_i$. Indeed, it can be seen that at this value of ${\tilde{z}}$ there is pole coming from the contour pinching at

C.11 $$\begin{aligned} {\tilde{t}}_{N+2}=(pq)^{-\frac{N+1}{2N+4}}w^{-2\frac{N+2}{N+3}}{\tilde{a}}_i, \end{aligned}$$

where ${\tilde{t}}_{N+2}$ variable is chosen without loss of generality. In fact, we should compute such pinchings for each ${\tilde{t}}_i$ variables and sum the results. But due to the Weyl symmetry of the $\textrm{SU}(N+1)$ root system, all such contributions are the same and summing them results in an overall factor of $(N+1)!$. We omit such overall factors since they are irrelevant for the structure of the A$\Delta$O we get in the end. Computation of the corresponding residue at the pinching point leads to the following expression for the three-punctured sphere theory index:

C.12 $$\begin{aligned}&K_{(3;i;0)}^C(x,{\tilde{x}},z)\nonumber \\&\quad =\kappa _{N+1}\kappa _N\oint \prod \limits _{j=1}^{N+1}\frac{dt_j}{2\pi it_j}\prod \limits _{j=1}^N\frac{d{\tilde{t}}_j}{2\pi i{\tilde{t}}_j} \prod \limits _{k\ne j}^{N+2}\frac{1}{\Gamma _e\left( t_k/t_j\right) }\prod \limits _{i\ne j}^{N+1}\frac{1}{\Gamma _e\left( {\tilde{t}}_k/{\tilde{t}}_j\right) }\nonumber \\&\qquad \times \prod \limits _{m=1}^{N+2}\prod \limits _{j=1}^{N+1}\prod \limits _{k=1}^N\prod \limits _{l\ne i}^{2N+6} \Gamma _e\left( (pq)^{\frac{1}{2N+4}}w^{2N+4}x_k^{\pm 1}t_m\right) \Gamma _e\left( (pq)^{\frac{N+1}{2N+4}}w^{-2\frac{N+2}{N+3}}{\tilde{a}}_lt_m^{-1}\right) \nonumber \\&\qquad \times \Gamma _e\left( (pq)^{\frac{1}{2N+4}}w^{2N+4}t_mz^{\pm 1}\right) \Gamma _e\left( w^{-2\frac{(N+2)^3}{(N+1)(N+3)}}{\tilde{a}}_i^{\frac{1}{N+1}}{\tilde{x}}_k^{\pm 1}{\tilde{t}}_j^{-1}\right) \nonumber \\&\qquad \Gamma _e\left( (pq)^{\frac{1}{2}}w^{-2\frac{(N+2)^2}{N+3}}{\tilde{a}}_i^{-1}{\tilde{z}}_k^{\pm 1}\right) \Gamma _e\left( (pq)^{\frac{1}{2}}w^{2\frac{(N+2)^2}{(N+1)(N+3)}}{\tilde{a}}_l^{-1}{\tilde{a}}_i^{-\frac{1}{N+1}}{\tilde{t}}_j\right) \nonumber \\&\qquad \times \Gamma _e\left( (pq)^{\frac{1}{2N+4}}w^{-2\frac{N+2}{(N+1)(N+3)}}{\tilde{a}}_i^{\frac{1}{N+1}}t_i/{\tilde{t}}_j\right) \nonumber \\&\qquad \times \prod \limits _{i=m<j}^{N+2}\Gamma _e\left( (pq)^{\frac{N+1}{N+2}}w^{-4N-8}t_m^{-1}t_j^{-1}\right) \prod \limits _{m<j}^{N+1}\Gamma _e\left( pq w^{4\frac{(N+2)^3}{(N+1)(N+3)}}{\tilde{a}}_i^{-\frac{2}{N+1}}{\tilde{t}}_m{\tilde{t}}_j\right) \,. \end{aligned}$$

Corresponding quiver diagram is shown in Fig. 3.

In case $N=1$, it can be shown that the theory we derived reduces to the one shown in Figure 22 (b) in our previous paper [19]. There we used it to derive $C_1$ A$\Delta$O which appeared to be van Diejen operator. Studying general *N* case appears to be too complicated since there is no clear way to further simplify theory (C.12). Instead we concentrate here on $C_2$ case. When $N=2$ ${\overline{AS}}$, representation of $\textrm{SU}(N+1)$ node becomes anti-fundamental representation and the index of the theory reads:

C.13 $$\begin{aligned}&K_{(3;i,0)}^C(x,{\tilde{x}},z)\nonumber \\&\quad =\kappa _{3}\kappa _2\oint \prod \limits _{m=1}^{3}\frac{dt_m}{2\pi it_m}\prod \limits _{i=1}^2\frac{d{\tilde{t}}_m}{2\pi i{\tilde{t}}_m} \prod \limits _{m\ne j}^{4}\frac{1}{\Gamma _e\left( t_m/t_j\right) }\prod \limits _{m\ne j}^{3}\frac{1}{\Gamma _e\left( {\tilde{t}}_m/{\tilde{t}}_j\right) }\nonumber \\&\qquad \times \prod \limits _{m=1}^{4}\prod \limits _{j=1}^{3}\prod \limits _{k=1}^2\prod \limits _{l\ne i}^{10} \Gamma _e\left( (pq)^{\frac{1}{8}}w^{8}x_k^{\pm 1}t_m\right) \Gamma _e\left( (pq)^{\frac{3}{8}}w^{-\frac{8}{5}}{\tilde{a}}_lt_m^{-1}\right) \Gamma _e\left( (pq)^{\frac{1}{8}}w^{8}t_mz^{\pm 1}\right) \nonumber \\&\qquad \times \Gamma _e\left( w^{-\frac{128}{15}}{\tilde{a}}_i^{\frac{1}{3}}{\tilde{x}}_k^{\pm 1}{\tilde{t}}_j^{-1}\right) \Gamma _e\left( (pq)^{\frac{1}{2}}w^{-\frac{32}{5}}{\tilde{a}}_i^{-1}{\tilde{x}}_k^{\pm 1}\right) \Gamma _e\left( (pq)^{\frac{1}{2}}w^{\frac{32}{5}}{\tilde{a}}_l^{-1}{\tilde{a}}_i^{-\frac{1}{3}}{\tilde{t}}_j\right) \nonumber \\&\qquad \times \Gamma _e\left( (pq)^{\frac{1}{8}}w^{-\frac{8}{15}}{\tilde{a}}_i^{\frac{1}{3}}t_m/{\tilde{t}}_j\right) \Gamma _e\left( pqw^{\frac{256}{15}}{\tilde{a}}_i^{-\frac{2}{3}}{\tilde{t}}_j^{-1}\right) \prod \limits _{m<j}^{N+2}\Gamma _e\left( (pq)^{\frac{3}{4}}w^{-16}t_m^{-1}t_j^{-1}\right) \,, \end{aligned}$$

with the corresponding quiver of the theory shown in Fig. 11a.

At the next step, we perform Seiberg duality on the $\textrm{SU}(3)$ gauge node of the theory above. This node has 9 flavors so after dualizing it we obtain $\textrm{SU}(6)$ node with the superconformal index of the resulting theory given by

C.14 $$\begin{aligned}{} & {} K_{(3;i,0)}^C(x,{\tilde{x}},z)\nonumber \\{} & {} \quad =\kappa _3 \kappa _5\oint \prod \limits _{m=1}^3\frac{dt_m}{2\pi it_m}\prod \limits _{m=1}^5\frac{d{\tilde{t}}_m}{2\pi i{\tilde{t}}_m} \prod \limits _{m\ne j}^4\frac{1}{\Gamma _e\left( t_m/t_j\right) }\prod \limits _{m\ne j}^6\frac{1}{\Gamma _e\left( {\tilde{t}}_m/{\tilde{t}}_j\right) }\nonumber \\{} & {} \qquad \times \prod \limits _{m=1}^4\prod \limits _{j=1}^6\prod \limits _{k=1}^2\prod \limits _{l=2}^{10}\Gamma _e\left( (pq)^{\frac{1}{8}}w^8y_k^{\pm 1}t_m\right) \Gamma _e\left( (pq)^{\frac{1}{8}}w^8t_mz^{\pm 1}\right) \Gamma _e\left( (pq)^{\frac{1}{2}}w^{-\frac{32}{5}}{\tilde{a}}_i^{-1}{\tilde{x}}_k^{\pm 1}\right) \nonumber \\{} & {} \qquad \times \Gamma _e\left( (pq)^{\frac{1}{2}}w^{-\frac{32}{5}}{\tilde{a}}_l^{-1}{\tilde{x}}_k^{\pm 1}\right) \Gamma _e\left( (pq)^{\frac{1}{4}}w^{\frac{16}{15}}{\tilde{a}}_l{\tilde{t}}_j\right) \Gamma _e\left( (pq)^{\frac{1}{4}}w^{\frac{16}{3}}{\tilde{x}}_k^{\pm 1}{\tilde{t}}_j^{-1}\right) \nonumber \\{} & {} \qquad \times \Gamma _e\left( (pq)^{-\frac{3}{4}}w^{-\frac{304}{15}}{\tilde{a}}_i{\tilde{t}}_j^{-1}\right) \Gamma _e\left( (pq)^{\frac{1}{8}}w^{-\frac{8}{3}}t_m^{-1}{\tilde{t}}_j^{-1}\right) \prod _{m<n}^4\Gamma _e\left( (pq)^{\frac{3}{4}}w^{-16}t_m^{-1}t_n^{-1}\right) ,\nonumber \\ \end{aligned}$$

and corresponding quiver is shown in Fig. 11 (b). We see that $\textrm{SU}(4)$ gauge node of this theory appears to be S-confining due to SWV duality. Hence, using SWV index identity (C.7) we arrive to the gauge theory with the following superconformal index:

C.15 $$\begin{aligned}{} & {} K_{(3;i,0)}^C(x,{\tilde{x}},z)\nonumber \\{} & {} \quad =\kappa _5\oint \prod \limits _{i=1}^5\frac{d{\tilde{t}}_i}{2\pi i{\tilde{t}}_i}\prod \limits _{i\ne j}^6\frac{1}{\Gamma _e\left( {\tilde{t}}_i/{\tilde{t}}_j\right) } \prod \limits _{i=1}^6\prod \limits _{k=1}^2\Gamma _e\left( (pq)^{\frac{1}{4}}w^{\frac{16}{3}}x_k^{\pm 1}{\tilde{t}}_i^{-1}\right) \nonumber \\{} & {} \qquad \times \Gamma _e\left( (pq)^{\frac{1}{4}}w^{\frac{16}{3}}z^{\pm 1}{\tilde{t}}_i^{-1}\right) \Gamma _e\left( (pq)^{\frac{1}{4}}w^{\frac{16}{3}}{\tilde{x}}_k^{\pm 1}{\tilde{t}}_i^{-1}\right) \Gamma _e\left( (pq)^{-\frac{3}{4}}w^{-\frac{304}{15}}{\tilde{a}}_1{\tilde{t}}_i^{-1}\right) \nonumber \\{} & {} \qquad \times \Gamma _e\left( (pq)^{\frac{1}{2}}w^{-\frac{32}{5}}{\tilde{a}}_1^{-1}{\tilde{x}}_k^{\pm 1}\right) \prod \limits _{l=2}^{10}\Gamma _e\left( (pq)^{\frac{1}{4}}w^{\frac{16}{15}}{\tilde{a}}_l{\tilde{t}}_i\right) \Gamma _e\left( (pq)^{\frac{1}{2}}w^{-\frac{32}{5}}{\tilde{a}}_l^{-1}{\tilde{x}}_k^{\pm 1}\right) \nonumber \\{} & {} \qquad \times \prod \limits _{i<j}^6\Gamma _e\left( (pq)^{\frac{1}{2}}w^{-\frac{32}{3}}{\tilde{t}}_i{\tilde{t}}_j\right) , \end{aligned}$$

with the quiver shown in Fig. 11c. Now, we are ready to close second minimal puncture leaving us with the sphere with only two maximal $\textrm{USp}(2N)$ punctures left. As discussed in Sect. 3, for this purpose we fix *z* fugacity to be

C.16 $$\begin{aligned} z=Z^*_{i;K,0}=(pq)^{-\frac{1}{2}}w^{-\frac{32}{5}}a_i^{-1}q^{-K}. \end{aligned}$$

Carefully studying expression (C.15), we see that at this value of *z* superconformal index indeed has pole coming from the contour pinching at:

C.17 $$\begin{aligned} {\tilde{t}}_{1,2}= & {} (pq)^{\frac{1}{4}}w^{\frac{16}{3}}y_1^{\pm 1}q^{k_{1,\pm }},\quad {\tilde{t}}_{3,4}=(pq)^{\frac{1}{4}}w^{\frac{16}{3}}y_1^{\pm 1}q^{k_{2,\pm }}, \nonumber \\ {\tilde{t}}_5= & {} (pq)^{-\frac{1}{4}}w^{-\frac{16}{5}}a_i^{-1}q^{k_5-K},\quad {\tilde{t}}_6=(pq)^{-\frac{3}{4}}w^{-\frac{304}{15}}a_i q^{k_6}, \end{aligned}$$

where $\{k_{1,\pm },\,k_{2,\pm },\,k_5,\,k_6\}\equiv \vec {K}$ is partition of the integer *K*. Of course any permutation of (C.17) would also result in the same contour pinching and hence the pole of the superconformal index. Due to the Weyl group symmetry contributions of all these permutations into our operator are the same and taking them into account just results in an overall factor which we omit. Substituting values (C.17) into three-punctured sphere index (C.15), we get the following result for the index of the two-punctured sphere:

C.18 $$\begin{aligned}&K_{(2;i;K,0)}(x,{\tilde{x}})\nonumber \\&\quad =\sum \limits _{\vec {K}}C_{(\vec {K};i)}\prod \limits _{j=1}^2\prod \limits _{l\ne i}^{10}\frac{\Gamma _e\left( x_j^{\pm 2}q^{-k_{j,{\mp }}}\right) }{\Gamma _e\left( x_j^{\pm 2}q^{\pm (k_{j,+}-k_{j,-})}\right) }\nonumber \\&\qquad \times \frac{\Gamma _e\left( x_1 x_2^{\pm 1}q^{-k_{2,{\mp }}}\right) \Gamma _e\left( x_1^{-1}x_2^{\pm 1}q^{-k_{2,{\mp }}}\right) }{\Gamma _e\left( \left( x_1x_2^{\pm 1}q^{\left( k_{1,+}-k_{2,{\mp }} \right) } \right) ^{\pm 1}\right) } \frac{\Gamma _e\left( x_1^{\pm 1} x_2q^{-k_{1,{\mp }}}\right) \Gamma _e\left( x_1^{\pm 1}x_2^{-1}q^{-k_{1,{\mp }}}\right) }{\Gamma _e\left( \left( x_1^{-1}x_2^{\pm 1}q^{\left( k_{1,-}-k_{2,{\mp }} \right) } \right) ^{\pm 1}\right) }\nonumber \\&\qquad \times \frac{\Gamma _e\left( (pq)^{\frac{1}{2}}w^{\frac{32}{5}}{\tilde{a}}_ix_j^{\pm 1}q^{K-k_{j,{\mp }}}\right) }{\Gamma _e\left( \left( (pq)^{\frac{1}{2}}w^{\frac{32}{5}}{\tilde{a}}_ix_j^{\pm 1}q^{K+k_{j,\pm }-k_5}\right) ^{\pm 1}\right) } \frac{\Gamma _e\left( p^{-1}q^{-1}w^{-\frac{128}{5}}{\tilde{a}}_ix_j^{\pm 1}q^{-k_{j,{\mp }}}\right) }{\Gamma _e\left( \left( pqw^{\frac{128}{5}}{\tilde{a}}_i^{-1}x_j^{\pm 1}q^{k_{j,\pm }-k_6}\right) ^{\pm 1}\right) }\nonumber \\&\qquad \times \Gamma _e\left( (pq)^{-\frac{1}{2}}w^{-\frac{32}{5}}{\tilde{a}}_i^{-1}x_j^{\pm 1}q^{-K-k_{j,{\mp }}}\right) \nonumber \\&\qquad \Gamma _e\left( w^{-\frac{128}{5}}{\tilde{a}}_ix_j^{\pm 1}q^{k_{j,\pm }+k_6}\right) \Gamma _e\left( pqx_1x_2^{\pm 1}q^{k_{1,+}+k_{2,\pm }}\right) \nonumber \\&\qquad \times \Gamma _e\left( (pq)^{\frac{1}{2}}w^{-\frac{32}{5}}{\tilde{a}}_i^{-1}x_j^{\pm 1}q^{k_{j,\pm }+k_5-K}\right) \nonumber \\&\qquad \Gamma _e\left( pq w^{\frac{128}{5}}{\tilde{a}}_i^{-1}{\tilde{x}}_j^{\pm 1}q^{-k_6}\right) \Gamma _e\left( (pq)^{\frac{1}{2}}w^{\frac{32}{5}}{\tilde{a}}_lx_j^{\pm 1}q^{k_{j,\pm }}\right) \nonumber \\&\qquad \times \Gamma _e\left( (pq)^{\frac{1}{2}}w^{\frac{32}{5}}x_j^{\pm 1}{\tilde{a}}_iq^{K-k_5}\right) \Gamma _e\left( (pq)^{\frac{1}{2}}w^{\frac{32}{5}}{\tilde{a}}_i{\tilde{x}}_j^{\pm 1}q^{K-k_5}\right) \Gamma _e\left( (pq)^{\frac{1}{2}}w^{-\frac{32}{5}}{\tilde{a}}_l^{-1}{\tilde{x}}_j^{\pm 1}\right) \nonumber \\&\qquad \times \Gamma _e\left( (pq)^{\frac{1}{2}}w^{-\frac{32}{5}}{\tilde{a}}_i^{-1}{\tilde{x}}_j^{\pm 1}\right) \prod \limits _{k=1}^2\Gamma _e\left( {\tilde{x}}_k^{\pm 1} x_jq^{-k_{j,-}}\right) \nonumber \\&\qquad \Gamma _e\left( {\tilde{x}}_k^{\pm 1} x_j^{-1}q^{-k_{j,+}}\right) \Gamma _e\left( pq^{1+k_{1,+}+k_{1,-}}\right) \nonumber \\&\qquad \times \Gamma _e\left( pqw^{\frac{128}{5}}{\tilde{a}}_i^{-1}x_j^{\pm 1}q^{-k_6}\right) \nonumber \\&\qquad \Gamma _e\left( pqx_1^{-1}x_2^{\pm 1}q^{k_{1,-}+k_{2,\pm }}\right) \Gamma _e\left( pq^{1+k_{2,+}+k_{2,-}}\right) \,, \end{aligned}$$

where $C_{(\vec {K};i)}$ is the following constant:

C.19 $$\begin{aligned} C_{(\vec {K};i)}&=\prod \limits _{l\ne i}^{10}\Gamma _e\left( (pq)^{\frac{3}{2}}w^{32}q^{K-k_6}\right) \Gamma _e\left( {\tilde{a}}_l{\tilde{a}}_i^{-1}q^{k_5-K}\right) \Gamma _e\left( (pq)^{-\frac{1}{2}}w^{-\frac{96}{5}}{\tilde{a}}_l{\tilde{a}}_iq^{k_6}\right) \nonumber \\&\quad \times \frac{\Gamma _e\left( (pq)^{-\frac{1}{2}}w^{-\frac{96}{5}}{\tilde{a}}_i^2q^{K-k_5}\right) \Gamma _e\left( (pq)^{\frac{1}{2}}w^{\frac{96}{5}}{\tilde{a}}_i^{-2}q^{-K-k_6}\right) }{\Gamma _e\left( (pq)^{\frac{1}{2}}w^{\frac{96}{5}}{\tilde{a}}_i^{-2}q^{-K+k_5-k_6}\right) \Gamma _e\left( (pq)^{-\frac{1}{2}}w^{-\frac{96}{5}}{\tilde{a}}_i^{2}q^{K+k_6-k_5}\right) }\nonumber \\&\quad \times \Gamma _e\left( (pq)^{-\frac{1}{2}}w^{-32}q^{k_5+k_6-K}\right) \Gamma _e\left( pqw^{\frac{64}{5}}{\tilde{a}}_i^2 q^{2K-k_5}\right) \,, \end{aligned}$$

where subscript contains all the information on how the tube was obtained. Inside this subscript 2 means that we obtain two-punctured sphere (the tube) in the end. Index *i* stands for the choice of the moment map we give vev to according to (3.8) and (3.10). Finally, *K* denotes the power of holomorphic derivative of the moment map we give vev to. Summation in (C.18) goes over all possible partitions of *K* integer. Due to the presence of the defect, this index expression unlike our previous four- and three-punctured sphere does not have nice gauge theory interpretation.

Now gluing tube (C.18) to an arbitrary ${\mathcal {N}}=1$ theory with one maximal $\textrm{USp}(2N)$ puncture we can obtain desired A$\Delta$O. At the level of the superconformal index, the gluing is performed as specified in (3.13):

C.20 $$\begin{aligned} {{\mathcal {O}}}^{(C_2;h_{k};K,0)}_x\cdot {{\mathcal {I}}}(x)= & {} \frac{\kappa _N}{2^N}\oint \frac{d{\tilde{x}}_{1,2}}{2\pi i{\tilde{x}}_{1,2}}\nonumber \\{} & {} \frac{1}{\Gamma _e\left( {\tilde{x}}_{1,2}^{\pm 2}\right) \Gamma _e\left( {\tilde{x}}_1^{\pm 1}{\tilde{x}}_2^{\pm 1}\right) }K_{(2;i;K,0)}^C(x,\,{\tilde{x}}) {{\mathcal {I}}}({\tilde{x}})\nonumber \\ \end{aligned}$$

Notice that expression (C.18) for the tube index has zeroes coming from the last two $\Gamma$-functions. Due to these zeroes, only the poles of the integral in (C.20) coming from other $\Gamma$ functions will contribute to the final expression. In particular, these poles are coming from the $\Gamma _e\left( {\tilde{x}}_k^{\pm 1}x_jq^{-k_{j,-}}\right)$ and $\Gamma _e\left( {\tilde{x}}_k^{\pm 1}x_j^{-1}q^{-k_{j,+}}\right)$ terms and are located at

C.21 $$\begin{aligned} {\tilde{x}}_i=\left( x_{\sigma (i)}q^{-m_i}\right) ^{\pm 1},\qquad -k_{\sigma (i),+}\le m_i \le k_{\sigma (i),-}. \end{aligned}$$

where $\sigma (i)$ is permutation of *i*. In the end, we should sum over such permutations as well as over all combinations of $\pm 1$ powers in the expression above. Since the tube expression (C.18) is symmetric w.r.t. permutations of ${\tilde{x}}_1\leftrightarrow {\tilde{x}}_2$ and ${\tilde{x}}_i\rightarrow {\tilde{x}}_i^{-1}$, every contribution of the pinchings specified above is the same and summation just gives an irrelevant overall factor. So for simplicity we can consider only one of the combinations

C.22 $$\begin{aligned} {\tilde{x}}_i=x_i q^{-m_i},\qquad -k_{i,+}\le m_i \le k_{i,-}. \end{aligned}$$

The condition on $m_i$ comes from the fact that both types of specified $\Gamma$ functions should have poles. Half of these terms should lead to integration contour pinching in (C.20), while other half will cancel zeroes coming from the last two $\Gamma$ functions in the tube index expression (C.18).

Now computing the contribution of pinchings (C.22) into gluing (C.20) and using $\Gamma$ functions identity (A.9), we can directly obtain the full tower of operators (3.14).

## Proof of the kernel property of $A_2C_2$ tube theory

Here, we give a proof of the kernel property (4.2) where we act with $A_2$ operator (2.13) and $C_2$ operator (3.17) on the index (C.5) of the tube with $\textrm{SU}(3)$ and $\textrm{USp}(4)$ maximal punctures.

Let’s start the proof of the kernel property by explicitly calculating action of our A$\Delta$Os on the tube index and write two sides of the equation in terms of algebraic expressions. First of all we should fix the parametrization of the Cartans of global 6*d* symmetry. For this, it will be more convenient to use $C_2$ parametrization given in (3.2). We should also use the dictionary (3.4) between $A_2$ and $C_2$ parametrizations in order to rewrite everything in terms of the latter one. Fixing $N=2$ in the tube index (C.5) and writing everything in terms of $C_2$ parametrization, we obtain:

D.1 $$\begin{aligned} K_2^{A_2C_2}(x,y)= & {} \prod \limits _{i=1}^3\prod \limits _{j=1}^2\prod \limits _{l=1}^9\Gamma _e\left( w^{\frac{128}{15}}{\tilde{a}}_{10}^{-\frac{1}{3}}y_i^{-1}x_j^{\pm 1}\right) \Gamma _e\left( (pq)^{\frac{1}{2}}w^{-\frac{32}{15}}{\tilde{a}}_{10}^{\frac{1}{3}}{\tilde{a}}_ly_i\right) \nonumber \\{} & {} \times \Gamma _e\left( (pq)^{\frac{1}{2}}w^{-\frac{224}{15}}{\tilde{a}}_{10}^{-\frac{2}{3}}y_i\right) \Gamma _e\left( (pq)^{\frac{1}{2}}w^{\frac{32}{5}}{\tilde{a}}_{10}x_j^{\pm 1 }\right) \nonumber \\{} & {} \Gamma _e\left( pq w^{-\frac{256}{15}}{\tilde{a}}_{10}^{\frac{2}{3}}y_i^{-1}\right) , \end{aligned}$$

where as previously $x_{1,2}$ are fugacities for the global symmetry of the $\textrm{USp}(4)$ puncture, while $y_{1,2,3}$ are fugacities of the $\textrm{SU}(3)$ puncture. Latter ones are as usually constrained by the identity $\prod _{i=1}^3y_i=1$.

Now, we can start with the r.h.s. of (4.2) describing the action of $C_2$ operator. Main ingredient we need to find the action of the full operator is the action of the shift $\Delta _q(x_i)$ on the tube index. Studying this action in (D.1), we obtain:

D.2 $$\begin{aligned} \Delta _q(x_l)K_2^{A_2C_2}(x,y)=D_l(x,y)K_2^{A_2C_2}(x,y), \end{aligned}$$

where the function $D_l(x,y)$ is given by:

D.3 $$\begin{aligned} D_l(x,y)=\prod \limits _{i=1}^3\frac{\theta _p\left( (pq)^{\frac{1}{2}}{\tilde{a}}_{10}w^{\frac{32}{5}}x_l\right) }{\theta _p\left( (pq)^{\frac{1}{2}}q^{-1}{\tilde{a}}_{10}w^{\frac{32}{5}}x_l^{-1}\right) } \frac{\theta _p\left( {\tilde{a}}_{10}^{-\frac{1}{3}}w^{\frac{128}{15}}x_ly_i^{-1}\right) }{\theta _p\left( q^{-1}{\tilde{a}}_{10}^{-\frac{1}{3}}w^{\frac{128}{15}}x_l^{-1}y_i^{-1}\right) }\qquad \end{aligned}$$

Then, on the r.h.s. of (4.2) we obtain:

D.4 $$\begin{aligned} {{\mathcal {O}}}^{(C_2;h_{10};1,0)}_{x}K_2^{A_2C_2}(x,y)= & {} \left[ F^{(C_2;h_{10};1,0)}(x,y)+W^{(C_2;h_{10};1,0)}(x)\right] K_2^{A_2C_2}(x,y) \nonumber \\ F^{(C_2;h_{10};1,0)}(x,y)\equiv & {} \sum _{i=1}^2\left( A^{(C_2;h_{10};1,0)}_i(x)D_i(x,y)+\left( x_i\rightarrow x_i^{-1}\right) \right) \nonumber \\= & {} \sum _{i=1}^2\left( \prod \limits _{j\ne i}^2 \frac{\theta _p\left( (pq)^{\frac{1}{2}}w^{\frac{32}{5}}{\tilde{a}}_{10}x_j^{\pm 1}\right) }{\theta _p\left( x_i\right) \theta _p\left( qx_i^2\right) \theta _p\left( x_ix_j^{\pm 1}\right) } \prod \limits _{k=1}^{10}\theta _p\left( (pq)^{\frac{1}{2}}w^{-\frac{32}{5}}{\tilde{a}}_k^{-1}x_i\right) \right. \nonumber \\{} & {} \times \frac{\theta _p\left( (pq)^{\frac{1}{2}}w^{\frac{32}{5}}{\tilde{a}}_{10}x_i\right) }{\theta _p\left( (pq)^{\frac{1}{2}}w^{\frac{32}{5}}{\tilde{a}}_{10}q^{-1}x_i^{-1}\right) } \prod \limits _{l=1}^3\frac{\theta _p\left( w^{\frac{128}{15}}{\tilde{a}}_{10}^{-\frac{1}{3}}y_l^{-1}x_i\right) }{\theta _p\left( w^{\frac{128}{15}}{\tilde{a}}_{10}^{-\frac{1}{3}}y_l^{-1}q^{-1}x_i^{-1}\right) }\nonumber \\{} & {} \left. +\left( x_i\rightarrow x_i^{-1} \right) \right) . \end{aligned}$$

Now, we consider l.h.s. of the equation above. Similarly, we need to understand action of $\Delta _{lm}(y)$ operator on the $A_2C_2$ tube (4.2):

D.5 $$\begin{aligned} \Delta _{lm}(y)K_2^{A_2C_2}(x,y)=D_{lm}(x,y)K_2^{A_2C_2}(x,y), \end{aligned}$$

where

D.6 $$\begin{aligned} D_{lm}(x,y)= & {} \prod \limits _{j=1}^2\prod \limits _{k=1}^9 \frac{\theta _p\left( {\tilde{a}}_{10}^{-\frac{1}{3}}w^{\frac{128}{15}}x_j^{\pm 1}y_l^{-1}\right) }{\theta _p\left( q^{-1}{\tilde{a}}_{10}^{-\frac{1}{3}}w^{\frac{128}{15}}x_j^{\pm 1}y_m^{-1}\right) } \frac{ \theta _p\left( (pq)^{\frac{1}{2}}{\tilde{a}}_{10}^{\frac{1}{3}}w^{-\frac{32}{15}}{\tilde{a}}_k y_m\right) }{ \theta _p\left( (pq)^{\frac{1}{2}}{\tilde{a}}_{10}^{\frac{1}{3}}w^{-\frac{32}{15}}{\tilde{a}}_k q^{-1} y_l\right) } \nonumber \\{} & {} \times \frac{ \theta _p\left( (pq)^{\frac{1}{2}}{\tilde{a}}_{10}^{-\frac{2}{3}}w^{-\frac{224}{15}} y_m\right) }{ \theta _p\left( (pq)^{\frac{1}{2}}{\tilde{a}}_{10}^{-\frac{2}{3}}w^{-\frac{224}{15}} q^{-1}y_l\right) } \frac{\theta _p\left( {\tilde{a}}_{10}^{-\frac{2}{3}}w^{\frac{256}{15}}q^{-1}y_l\right) }{\theta _p\left( {\tilde{a}}_{10}^{-\frac{2}{3}}w^{\frac{256}{15}}y_m\right) } \end{aligned}$$

So the l.h.s. of (4.2) takes the following form:

D.7 $$\begin{aligned} {{\mathcal {O}}}^{(A_2;{\tilde{h}}_{10}^{-1};1,0)}_{y}K_2^{A_2C_2}(x,y)= & {} \left[ F^{(A_2;{\tilde{h}}_{10};1,0)}(x,y)+W^{(A_2;{\tilde{h}}_{10}^{-1};1,0)}(y)\right] K_2^{A_2C_2}(x,y), \nonumber \\ F^{(A_2;{\tilde{h}}_{10};1,0)}(x,y)\equiv & {} \sum \limits _{l\ne m}^3D_{lm}(x,y)A^{(A_2;{\tilde{h}}_{10};1,0)}_{lm}(y) \nonumber \\= & {} \sum \limits _{l\ne m}^3\prod \limits _{k=1}^9\prod \limits _{j=1}^2 \frac{\theta _p\left( (pq)^{\frac{1}{2}}w^{-\frac{32}{15}}{\tilde{a}}_{10}^{\frac{1}{3}}{\tilde{a}}_ky_m\right) }{\theta _p\left( q\frac{y_m}{y_l}\right) \theta _p\left( \frac{y_m}{y_l}\right) }\nonumber \\{} & {} \frac{\theta _p\left( w^{\frac{128}{15}}{\tilde{a}}_{10}^{-\frac{1}{3}}y_l^{-1}x_j^{\pm 1}\right) \theta _p\left( w^{\frac{256}{15}}{\tilde{a}}_{10}^{-\frac{2}{3}}q^{-1}y_l\right) }{\theta _p\left( w^{\frac{128}{15}}{\tilde{a}}_{10}^{-\frac{1}{3}}y_m^{-1}q^{-1}x_j^{\pm 1}\right) \theta _p\left( w^{\frac{256}{15}}{\tilde{a}}_{10}^{-\frac{2}{3}}y_m\right) }\nonumber \\{} & {} \times \theta _p\left( (pq)^{\frac{1}{2}}w^{-\frac{224}{15}}{\tilde{a}}_{10}^{-\frac{2}{3}}y_m\right) \nonumber \\{} & {} \prod \limits _{i\ne m\ne l}^3\frac{\theta _p\left( (pq)^{\frac{1}{2}}w^{\frac{224}{15}}{\tilde{a}}_{10}^{\frac{2}{3}}y_i^{-1}\right) \theta _p\left( (pq)^{\frac{1}{2}}w^{-\frac{32}{15}}{\tilde{a}}_{10}^{\frac{4}{3}}y_i\right) }{\theta _p\left( \frac{y_i}{y_l}\right) \theta _p\left( \frac{y_m}{y_i}\right) }. \nonumber \\ \end{aligned}$$

Hence, kernel property (4.2) can be reduced to the following algebraic identity:

D.8 $$\begin{aligned}{} & {} F^{(A_2;{\tilde{h}}_{10};1,0)}(x,y)+W^{(A_2;{\tilde{h}}_{10}^{-1};1,0)}(y)\nonumber \\{} & {} \quad =F^{(C_2;h_{10};1,0)}(x,y)+W^{(C_2;h_{10};1,0)}(x) \end{aligned}$$

It can be checked that both $F^{(A_2;{\tilde{h}}_{10};1,0)}(x,y)$ and $F^{(C_2;h_{10};1,0)}(x,y)$ functions defined above are elliptic w.r.t both $x_i$ and $y_i$ variables with periods 1 and *p*.^6^ In order to prove the identity, we now need to check poles and residues in the fundamental domain on two sides of equation.^7^ We already know the poles and residues of the constant parts $W^{(A_2;{\tilde{h}}_{10}^{-1};1,0)}(y)$ and $W^{(C_2;h_{10};1,0)}(x)$ summarized in Sects. 2 and 3 correspondingly. Now, let’s study functions $F^{(A_2;{\tilde{h}}_{10};1,0)}(x,y)$ and $F^{(C_2;h_{10};1,0)}(x,y)$ coming from the action of the shift parts.

We start with $F^{(A_2;{\tilde{h}}_{10};1,0)}(x,y)$ function given in (D.7). This elliptic function appears to have poles at the following positions:

D.9 $$\begin{aligned} y_a= & {} w^{\frac{128}{15}}{\tilde{a}}_{10}^{-\frac{1}{3}}q^{-1}x_b^s,\quad y_a=q y_b,\quad y_a=q^{-1}y_b,\quad y_a=sq^{\frac{1}{2}}P_a^{-\frac{1}{2}},\quad y_a=sq^{-\frac{1}{2}}P_a^{-\frac{1}{2}}, \nonumber \\ y_a= & {} sq^{\frac{1}{2}}p^{\frac{1}{2}}P_a^{-\frac{1}{2}},\quad y_a=sq^{-\frac{1}{2}}p^{\frac{1}{2}}P_a^{-\frac{1}{2}},\quad x_j=\left( w^{\frac{128}{15}}{\tilde{a}}_{10}^{-\frac{1}{3}}q^{-1}y_a^{-1}\right) ^{\pm 1}, \end{aligned}$$

where $s=\pm$ and $P_a\equiv \prod \limits _{j\ne a}^3 y_j$. Seemingly there are more poles in expression (D.7) but checking all residues shows that the only actual poles are the ones listed above.

Residues of poles (D.9) are given by

D.10 $$\begin{aligned}&\textrm{Res}_{y_a=w^{\frac{128}{15}}{\tilde{a}}_{10}^{-\frac{1}{3}}q^{-1}x_b^s}F^{(A_2;{\tilde{h}}_{10};1,0)}(x,y)\nonumber \\&\quad =\frac{w^{\frac{128}{15}} {\tilde{a}}_{10}^{-\frac{1}{3}}q^{-1}x_b^s}{\left( p;p\right) _\infty ^2} \sum \limits _{l\ne a}^3\prod \limits _{k=1}^9\frac{\theta _p\left( (pq)^{\frac{1}{2}}w^{\frac{32}{5}}{\tilde{a}}_kq^{-1}x_b^s\right) }{\theta _p\left( w^{\frac{128}{5}}q_{10}^{-1}q^{-1}x_b^s\right) }\nonumber \\&\qquad \times \frac{\theta _p\left( w^{\frac{256}{15}}{\tilde{a}}_{10}^{-\frac{2}{3}}q^{-1}y_l\right) }{\theta _p\left( w^{\frac{128}{15}}{\tilde{a}}_{10}^{-\frac{1}{3}}q^{-1}y_l^{-1}x_b^s\right) } \prod \limits _{i\ne l\ne a}^3\frac{\theta _p\left( (pq)^{\frac{1}{2}}w^{\frac{224}{15}}{\tilde{a}}_{10}^{\frac{2}{3}}y_i^{-1}\right) \theta _p\left( (pq)^{\frac{1}{2}}w^{-\frac{32}{15}}{\tilde{a}}_{10}^{\frac{4}{3}}y_i\right) }{\theta _p\left( \frac{y_i}{y_l}\right) \theta _p\left( w^{\frac{128}{15}}q^{-1}{\tilde{a}}_{10}^{-\frac{1}{3}}y_i^{-1}x_b^s\right) }\nonumber \\&\qquad \times \prod \limits _{j\ne b}^2\frac{\theta _p\left( w^{\frac{128}{15}}{\tilde{a}}_{10}^{-\frac{1}{3}}y_l^{-1}x_j^{\pm 1}\right) \theta _p\left( w^{\frac{128}{15}}{\tilde{a}}_{10}^{-\frac{1}{3}}y_l^{-1}x_b^{-s}\right) }{\theta _p\left( x_b^{-s}x_j^{\pm 1} \right) \theta _p\left( x_b^{-2s}\right) }\theta _p\left( (pq)^{\frac{1}{2}}w^{-\frac{32}{15}}{\tilde{a}}_{10}^{-1}q^{-1}x_b^s \right) \,, \end{aligned}$$

D.11 $$\begin{aligned}&\textrm{Res}_{x_j=\left( w^{\frac{128}{15}}{\tilde{a}}_{10}^{-\frac{1}{3}}q^{-1}y_a^{-1}\right) ^{s}}F^{(A_2;{\tilde{h}}_{10};1,0)}(x,y)\nonumber \\&\quad = s\frac{\left( w^{\frac{128}{15}}{\tilde{a}}_{10}^{-\frac{1}{3}}q^{-1}y_a^{-1}\right) ^{s}}{\left( p;p\right) _\infty ^2}\nonumber \\&\qquad \times \sum _{l\ne a}^3\prod \limits _{k=1}^9\prod \limits _{i\ne j}^2\frac{\theta _p\left( (pq)^{\frac{1}{2}}w^{-\frac{32}{15}}{\tilde{a}}_{10}^{\frac{1}{3}}{\tilde{a}}_ky_a\right) }{\theta _p\left( q\frac{y_a}{y_l}\right) \theta _p\left( \frac{y_a}{y_l}\right) } \frac{\theta _p\left( w^{\frac{128}{15}}{\tilde{a}}_{10}^{-\frac{1}{3}}y_l^{-1}x_i^{\pm 1}\right) }{\theta _p\left( w^{\frac{128}{15}}{\tilde{a}}_{10}^{-\frac{1}{3}}y_a^{-1}q^{-1}x_i^{\pm 1}\right) }\nonumber \\&\qquad \times \frac{\theta _p\left( w^{\frac{256}{15}}{\tilde{a}}_{10}^{-\frac{2}{3}}q^{-1}y_l^{-1}y_a^{-1}\right) \theta _p\left( w^{\frac{256}{15}}{\tilde{a}}_{10}^{-\frac{2}{3}}q^{-1}y_l\right) }{\theta _p\left( w^{\frac{256}{15}}{\tilde{a}}_{10}^{-\frac{2}{3}}q^{-2}y_a^{-2}\right) \theta _p\left( w^{\frac{256}{15}}{\tilde{a}}_{10}^{-\frac{2}{3}}y_a\right) } \theta _p\left( (pq)^{\frac{1}{2}}w^{-\frac{224}{15}}{\tilde{a}}_{10}^{-\frac{2}{3}}y_a\right) \nonumber \\&\qquad \times \theta _p\left( qy_l^{-1}y_a\right) \prod \limits _{k\ne a\ne l}^3\frac{\theta _p\left( (pq)^{\frac{1}{2}}w^{\frac{224}{15}}{\tilde{a}}_{10}^{\frac{2}{3}}y_k^{-1}\right) \theta _p\left( (pq)^{\frac{1}{2}}w^{-\frac{32}{15}}{\tilde{a}}_{10}^{\frac{4}{3}}y_k\right) }{\theta _p\left( \frac{y_k}{y_l}\right) \theta _p\left( \frac{y_a}{y_k}\right) } \end{aligned}$$

D.12 $$\begin{aligned}&\textrm{Res}_{y_a=qy_b}F^{(A_2;{\tilde{h}}_{10};1,0)}(x,y)\nonumber \\&\quad =\frac{qy_b}{\theta _p\left( q^{-1}\right) \left( p;p\right) _\infty ^2} \prod \limits _{k=1}^9\theta _p\left( (pq)^{\frac{1}{2}}w^{-\frac{32}{15}}{\tilde{a}}_{10}^{\frac{1}{3}}{\tilde{a}}_ky_b\right) \nonumber \\&\qquad \times \theta _p\left( (pq)^{\frac{1}{2}}w^{-\frac{224}{15}}{\tilde{a}}_{10}^{-\frac{2}{3}}y_b\right) \prod \limits _{j\ne a\ne b}^3\frac{\theta _p\left( (pq)^{\frac{1}{2}}w^{\frac{224}{15}}{\tilde{a}}_{10}^{\frac{2}{3}}y_j^{-1}\right) \theta _p\left( (pq)^{\frac{1}{2}}w^{-\frac{32}{15}}{\tilde{a}}_{10}^{\frac{4}{3}}y_j\right) }{\theta _p\left( q^{-1}\frac{y_j}{y_b}\right) \theta _p\left( \frac{y_b}{y_j}\right) }\,, \end{aligned}$$

D.13 $$\begin{aligned}&\textrm{Res}_{y_a=q^{-1}y_b}F^{(A_2;{\tilde{h}}_{10};1,0)}(x,y)\nonumber \\&\quad =-\frac{q^{-1}y_b}{\theta _p\left( q^{-1}\right) \left( p;p\right) _\infty ^2}\prod \limits _{k=1}^9 \theta _p\left( (pq)^{\frac{1}{2}}w^{\frac{32}{15}}{\tilde{a}}_{10}^{-\frac{1}{3}}{\tilde{a}}_k^{-1}y_b^{-1}\right) \nonumber \\&\qquad \times \theta _p\left( (pq)^{\frac{1}{2}}w^{\frac{224}{15}}{\tilde{a}}_{10}^{\frac{2}{3}}y_b^{-1}\right) \prod \limits _{j\ne a\ne b}^3\frac{\theta _p\left( (pq)^{\frac{1}{2}}w^{\frac{224}{15}}{\tilde{a}}_{10}^{\frac{2}{3}}y_j^{-1}\right) \theta _p\left( (pq)^{\frac{1}{2}}w^{-\frac{32}{15}}{\tilde{a}}_{10}^{\frac{4}{3}}y_j\right) }{\theta _p\left( \frac{y_j}{y_b}\right) \theta _p\left( q^{-1}\frac{y_b}{y_j}\right) }\,, \end{aligned}$$

D.14 $$\begin{aligned}&\textrm{Res}_{y_a=sq^{\frac{1}{2}}P_a^{-\frac{1}{2}}}F^{(A_2;{\tilde{h}}_{10};1,0)}(x,y)\nonumber \\&\quad = s\frac{q^{\frac{1}{2}}P_a^{-\frac{1}{2}}}{2\theta _p\left( q^{-1}\right) \left( p;p\right) _\infty ^2} \prod \limits _{k=1}^9\theta _p\left( sp^{\frac{1}{2}}w^{-\frac{32}{15}}{\tilde{a}}_{10}^{\frac{1}{3}}{\tilde{a}}_kP_a^{-\frac{1}{2}}\right) \nonumber \\&\qquad \times \theta _p\left( sp^{\frac{1}{2}}w^{-\frac{224}{15}}{\tilde{a}}_{10}^{-\frac{2}{3}}P_a^{-\frac{1}{2}}\right) \frac{\theta _p\left( (pq)^{\frac{1}{2}} w^{\frac{224}{15}}{\tilde{a}}_{10}^{\frac{2}{3}}P_a^{-1}\right) \theta _p\left( (pq)^{\frac{1}{2}} w^{-\frac{32}{15}}{\tilde{a}}_{10}^{\frac{4}{3}}P_a\right) }{\theta _p\left( sq^{-\frac{1}{2}}P_a^{\frac{3}{2}}\right) \theta _p\left( sq^{-\frac{1}{2}}P_a^{-\frac{3}{2}}\right) }\,, \end{aligned}$$

D.15 $$\begin{aligned}&\textrm{Res}_{y_a=sq^{-\frac{1}{2}}P_a^{-\frac{1}{2}}}F^{(A_2;{\tilde{h}}_{10};1,0)}(x,y)\nonumber \\&\quad = -s\frac{q^{-\frac{1}{2}}P_a^{-\frac{1}{2}}}{2\theta _p\left( q^{-1}\right) \left( p;p\right) _\infty ^2} \prod \limits _{k=1}^9\theta _p\left( sp^{\frac{1}{2}}w^{-\frac{32}{15}}{\tilde{a}}_{10}^{\frac{1}{3}}{\tilde{a}}_kP_a^{-\frac{1}{2}}\right) \nonumber \\&\qquad \times \theta _p\left( sp^{\frac{1}{2}}w^{-\frac{224}{15}}{\tilde{a}}_{10}^{-\frac{2}{3}}P_a^{-\frac{1}{2}}\right) \frac{\theta _p\left( (pq)^{\frac{1}{2}} w^{\frac{224}{15}}{\tilde{a}}_{10}^{\frac{2}{3}}P_a^{-1}\right) \theta _p\left( (pq)^{\frac{1}{2}} w^{-\frac{32}{15}}{\tilde{a}}_{10}^{\frac{4}{3}}P_a\right) }{\theta _p\left( sq^{-\frac{1}{2}}P_a^{\frac{3}{2}}\right) \theta _p\left( sq^{-\frac{1}{2}}P_a^{-\frac{3}{2}}\right) }\,, \end{aligned}$$

D.16 $$\begin{aligned}&\textrm{Res}_{y_a=sq^{\frac{1}{2}}p^{\frac{1}{2}}P_a^{-\frac{1}{2}}}F^{(A_2;{\tilde{h}}_{10};1,0)}(x,y)\nonumber \\&\quad = s\frac{p^{\frac{3}{2}}q^{\frac{1}{2}}P_a^{\frac{1}{2}}w^{\frac{256}{15}}{\tilde{a}}_{10}^{-\frac{2}{3}}}{2\theta _p\left( q^{-1}\right) \left( p;p\right) _\infty ^2} \prod \limits _{k=1}^9\theta _p\left( sw^{-\frac{32}{15}}{\tilde{a}}_{10}^{\frac{1}{3}}{\tilde{a}}_kP_a^{-\frac{1}{2}}\right) \nonumber \\&\qquad \times \theta _p\left( sw^{-\frac{224}{15}}{\tilde{a}}_{10}^{-\frac{2}{3}}P_a^{-\frac{1}{2}}\right) \frac{\theta _p\left( (pq)^{\frac{1}{2}} w^{\frac{224}{15}}{\tilde{a}}_{10}^{\frac{2}{3}}P_a^{-1}\right) \theta _p\left( (pq)^{\frac{1}{2}} w^{-\frac{32}{15}}{\tilde{a}}_{10}^{\frac{4}{3}}P_a\right) }{\theta _p\left( sq^{-\frac{1}{2}}p^{\frac{1}{2}}P_a^{\frac{3}{2}}\right) \theta _p\left( sq^{-\frac{1}{2}}p^{-\frac{1}{2}}P_a^{-\frac{3}{2}}\right) }\,, \end{aligned}$$

D.17 $$\begin{aligned}&\textrm{Res}_{y_a=sq^{-\frac{1}{2}}p^{\frac{1}{2}}P_a^{-\frac{1}{2}}}F^{(A_2;{\tilde{h}}_{10};1,0)}(x,y)\nonumber \\&\quad = -s\frac{p^{\frac{3}{2}}q^{-\frac{1}{2}}P_a^{\frac{1}{2}}w^{\frac{256}{15}}{\tilde{a}}_{10}^{-\frac{2}{3}}}{2\theta _p\left( q^{-1}\right) \left( p;p\right) _\infty ^2} \prod \limits _{k=1}^9\theta _p\left( sw^{-\frac{32}{15}}{\tilde{a}}_{10}^{\frac{1}{3}}{\tilde{a}}_kP_a^{-\frac{1}{2}}\right) \nonumber \\&\qquad \times \theta _p\left( sw^{-\frac{224}{15}}{\tilde{a}}_{10}^{-\frac{2}{3}}P_a^{-\frac{1}{2}}\right) \frac{\theta _p\left( (pq)^{\frac{1}{2}} w^{\frac{224}{15}}{\tilde{a}}_{10}^{\frac{2}{3}}P_a^{-1}\right) \theta _p\left( (pq)^{\frac{1}{2}} w^{-\frac{32}{15}}{\tilde{a}}_{10}^{\frac{4}{3}}P_a\right) }{\theta _p\left( sq^{-\frac{1}{2}}p^{\frac{1}{2}}P_a^{\frac{3}{2}}\right) \theta _p\left( sq^{-\frac{1}{2}}p^{-\frac{1}{2}}P_a^{-\frac{3}{2}}\right) }\,, \end{aligned}$$

Looking on these residues, we immediately see that on the l.h.s. of (D.8) there are vast cancelation of residues between $F^{(A_2;{\tilde{h}}_{10};1,0)}(x,y)$ and constant part $W^{(A_2;{\tilde{h}}_{10}^{-1};1,0)}(y)$. Latter ones can be found from (2.18) upon charges identification given in (3.4). In particular, the poles at $y_a=q y_b\,, y_a=q^{-1}y_b\,,$ $y_a=sq^{\frac{1}{2}}P_a^{-\frac{1}{2}}\,,$ $y_a=sq^{-\frac{1}{2}}P_a^{-\frac{1}{2}},$ $y_a=sq^{\frac{1}{2}}p^{\frac{1}{2}}P_a^{-\frac{1}{2}},$ $y_a=sq^{-\frac{1}{2}}p^{\frac{1}{2}}P_a^{-\frac{1}{2}},$ get canceled. Hence, we are left with the following poles and residues on the l.h.s. of (D.8):

Now, let’s move to the $C_2$ side of the kernel function Eq. (D.8). As mentioned previously the function $F^{(C_2;{\tilde{h}}_{10};1,0)}(x,y)$ defined in (D.4) is elliptic with periods 1 and *p*. In the fundamental domain, the poles of the function are located at

D.20 $$\begin{aligned} y_a=w^{\frac{128}{15}}{\tilde{a}}_{10}^{-\frac{1}{3}}q^{-1}x_b^s,\quad x_i=sq^{\pm \frac{1}{2}},\quad x_i=sq^{\pm \frac{1}{2}}p^{\frac{1}{2}}, \quad x_j=\left( w^{\frac{128}{15}}{\tilde{a}}_{10}^{-\frac{1}{3}}q^{-1}y_a^{-1}\right) ^{\pm 1}, \nonumber \\ \end{aligned}$$

where as usually $s=\pm 1$. The residues at the poles $x_i=sq^{\pm \frac{1}{2}}$ and $x_i=sq^{\pm \frac{1}{2}}p^{\frac{1}{2}}$ are given by

D.21 $$\begin{aligned}&\textrm{Res}_{x_i=sq^{\frac{1}{2}}}F^{(C_2;h_{10};1,0)}(x,y)\nonumber \\&\quad =s\frac{q^{\frac{1}{2}}\prod \limits _{k=1}^{10} \theta _p\left( (pq)^{\frac{1}{2}}sq^{-\frac{1}{2}}w^{-\frac{32}{5}}{\tilde{a}}_k^{-1}\right) }{2\left( p;p\right) _\infty ^2\theta _p\left( q^{-1}\right) }\prod \limits _{j\ne i}^2 \frac{\theta _p\left( (pq)^{\frac{1}{2}} w^{\frac{32}{5}}{\tilde{a}}_{10} x_j^{\pm 1}\right) }{\theta _p\left( sq^{-\frac{1}{2}}x_j^{\pm 1} \right) }\,, \nonumber \\&\textrm{Res}_{x_i=sq^{-\frac{1}{2}}}F^{(C_2;h_{10};1,0)}(x,y)\nonumber \\&\quad =-s\frac{q^{-\frac{1}{2}} \prod \limits _{k=1}^{10}\theta _p\left( (pq)^{\frac{1}{2}}sq^{-\frac{1}{2}}w^{-\frac{32}{5}}{\tilde{a}}_k^{-1}\right) }{2\left( p;p\right) _\infty ^2\theta _p\left( q^{-1}\right) }\prod \limits _{j\ne i}^2 \frac{\theta _p\left( (pq)^{\frac{1}{2}} w^{\frac{32}{5}}{\tilde{a}}_{10} x_j^{\pm 1}\right) }{\theta _p\left( sq^{-\frac{1}{2}}x_j^{\pm 1} \right) }\,, \nonumber \\&\textrm{Res}_{x_i=sp^{\frac{1}{2}}q^{\frac{1}{2}}}F^{(C_2;h_{10};1,0)}(x,y)\nonumber \\&\quad =s\frac{pw^{-32}\prod \limits _{k=1}^{10}\theta _p\left( sw^{\frac{32}{5}}{\tilde{a}}_k\right) }{2\left( p;p\right) _\infty ^2\theta _p\left( q^{-1}\right) }\prod \limits _{j\ne i}^2 \frac{\theta _p\left( (pq)^{\frac{1}{2}}w^{\frac{32}{5}}{\tilde{a}}_{10}x_j^{\pm 1}\right) }{\theta _p\left( (pq)^{-\frac{1}{2}}sx_j^{\pm 1} \right) }\,, \nonumber \\&\textrm{Res}_{x_i=sp^{\frac{1}{2}}q^{-\frac{1}{2}}}F_C^{(C_2;h_{10};1,0)}(x,y)\nonumber \\&\quad =-s\frac{pq^{-1}w^{-32} \prod \limits _{k=1}^{10}\theta _p\left( sw^{\frac{32}{5}}{\tilde{a}}_k\right) }{2\left( p;p\right) _\infty ^2\theta _p\left( q^{-1}\right) }\prod \limits _{j\ne i}^2 \frac{\theta _p\left( (pq)^{\frac{1}{2}}w^{\frac{32}{5}}{\tilde{a}}_{10}x_j^{\pm 1}\right) }{\theta _p\left( (pq)^{-\frac{1}{2}}sx_j^{\pm 1} \right) }\,. \end{aligned}$$

It can be easily seen that these residues are canceled by the corresponding residues (3.19) of the constant part $W^{(C_2;h_{10};1,0)}$. Hence, $C_2$ side of kernel function Eq. (D.8) does not have poles in $x_i=sq^{\pm \frac{1}{2}}$ and $x_i=sq^{\pm \frac{1}{2}}p^{\frac{1}{2}}$. The only poles are the same as on the $A_2$ side and are located in $y_a=w^{\frac{128}{15}}{\tilde{a}}_{10}^{-\frac{1}{3}}q^{-1}x_b^s$ and $x_j=\left( w^{\frac{128}{15}}{\tilde{a}}_{10}^{-\frac{1}{3}}q^{-1}y_a^{-1}\right) ^{\pm 1}$. Corresponding residues of $F^{(C_2;h_{10};1,0)}$ and hence of the full function $F^{(C_2;h_{10};1,0)}+W^{(C_2;h_{10};1,0)}$ are given by As the next step, we have to show that the residues in (D.22) and (D.23) coincide with the residues (D.18) and (D.19) in order for the kernel identity (D.8) to work. Notice that in both pairs (D.22),(D.23) and (D.18), (D.19) position of the poles is defined by a single equation

D.24 $$\begin{aligned} w^{\frac{128}{15}}{\tilde{a}}_{10}^{-\frac{1}{3}}q^{-1}x_b^s y_a^{-1}=1 \end{aligned}$$

The two residues in each of the pairs differ by the choice of the variables with respect to which we compute this residue. It is either fixing $x_a$ variable and computing residue in $y_a$ variable (like in (D.18) and (D.22)) or vice versa (like in (D.19) and (D.23)). Hence, it is sufficient to prove equality of residues only in one of the variables. It then automatically works for another variable since it is the very same pole. Let’s choose to study residues w.r.t. $x_b$.^8^ Also since all expressions are symmetric w.r.t. $x_i\rightarrow x_i^{-1}$, we can perform the check only for one of the signs *s*. The other one will work automatically. So let’s perform the check only for the residue at $x_a=w^{\frac{128}{15}}{\tilde{a}}_{10}^{-\frac{1}{3}}q^{-1}y_b^{-1}$ Subtracting residue (D.19) from the residue (D.23), we can obtain the following equation:

D.25 $$\begin{aligned}&\textrm{Res}_{x_a=w^{\frac{128}{15}}{\tilde{a}}_{10}^{-\frac{1}{3}}q^{-1}y_b^{-1}}\left[ F^{(C_2;h_{10};1,0)}(x,y)+ W^{(C_2;h_{10};1,0)}(x)-F^{(A_2,{\tilde{h}}_{10};1,0)}(x,y) \right. \nonumber \\&\quad \qquad \left. -W^{(A_2;{\tilde{h}}_{10}^{-1};1,0)}(y)\right] = \frac{w^{\frac{128}{15}}{\tilde{a}}_{10}^{-\frac{1}{3}}y_b^{-1}q^{-1}}{\left( p;p\right) _\infty ^2}\prod \limits _{l\ne b}^3\frac{\theta _p\left( w^{\frac{256}{15}}{\tilde{a}}_{10}^{-\frac{2}{3}}q^{-1}y_l\right) }{\theta _p\left( \frac{y_b}{y_l}\right) } \frac{\theta _p\left( (pq)^{\frac{1}{2}}w^{\frac{224}{15}}{\tilde{a}}_{10}^{\frac{2}{3}}y_b\right) }{\theta _p\left( w^{\frac{256}{15}}{\tilde{a}}_{10}^{-\frac{2}{3}}q^{-2}y_b^{-2}\right) }\nonumber \\&\quad \qquad \times \prod \limits _{j\ne a}^2\frac{\theta _p\left( (pq)^{\frac{1}{2}}w^{\frac{32}{5}}{\tilde{a}}_{10}x_j^{\pm 1}\right) }{\theta _p\left( w^{\frac{128}{15}}{\tilde{a}}_{10}^{-\frac{1}{3}}q^{-1}y_b^{-1}x_j^{\pm 1}\right) }\left[ 1-M_{ab}(x,y) \right] \,, \end{aligned}$$

where we have introduced an auxiliary function

D.26 $$\begin{aligned} M_{ab}(x,y)&=\sum \limits _{l\ne b}^3\prod \limits _{j\ne a}^2\frac{\theta _p\left( w^{\frac{128}{15}}{\tilde{a}}_{10}^{-\frac{1}{3}}y_l^{-1}x_j^{\pm 1} \right) }{\theta _p\left( (pq)^{\frac{1}{2}}w^{\frac{32}{5}}{\tilde{a}}_{10}x_j^{\pm 1} \right) } \nonumber \\&\quad \times \prod \limits _{k\ne l\ne b}^3 \frac{\theta _p\left( (pq)^{\frac{1}{2}}w^{\frac{224}{15}}{\tilde{a}}_{10}^{\frac{2}{3}}y_k^{-1} \right) \theta _p\left( (pq)^{\frac{1}{2}}w^{-\frac{32}{15}}{\tilde{a}}_{10}^{\frac{4}{3}}y_k \right) }{\theta _p\left( \frac{y_k}{y_l}\right) \theta _p\left( w^{\frac{256}{15}}{\tilde{a}}_{10}^{-\frac{2}{3}}y_b\right) }\,. \end{aligned}$$

It can be checked that this function is elliptic with periods 1 and *p*. Seemingly this function has poles at $x_j=\left[ (pq)^{\frac{1}{2}}w^{\frac{32}{5}}{\tilde{a}}_{10}\right] ^{\pm 1}$, $y_a=y_c$ with $a,c\ne b$ and $y_b=w^{-\frac{256}{15}}{\tilde{a}}_{10}^{\frac{2}{3}}$. However, as it often happens in our calculations, accurate derivation of the corresponding residues results in zeros for all of them.^9^ Hence, these are not really poles and our function *M*(*x*, *y*) is an entire function for all its variables. Since function is entire elliptic function, we conclude that it is just a constant.

In order to define which particular constant it is, we just need to put in some values for *x* and *y* variables. Here, it is worth noticing that *M*(*x*, *y*) function is the sum of two products of $\theta$ function. Without loss of generality, let’s choose the following values of *y* variables:

D.27 $$\begin{aligned} y_c=(pq)^{-\frac{1}{2}}w^{\frac{32}{15}}{\tilde{a}}_{10}^{-\frac{4}{3}},\quad y_d=(pq)^{\frac{1}{2}}w^{-\frac{32}{15}}{\tilde{a}}_{10}^{\frac{4}{3}}y_b^{-1}, \end{aligned}$$

where the *c* and *d* indices are any indices not equal to *b*. The second equality, i.e., value of $y_d$, follows from the first one and $A_2$ constraint for $y_j$ variables. Now, if we look on the definition (D.26) of *M*(*x*, *y*), we see the sum of two terms. First term is when $l=c, k=d$, and the second term is vice versa when $l=d, k=c$. However, we immediately see that if $y_k=(pq)^{-\frac{1}{2}}w^{\frac{32}{15}}{\tilde{a}}_{10}^{-\frac{4}{3}}$ in (D.26), we obtain zero of theta function. Hence, this second term in the sum is automatically zero and that was the idea behind our choice (D.27). We are now left only with the first term. Substituting chosen values into this term, we see vast cancelations leading to

D.28 $$\begin{aligned} \left. M_{ab}(x,y)\right| _{y_c=(pq)^{-\frac{1}{2}}w^{\frac{32}{15}}{\tilde{a}}_{10}^{-\frac{4}{3}}}=1, \end{aligned}$$

and hence the $M_{ab}(x,y)$ function given in (D.26) is just 1, i.e., $M_{ab}(x,y)=1$. Notice that to find the value of the constant $M_{ab}(x,y)$ we fixed only one of *y* variables. Other *y*’s as well as all of *x* stayed random. Using our finding from (D.25), we immediately conclude that

D.29 $$\begin{aligned}&\textrm{Res}_{x_a=w^{\frac{128}{15}}{\tilde{a}}_{10}^{-\frac{1}{3}}q^{-1}y_b^{-1}} \left[ F^{(C_2;h_{10};1,0)}(x,y)+W^{(C_2;h_{10};1,0)}(x)\right] \nonumber \\&\quad =\textrm{Res}_{x_a=w^{\frac{128}{15}}{\tilde{a}}_{10}^{-\frac{1}{3}}q^{-1}y_b^{-1}} \left[ F^{(A_2;{\tilde{h}}_{10};1,0)}(x,y)+W^{(A_2;{\tilde{h}}_{10}^{-1};1,0)}(y)\right] \,. \end{aligned}$$

Now since both functions $F^{(C_2;h_{10};1,0)}(x,y)+W^{(C_2;h_{10};1,0)}(x)$ and $F^{(A_2;{\tilde{h}}_{10};1,0)}(x,y)+W^{(A_2;{\tilde{h}}_{10}^{-1};1,0)}(y)$ are elliptic with the coinciding poles and residues in the fundamental domain, we conclude that they can differ at most by additive constant. And just as we did it with $M_{ab}(x,y)$ function in order to find this constant, we need to choose convenient values of *x* and *y* variables. Here, it is a bit harder to do then in the case of $M_{ab}$ function. However, first thing we can notice is that constant part $W^{(C_2;h_{10};1,0)}(x)$ of $C_2$ operator (3.17) has zero at the following value of variables:

D.30 $$\begin{aligned} x_1=(pq)^{\frac{1}{2}}w^{\frac{32}{5}}{\tilde{a}}_{10}, \end{aligned}$$

where we have chosen $x_1$ variable without loss of generality (it can as well be $x_2$ variable) and the other $x_2$ variable is kept arbitrary. At the same time, constant part $W^{(A_2;{\tilde{h}}_{10}^{-1};1,0)}(y)$ of $A_2$ operator given in (2.15) has zero at the following value of variables:

D.31 $$\begin{aligned} y_1=(pq)^{\frac{1}{2}}w^{\frac{224}{15}}{\tilde{a}}_{10}^{\frac{2}{3}},\quad y_2=(pq)^{-\frac{1}{2}}w^{\frac{32}{15}}{\tilde{a}}_{10}^{-\frac{4}{3}},\quad y_3=w^{-\frac{256}{15}}{\tilde{a}}_{10}^{\frac{2}{3}}. \end{aligned}$$

It is natural to try these values and check what happens with the shift parts $F^{(A_2;{\tilde{h}}_{10};1,0)}(y)$ and $F^{(C_2;h_{10};1,0)}(x)$ on two sides of the kernel identity (D.8). On the $C_2$ side, it is straightforward to see from (D.4) that simultaneous substitution of values specified in (D.31) and (D.30) leads to zero of the shift part $F^{(C_2;h_{10};1,0)}(x,y)$. With the $A_2$ side, it is a bit more tricky since the shift part $F^{(A_2;{\tilde{h}}_{10};1,0)}(x,y)$ seemingly has singularity at $y_3=w^{-\frac{256}{15}}{\tilde{a}}_{10}^{\frac{2}{3}}$ due to theta function $\theta _p\left( w^{\frac{256}{15}}{\tilde{a}}_{10}^{-\frac{2}{3}}\right)$ in the denominator of the expression. However, accurate analysis shows that this singularity is always canceled by zeros of other theta functions standing in the numerator of the expression. Taking this into account, it is once again pretty easy to show that shift part on $A_2$ side of the kernel property also has zero at values (D.31) and (D.30).

Summarizing we have shown that expressions on two sides of the kernel property Eq. (D.8) are elliptic functions with periods 1 and *p* and same sets of poles and residues in the fundamental domain given in (D.18), (D.19), (D.22) and (D.23). This means that the two functions differ at most by constant. To fix this constant, we also notice that both functions on two sides of the equation have zero at the same values of variables given in (D.31) and (D.30). Hence, the constant two functions can differ by is just zero and the functions appear to be the same. This concludes the proof of the kernel property (4.2).

## Commutators of $C_2$ operators

In this appendix, we discuss commutation relations (4.5) of the basic $C_2$ operators (3.17). Let’s start with checking the following commutation relation:

E.1 $$\begin{aligned} \left[ {{\mathcal {O}}}_x^{(C_2;h_a;1,0)},\, {{\mathcal {O}}}_x^{(C_2;h_b;0,1)}\right] =0,\quad \forall ~ ~ a,b=1,\ldots ,10. \end{aligned}$$

Using explicit expression (3.17), we can write out all the terms of the commutator:

E.2 $$\begin{aligned}{} & {} \left[ {{\mathcal {O}}}_x^{(C_2;h_a;1,0)},\, {{\mathcal {O}}}_x^{(C_2;h_b;0,1)}\right] {{\mathcal {I}}}(x) \nonumber \\{} & {} \quad =\sum \limits _{i,j=1}^2\left[ A_i^{\left( C_2;h_a;1,0\right) }(x_i) \left( \Delta _q(x_i) A_j^{\left( C_2;h_b;0,1\right) }(x) \right) \Delta _q(x_i)\Delta _p(x_j) {{\mathcal {I}}}(x_i, x_j) \right. \nonumber \\{} & {} \qquad +A_i^{\left( C_2;h_a;1,0\right) }(x)\left( \Delta _q(x_i) A_j^{\left( C_2;h_b;0,1\right) }\left( x^{-1}\right) \right) \Delta _q(x_i)\Delta _p^{-1}(x_j) {{\mathcal {I}}}(x) \nonumber \\{} & {} \qquad +A_i^{\left( C_2;h_a;1,0\right) }\left( x^{-1}\right) \left( \Delta ^{-1}_q(x_i) A_j^{\left( C_2;h_b;0,1\right) }(x) \right) \Delta ^{-1}_q(x_i)\Delta _p(x_j) {{\mathcal {I}}}(x) \nonumber \\{} & {} \qquad +\left. A_i^{\left( C_2;h_a;1,0\right) }\left( x^{-1}\right) \left( \Delta ^{-1}_q(x_i) A_j^{\left( C_2;h_b;0,1\right) }\left( x^{-1}\right) \right) \Delta ^{-1}_q(x_i)\Delta ^{-1}_p(x_j) {{\mathcal {I}}}(x)\right] \nonumber \\{} & {} \qquad +\sum \limits _{i=1}^2\left[ A_i^{\left( C_2;h_a;1,0\right) }(x)\left( \Delta _q(x_i)W^{\left( C_2;h_b;0,1\right) }(x)-W^{\left( C_2;h_b;0,1\right) }(x)\right) \Delta _q(x_i) {{\mathcal {I}}}(x) \right. \nonumber \\{} & {} \qquad +\left. A_i^{\left( C_2;h_b;0,1\right) }\left( x^{-1}\right) \left( \Delta ^{-1}_p(x_i)W^{\left( C_2;h_a;1,0\right) }(x)-W^{\left( C_2;h_a;1,0\right) }(x)\right) \Delta ^{-1}_q(x_i) {{\mathcal {I}}}(x)\right] \nonumber \\{} & {} \qquad -\left( \begin{array}{c} p\leftrightarrow q \\ a \leftrightarrow b \end{array} \right) , \end{aligned}$$

where in the last line we subtract all the terms written in the first six lines but with *p* and *q* parameters as well as *a* and *b* indices exchanged. Now in order to compute this commutator, we should find the action of the shift operators $\Delta _q(x_i)$ and $\Delta _p(x_i)$ on the shift part $A_i(x)$ and constant part *W*(*x*). Using explicit expressions from (3.17), we obtain:

E.3 $$\begin{aligned} \Delta _p(x_i)W^{(C_2;h_a;1,0)}(x)= & {} W^{(C_2;h_a;1,0)}(x), \nonumber \\ \Delta _p(x_i)A_j^{(C_2;h_a;1,0)}(x)= & {} \Delta _p(x_i)^{-1}A_j^{(C_2;h_a;1,0)}(x)=A_j^{(C_2;h_a;1,0)}(x), \nonumber \\ \Delta _p(x_i)A_i^{(C_2;h_a;1,0)}(x)= & {} (pq)^{-3}hA_i^{(C_2;h_a;1,0)}(x), \nonumber \\ \Delta ^{-1}_p(x_i)A_i^{(C_2;h_a;1,0)}(x)= & {} (pq)^{3}h^{-1}A_i^{(C_2;h_a;1,0)}(x), \end{aligned}$$

where *h* in the last expression is as usually total *U*(1) charge defined in (3.16). Completely identical expressions can be written for *p* and *q* exchanged. Using these expressions for the commutator action (E.2), it is straightforward to see that it is just zero:

E.4 $$\begin{aligned} \left[ {{\mathcal {O}}}_x^{(C_2;h_a;1,0)},\, {{\mathcal {O}}}_x^{(C_2;h_b;0,1)}\right] {{\mathcal {I}}}(x)=0. \end{aligned}$$

Since the test function ${{\mathcal {I}}}(x)$ is arbitrary, we can conclude that the commutator itself is also zero.

Now, let’s move to the computation of more complicated type of commutators

E.5 $$\begin{aligned} \left[ {{\mathcal {O}}}_x^{(C_2;h_a;1,0)},\, {{\mathcal {O}}}_x^{(C_2;h_b;1,0)}\right] =0, \quad \forall ~ ~ a,b=1,\ldots ,10. \end{aligned}$$

In this case, the proof is more complicated since the periodicity properties of $\theta$ functions cannot be used anymore. Instead we will be using expansion in *p* and *q* parameters to perform this check. Once again we will use action of the commutator on an arbitrary test function ${{\mathcal {I}}}(x)$:

E.6 $$\begin{aligned}{} & {} \left[ {{\mathcal {O}}}_x^{(C_2;h_a;1,0)},\, {{\mathcal {O}}}_x^{(C_2;h_b;1,0)}\right] {{\mathcal {I}}}(x) \nonumber \\{} & {} \quad =\sum \limits _{i,j=1}^2\left[ A_i^{\left( C_2;h_a;1,0\right) }(x_i) \left( \Delta _q(x_i) A_j^{\left( C_2;h_b;1,0\right) }(x) \right) \Delta _q(x_i)\Delta _p(x_j) {{\mathcal {I}}}(x_i, x_j) \right. \nonumber \\{} & {} \qquad +A_i^{\left( C_2;h_a;1,0\right) }(x)\left( \Delta _q(x_i) A_j^{\left( C_2;h_b;1,0\right) }\left( x^{-1}\right) \right) \Delta _q(x_i)\Delta _q^{-1}(x_j) {{\mathcal {I}}}(x) \nonumber \\{} & {} \qquad +A_i^{\left( C_2;h_a;1,0\right) }\left( x^{-1}\right) \left( \Delta ^{-1}_q(x_i) A_j^{\left( C_2;h_b;1,0\right) }(x) \right) \Delta ^{-1}_q(x_i)\Delta _q(x_j) {{\mathcal {I}}}(x) \nonumber \\{} & {} \qquad +\left. A_i^{\left( C_2;h_a;1,0\right) }\left( x^{-1}\right) \left( \Delta ^{-1}_q(x_i) A_j^{\left( C_2;h_b;1,0\right) }\left( x^{-1}\right) \right) \Delta ^{-1}_q(x_i)\Delta ^{-1}_q(x_j) {{\mathcal {I}}}(x)\right] \nonumber \\{} & {} \qquad +\sum \limits _{i=1}^2\left[ A_i^{\left( C_2;h_a;1,0\right) }(x)\left( \Delta _q(x_i)W^{\left( C_2;h_b;1,0\right) }(x)-W^{\left( C_2;h_b;1,0\right) }(x)\right) \Delta _q(x_i) {{\mathcal {I}}}(x) \right. \nonumber \\{} & {} \qquad +\left. A_i^{\left( C_2;h_b;1,0\right) }\left( x^{-1}\right) \left( \Delta ^{-1}_q(x_i)W^{\left( C_2;h_a;1,0\right) }(x)-W^{\left( C_2;h_a;1,0\right) }(x)\right) \Delta ^{-1}_q(x_i) {{\mathcal {I}}}(x)\right] \nonumber \\{} & {} \qquad -\left( \begin{array}{c} a \leftrightarrow b \end{array} \right) , \end{aligned}$$

Since the test function ${{\mathcal {I}}}(x)$ is arbitrary in order to perform our checks, we have to consider contributions of all possible shifts of ${{\mathcal {I}}}(x)$ separately. Below we discuss such contributions one by one.

*(1) Terms with* $\Delta _q(x_i)\Delta _q(x_j) {{\mathcal {I}}}(x)$. This kind of contributions in (E.6) comes from the following terms:

E.7 $$\begin{aligned}{} & {} \left[ {{\mathcal {O}}}_x^{(C_2;h_a;1,0)},\, {{\mathcal {O}}}_x^{(C_2;h_b;1,0)}\right] {{\mathcal {I}}}(x)\sim \left[ A_i^{\left( C_2;h_a;1,0\right) }(x)\left( \Delta _q(x_i)A_j^{\left( C_2;h_b;1,0\right) }(x)\right) \right. \nonumber \\{} & {} \quad +\left. A_j^{\left( C_2;h_a;1,0\right) }(x)\left( \Delta _q(x_j)A_i^{\left( C_2;h_b;1,0\right) }(x)\right) -\left( a\leftrightarrow b \right) \right] \Delta _q(x_i)\Delta _q(x_j) {{\mathcal {I}}}(x).\nonumber \\ \end{aligned}$$

In order for the prefactor to be zero, the following algebraic identity has to be satisfied:

E.8 $$\begin{aligned}{} & {} F_1(x_i,x_j,h_a,h_b)+F_1(x_j,x_i,h_a,h_b)-F_1(x_i,x_j,h_b,h_a)-F_1(x_j,x_i,h_b,h_a)=0, \nonumber \\{} & {} F_1(x_i,x_j,h_a,h_b) \nonumber \\{} & {} \qquad =\frac{\theta _p\left( x_jx_i^{\pm 1}\right) \theta _p\left( (pq)^{\frac{1}{2}}h_bqx_i\right) \theta _p\left( (pq)^{\frac{1}{2}}h_bq^{-1}x_i^{-1}\right) \theta _p\left( (pq)^{\frac{1}{2}}h_ax_j^{\pm 1}\right) }{\theta _p\left( qx_jx_i\right) \theta _p\left( q^{-1}x_jx_i^{-1}\right) }. \end{aligned}$$

In order to check this equation, we expand all functions in *p* and *q* parameters. Since there are just two variables $x_1$ and $x_2$ in this case, we can fix $i=1$ and $j=2$ without loss of generality and check equality in expansion up to the order $O\left( p^3q^3\right)$. This check suggests that indeed these kind of terms do not contribute to the commutator (E.6).

*(2) Terms with* $\Delta ^2_q(x_i) {{\mathcal {I}}}(x)$. This contribution comes from the following terms:

E.9 $$\begin{aligned}{} & {} \left[ {{\mathcal {O}}}_x^{(C_2;h_a;1,0)},\, {{\mathcal {O}}}_x^{(C_2;h_b;1,0)}\right] {{\mathcal {I}}}(x)\sim \left[ A_i^{\left( C_2;h_a;1,0\right) }(x)\left( \Delta _q(x_i)A_i^{\left( C_2;h_b;1,0\right) }(x)\right) \right. \nonumber \\{} & {} \quad -\left. A_i^{\left( C_2;h_b;1,0\right) }(x)\left( \Delta _q(x_i)A_i^{\left( C_2;h_a;1,0\right) }(x)\right) \right] \Delta ^2_q(x_i) {{\mathcal {I}}}(x). \end{aligned}$$

We can now notice that

E.10 $$\begin{aligned} \Delta _q(x_i)A_i^{\left( C_2;h_a;1,0\right) }(x)= & {} \prod \limits _{j\ne i}^2\frac{\theta _p\left( x_i^2\right) \theta _p\left( qx_i^2\right) \theta _p\left( x_ix_j^{\pm 1}\right) }{\theta _p\left( q^2x_i^2\right) \theta _p\left( q^3x_i^2\right) \theta _p\left( qx_ix_j^{\pm 1}\right) } \nonumber \\{} & {} \times \prod \limits _{l=1}^{10}\frac{\theta _p\left( (pq)^{\frac{1}{2}}h_l^{-1}qx_i\right) }{\theta _p\left( (pq)^{\frac{1}{2}}h_l^{-1}x_i\right) }A_i^{\left( C_2;h_a;1,0\right) }(x), \end{aligned}$$

so that the overall factor we get after acting with the shift $\Delta _q(x_i)$ does not depend on the index *a*. Due to this independence, it is clear that corresponding contribution to the commutator (E.6) is zero.

*(3) Terms with* $\Delta _q(x_i)\Delta _q^{-1}(x_j) {{\mathcal {I}}}(x)$. These terms come from the contribution:

E.11 $$\begin{aligned}{} & {} \left[ {{\mathcal {O}}}_x^{(C_2;h_a;1,0)},\, {{\mathcal {O}}}_x^{(C_2;h_b;1,0)}\right] {{\mathcal {I}}}(x)\sim \left[ A_i^{\left( C_2;h_a;1,0\right) }(x)\left( \Delta _q(x_i)A_j^{\left( C_2;h_b;1,0\right) }\left( x^{-1}\right) \right) \right. \nonumber \\{} & {} \quad +\left. A_j^{\left( C_2;h_a;1,0\right) }\left( x^{-1}\right) \left( \Delta ^{-1}_q(x_j)A_i^{\left( C_2;h_b;1,0\right) }(x)\right) -(a\leftrightarrow b) \right] \Delta _q(x_i)\Delta _q^{-1}(x_j) {{\mathcal {I}}}(x).\nonumber \\ \end{aligned}$$

In order for this term to vanish, we need the following equation to be satisfied:

E.12 $$\begin{aligned}{} & {} F_2(x_i,x_j,a,b)-F_2(x_i,x_j,b,a)-F_2(x_j^{-1},x_i^{-1},b,a)+F_2(x_j^{-1},x_i^{-1},a,b)=0, \nonumber \\{} & {} F_2(x_i,x_j,a,b) \nonumber \\{} & {} \quad =\frac{\theta _p\left( x_j^{-1}x_i^{\pm 1}\right) \theta _p\left( (pq)^{\frac{1}{2}}h_bqx_i\right) \theta _p\left( (pq)^{\frac{1}{2}}h_bq^{-1}x_i^{-1}\right) \theta _p\left( (pq)^{\frac{1}{2}}h_ax_j^{\pm 1}\right) }{\theta _p\left( qx_ix_j^{-1}\right) \theta _p\left( q^{-1}x_i^{-1}x_j^{-1}\right) }\nonumber \\ \end{aligned}$$

Just as previously, we check this identity in *p* and *q* expansion up to an order of $O\left( p^3q^3\right)$ and thus show that corresponding contribution to the commutator is zero.

*(4) Terms with* $\Delta _q(x_i) {{\mathcal {I}}}(x)$. This kind of terms comes from the contribution:

E.13 $$\begin{aligned}{} & {} \left[ {{\mathcal {O}}}_x^{(C_2;h_a;1,0)},\, {{\mathcal {O}}}_x^{(C_2;h_b;1,0)}\right] {{\mathcal {I}}}(x)\sim \left[ A_i^{\left( C_2;h_a;1,0\right) }(x)\left( \Delta _q(x_i)W^{\left( C_2;h_b;1,0\right) }\left( x\right) \right) \right. \nonumber \\{} & {} \quad +\left. W^{\left( C_2;h_a;1,0\right) }\left( x\right) A_i^{\left( C_2;h_b;1,0\right) }\left( x\right) -(a\leftrightarrow b) \right] \Delta _q(x_i) {{\mathcal {I}}}(x). \end{aligned}$$

This expression is hard to simplify so we checked it directly fixing $i=1,j=2$ and expanding up to an order $O\left( p^2q^0 \right)$, $O\left( p^0q^2 \right)$ and $O\left( pq\right)$ in *p* and *q* parameters. Up to these orders in expansion, we confirmed that corresponding contribution to the commutator is zero.

*(5) Constant terms* ${{\mathcal {I}}}(x)$. This term comes from the contribution of

E.14 $$\begin{aligned}{} & {} \left[ {{\mathcal {O}}}_x^{(C_2;h_a;1,0)},\, {{\mathcal {O}}}_x^{(C_2;h_b;1,0)}\right] {{\mathcal {I}}}(x)\sim \left[ W^{\left( C_2;h_a;1,0\right) }(x)W^{\left( C_2;h_b;1,0\right) }\left( x\right) \right. \nonumber \\{} & {} \quad -\left. W^{\left( C_2;h_b;1,0\right) }(x)W^{\left( C_2;h_a;1,0\right) }\left( x\right) \right] {{\mathcal {I}}}(x)=0. \end{aligned}$$

This term is obviously zero since it does not involve any shift.

*(6) Terms with* $\Delta _q^{-1}(x_i)\Delta _q^{-1}(x_j) {{\mathcal {I}}}(x)$. These terms can be directly obtained from Type 1 terms containing $\Delta _q(x_i)\Delta _q(x_j) {{\mathcal {I}}}(x)$ by $x_i\rightarrow x_i^{-1}$ transformation of variables. Due to the $x_i\rightarrow x_i^{-1}$ symmetry of A$\Delta$O (3.17), we can immediately conclude that corresponding contribution to the commutator action (E.6) is zero.

*(7) Terms with* $\Delta _q^{-2}(x_i) {{\mathcal {I}}}(x)$. These terms can be directly obtained from Type 2 terms containing $\Delta ^2_q(x_i) {{\mathcal {I}}}(x)$ by $x_i\rightarrow x_i^{-1}$ transformation of variables. Hence by the same symmetry argument as in the previous case, we can immediately conclude that corresponding contribution to the commutator action (E.6) is zero.

*(8) Terms with* $\Delta _q^{-1}(x_i) {{\mathcal {I}}}(x)$. Contributions to the commutator action (E.6) of this type are also zero which follows from the same argument as previous two terms. In this case, it can be obtained by using $x_i\rightarrow x_i^{-1}$ transformation in Type 4 terms.

Thus, all eight types of terms present in the commutator (E.6) give zero contribution and the full commutator action, and hence, the commutator itself appears to be zero. So far we have shown it only in *p* and *q* expansion. But in principle this can be also proven analytically by computing positions of the poles and corresponding residues of all equalities we have checked in expansion. This way of proof is similar to the one we used in Appendix D to prove kernel property (4.2).

The last type of commutator identities we would like to prove is

E.15 $$\begin{aligned} \left[ {{\mathcal {O}}}_x^{(C_2;h_a;0,1)},\, {{\mathcal {O}}}_x^{(C_2;h_b;0,1)}\right] =0,\quad \forall ~ ~a,b=1,\ldots ,10. \end{aligned}$$

But this commutator can be directly obtained from the previous commutator

$\left[ {{\mathcal {O}}}_x^{(C_2;h_a;1,0)},\, {{\mathcal {O}}}_x^{(C_2;h_b;1,0)}\right]$ by the exchange $p\leftrightarrow q$. Hence, all the arguments above work also for this commutator which as result is indeed equal to zero.

To summarize, in this Appendix we gave arguments in favor of all commutation relations (4.5). For the third commutator, we gave full analytic proof, while for the first and second we checked commutation relations perturbatively in *p* and *q* expansion to sufficiently high order.

## Footnotes

1 The simplest case of $N=1$, as well as the case of $(A_1)^1$, was studied in our previous paper [19]. As a result, we observed two alternative parametrizations of the standard $BC_1$ vD model.

2 We would like to stress that the operator we derive is different from the standard higher-rank vD operator [12, 13] since the latter one is associated with the affine $BC_n$-type root system, while we discuss $C_2$ root system in our paper. We expect that the canonical $BC_n$ vD model can be obtained in a similar manner using compactifications of the rank-Q E-string theory. Unfortunately so far only compactifications on the spheres with two punctures are known for these theories so we are not able to prove this conjecture.

3 See the definition of the basic operators and their towers below.

4 Here and further, we often omit some overall factors which are irrelevant for the derivations of A$\Delta$Os. Because of this, we use $\sim$ instead of strict equality here.

5 Similar comments hold for the previously discussed $A_N$ operators [19], which depend on *p*, *q* and another $2N+4$ parameters.
